# Genome-wide CRISPR screen identifies BUB1 kinase as a druggable vulnerability in malignant pleural mesothelioma

**DOI:** 10.1038/s41419-025-07587-z

**Published:** 2025-04-03

**Authors:** Ece Cakiroglu, Sude Eris, Ozden Oz, Gökhan Karakülah, Serif Senturk

**Affiliations:** 1 Izmir Biomedicine and Genome Center, Izmir, Turkey; 2 Izmir International Biomedicine and Genome Institute, Dokuz Eylul University, Izmir, Turkey; 3 Department of Pathology, Izmir Bozyaka Education and Research Hospital, University of Health Sciences, Izmir, Turkey

**Keywords:** Mesothelioma, Cancer genomics, Mechanisms of disease

## Abstract

Malignant pleural mesothelioma (MPM) is a rare yet highly aggressive malignancy with a severe prognosis. Compounded by the lack of effective treatment modalities, MPM remains a formidable health challenge. Therefore, the identification of actionable liabilities is critical for advancing precision medicine to combat this lethal disease. Here, we exploit an unbiased genome-wide CRISPR screen, integrating and cross-comparing three MPM cell lines with nonmalignant mesothelial cells, to selectively map the gene targets whose depletion indicates a common dependency in MPM cells. This systematic approach unveils a cohort of verifiable genes, among which BUB1, a mitotic checkpoint serine/threonine kinase, emerges as a high-confidence hit in cancer cells. Cellular and molecular studies demonstrate that genetic depletion or pharmacological inhibition of BUB1 profoundly impairs MPM cell survival and growth while inducing G2/M cell cycle arrest, cellular senescence, and apoptosis, and attenuating functional hallmarks of aggressive cancer cells. Transcriptomic profiling of BUB1-depleted cells discloses differential gene expression signatures congruent with cell fate phenotypes, including the reprogramming of mitotic network genes. Mechanistically, BUB1 is indispensable for the proper localization of essential mitotic regulators MAD1, MAD2, and Shugoshin (SGO1), thereby ensuring the functionality of the spindle assembly checkpoint (SAC). Furthermore, BUB1 ablation leads to cytokinesis failure and multinucleation, a phenotype characterized by the downregulation of CDC20, Cyclin A, and Cyclin B, and a reciprocal upregulation of the cyclin-dependent kinase inhibitor p21. Clinically, MPM tumors exhibit elevated levels of BUB1, and high BUB1 expression is associated with shorter patient survival. Our novel findings accentuate comparative CRISPR screens as a powerful platform to explore tumor cell-selective gene essentiality and propose BUB1 kinase as a potential marker and druggable vulnerability with therapeutic implications for MPM.

## Introduction

Malignant pleural mesothelioma (MPM) originates from mesothelial cells lining the pleural cavity and is typically associated with asbestos exposure. Although relatively rare, MPM constitutes ~80% of all mesotheliomas and stands out as one of the most lethal malignancies, with an average survival time of 1 year after diagnosis [1]. Recent statistics report nearly 3000 new cases in the United States [2], contributing to a global incidence of around 30,000 new patients, and more than 26,000 recorded deaths globally in 2020 [1]. Comprehensive genomic studies have significantly enhanced the understanding of MPM biology, revealing recurrent chromosomal alterations, often manifested as point mutations and copy number changes across multiple genomic regions. Notably, unlike many cancers characterized by the presence of druggable oncogenic driver alterations, MPM carcinogenesis is predominantly driven by loss of tumor suppressor gene function [3].

Despite advancements in medical oncology, MPM remains largely refractory to conventional frontline therapies [4]. Efforts to develop a comprehensive and effective treatment are still in progress. Current strategies focus on small-molecule inhibitors or monoclonal antibodies targeting pathways such as PI3K/mTOR, FGFR, and EGFR, and biological processes related to immune checkpoint modulation and angiogenesis. Unfortunately, clinical trials exploring these avenues have thus far demonstrated limited efficacy [5, 6]. Given these challenges, there is a pressing need for ongoing research to identify novel targets and enhance the precision medicine arsenal for MPM.

High-throughput functional genomics has been instrumental in advancing our understanding of the genetic and molecular characteristics of cancer, enabling the discovery of potential therapeutic vulnerabilities across various cancer types [7]. In this context, pooled genome-wide or focused CRISPR screening has proven to be a powerful strategy for mapping genetic determinants of cancer cell fitness [8]. In this study, we leverage an unbiased genome-wide CRISPR screening approach, utilizing the Brunello library across multiple cell lines. The primary objective is to systematically identify gene landscapes relevant to MPM and unearth therapeutically actionable genes. Target identification involves a cross-comparative analysis between three MPM cell lines (H2052, H2452, and H28) and a nonmalignant mesothelial cell line (MeT-5A). We focus on identifying hits with shared negative CRISPR viability scores in MPM cell lines while confining MeT-5A to minimum or absent CRISPR effects. This strategy unveils *ATG9A*, *BUB1*, *CCND1*, *CDK2*, *PIKFYVE*, *TMEM41B*, *USP17L25*, and *VPS37A* as high-confidence genes, among which are known liabilities. Individual validation of top hits with targeted genetic depletions demonstrates the robust performance of our CRISPR screening platform. We prioritize budding uninhibited by benzimidazoles 1 (BUB1), a mitotic G2/M checkpoint serine/threonine kinase [9], as a unique druggable vulnerability in MPM pathophysiology. Genetic depletion or pharmacological inhibition of BUB1 in MPM cells attenuates DNA synthesis, impairs cell survival and growth, and is associated with G2/M cell cycle arrest, cellular senescence, and apoptosis. Consistent with these effects, BUB1 depletion mitigates functional hallmarks of cancer cells and inhibits tumor-relevant phenotypes in vitro. At the molecular level, BUB1 inactivation elicits transcriptomic reprogramming of mitotic regulatory circuits, leading to the attenuation of proliferative gene expression. Furthermore, BUB1 is essential for proper SAC function associated with localization of MAD1, MAD2, and SGO1, and its depletion disrupts cytokinesis, resulting in multinucleated cells, a phenotype defined by reduced expression of CDC20, Cyclin A2, and Cyclin B1, coupled with an increased expression of p21. Critically, elevated BUB1 levels in MPM tumors predict poor overall survival. These findings propose BUB1 as a novel potential marker and molecular target in MPM. Furthermore, our study underscores the broadly applicable genome-wide CRISPR screen platform for unbiased exploration of functional phenotypes and identification of drug-specific targets for precision medicine in MPM.

## Results

### Genome-wide CRISPR screens in MPM cell lines and normal cells

To interrogate cancer dependencies in MPM, we conducted an unbiased genome-wide loss-of-function genetic screen using the Brunello pooled library of 77,441 gRNAs [10]. The screen was performed across three human MPM cell lines representing all histologic subtypes: sarcomatoid (H2052), biphasic (H2452), and epithelioid (H28) [11], and an SV40 immortalized normal (nonmalignant) pleural mesothelial cell line with an epithelial phenotype (MeT-5A) [12]. The overarching objective of this comparative, multi-cell line CRISPR screening approach was to decipher vulnerabilities exclusive to MPM cells.

**Fig. 1:**
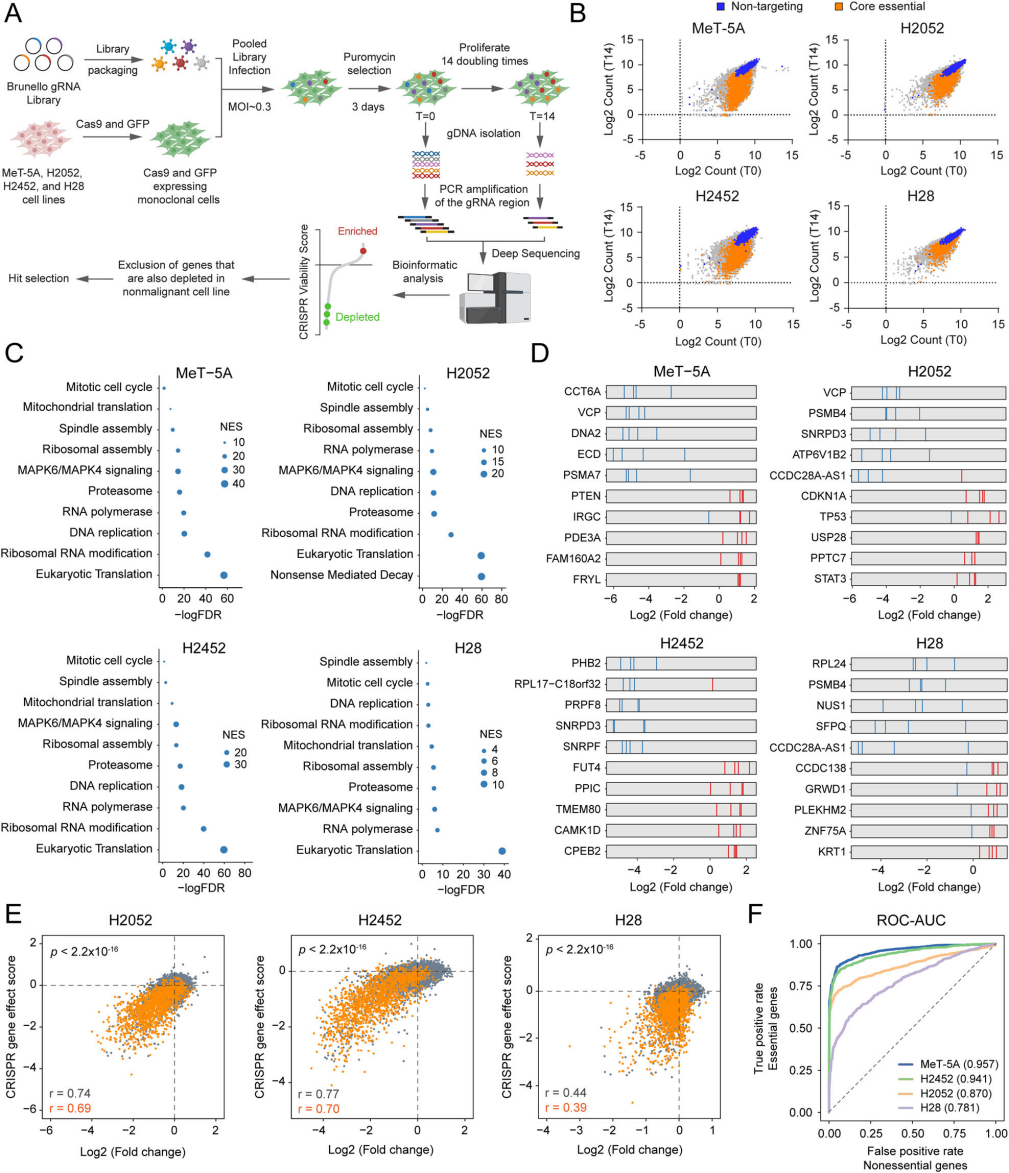
CRISPR screening of MPM and nonmalignant cell lines. **A** Schematic workflow of genome-wide loss-of-function CRISPR screens conducted in MeT-5A, H2052, H2452, and H28 cell lines. Cas9-expressing monoclonal cell lines were transduced with Brunello library at MOI ≌ 0.3 and selected with puromycin. Genomic DNA was extracted from T0 and T14 cell pellets. gRNA-targeted regions were amplified by PCR and sequenced on the HiSeq2500 system. Data were analyzed using MAGeCK software. High-confidence hits were identified based on negative CRISPR viability scores (depleted). **B** The dot plot showing the distributions of log2 normalized T14 gRNA read counts relative to T0 gRNA read counts. gRNAs targeting 360 core essential genes [88] (orange dots) and non-targeting gRNAs (blue dots), negative controls in the Brunello library, are shown. **C** Cellular and molecular pathways involving the genes with the highest depletion scores in the CRISPR screens were obtained by pathway enrichment analysis. **D** The log2 fold change of gRNAs for top-ranked and bottom-ranked 5 genes is depicted. The gRNAs with negative log2 fold change were represented as blue lines and the ones with positive log2 fold change were represented as red lines. **E** Correlation between our genome-wide CRISPR screen and CRISPR knockout screen data from the Project Achilles (DepMap Public 23Q2+Score, Chronos, access date: 09.06.2023). Genome-wide targets (gray) and fitness/essential genes (1580, orange) are highlighted. **F** ROC-AUC analysis of CRISPR knockout screening data across four cell lines. False positive rates are calculated based on nonessential genes and graphed in relation to the true positive rate, which is determined by essential genes.

To ensure genetic and cellular homogeneity for CRISPR screens, we engineered stable Cas9-expressing clonal cell lines by transducing cells with Flag-tagged Cas9 fused to EGFP (Fig. S1A). Cell pools were processed to establish isogenic clones (Fig. S1B, C) and Cas9 activity was verified by a competitive cell proliferation assay against the essential gene *RPA3* and the T7EI mismatch detection assay (Fig. S1D, E). Additionally, the Brunello gRNA library was amplified, achieving uniform distribution (Fig. S1F). Following CRISPR screens (Fig. 1A), genomic DNA from T0 and T14 samples was PCR-amplified, barcoded, and sequenced by NGS. Relative gRNA abundance was determined using MAGeCK software [13], and gene scores were calculated as the median log2 fold change of all gRNAs targeting the gene. Quality metrics such as gRNA distribution and Gini index confirmed consistency across samples (Fig. S2A–C). Furthermore, analysis of gRNAs targeting a set of 1580 human fitness/essential genes [14] showed significant depletion across all four cell lines (Fig. S2D). Unlike the depletion of 360 core essential genes (orange), the distribution of non-targeting gRNAs (blue) remained largely unchanged between T0 and T14 populations (Fig. 1B). Pathway enrichment analysis of the most significantly depleted genes using MAGeCKFlute algorithm revealed strong enrichment of essential biological processes (Fig. 1C). Similarly, functional annotation of the top 200 lethal genes using the DAVID database [15] elucidated molecular components involved in cell cycle, ribosomal function, RNA transport, and purine and pyrimidine metabolism (Fig. S2E). Five top-ranked depleted and enriched genes were highlighted in Fig. 1D, demonstrating consistent negative and positive selection for the majority of gRNAs. To validate our findings, we performed a comparative correlation coefficient analysis using genome-scale CRISPR screen data for MPM cell lines from Project Achilles (DepMap Public 23Q2+Score, Chronos). Except for a moderate correlation in the H28 cell line (*r* ≌ 0.4, *p* value < 2.2 × 10^−16^), we observed strong linear correlations (*r* > 0.6, *p* value < 2.2 × 10^−16^) between datasets for all genes (gray) and the subset of 1580 fitness genes (orange) (Fig. 1E). We further inferred the efficacy of CRISPR screens using ROC-AUC values to distinguish between essential (1580, true positives) and nonessential genes (927, false positive) [14]. These data indicated robust screen performance in MeT-5A, H2052, and H2452 cell lines, with a relatively lower efficacy in H28 cells (Fig. 1F). Thus, the adopted CRISPR screening strategy may serve as an effective tool tailored to identify MPM-relevant vulnerabilities.

### Validation of high-confidence CRISPR screening hits

**Fig. 2:**
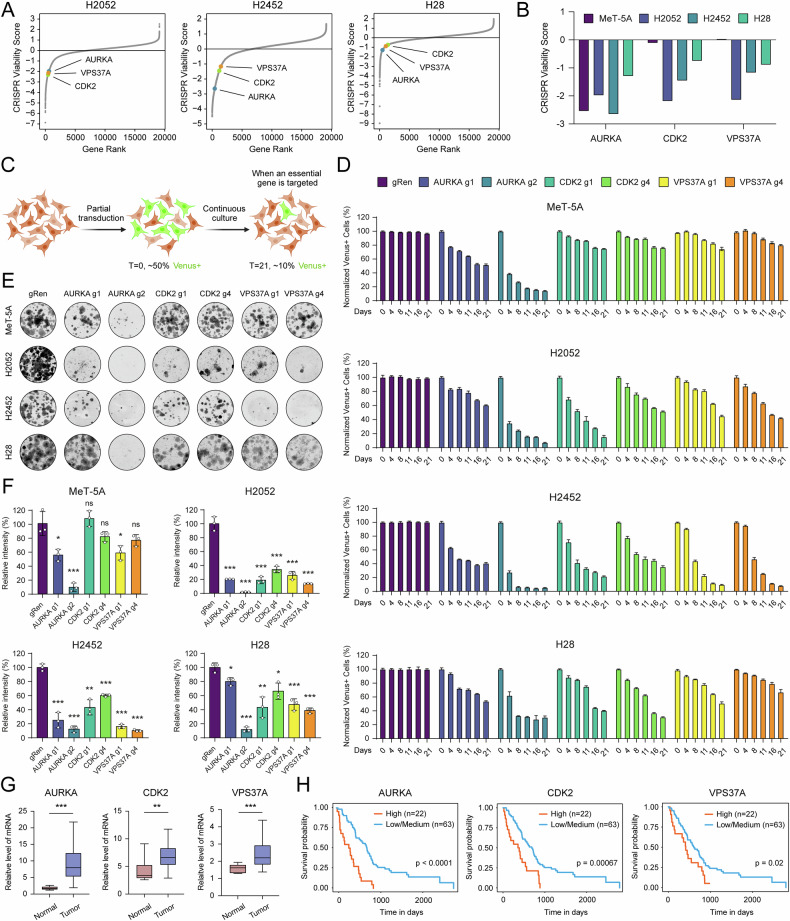
Validation of high-confidence CRISPR screening hits. **A** Dot plots indicate CRISPR viability scores of all genes (gray dots) and significantly depleted *AURKA*, *CDK2*, and *VPS37A* genes (colored dots). **B** Chart visualization of CRISPR viability scores for *AURKA*, *CDK2*, and *VPS37A* genes in MeT-5A, H2052, H2452, and H28 cell lines. **C** Schematic illustration of competitive cell proliferation assay. Partially transduced cells are examined for Venus positivity in a time-dependent manner. A significant decrease in the percentage of Venus-positive cells infected with gRNA indicates prominent cell depletion over time, implicating the essential role of the targeted gene. **D** Competitive cell proliferation assay results of MeT-5A, H2052, H2452, and H28 cells infected with gRNAs targeting *AURKA*, *CDK2*, and *VPS37A* genes. The percentage of Venus-positive cells at Day 0 was normalized to 100%, and the following measurements were calculated accordingly. Bar graphs are presented as the mean ± SD of three replicates. **E** Representative images showing decreased 2D colony formation capacity of MeT-5A, H2052, H2452, and H28 cell lines upon AURKA, CDK2, and VPS37A depletion. Colony formation assay was performed in triplicates in 12-well cell culture plates for 10–14 days. High-resolution images of the plates were acquired by LI-COR Odyssey CLx Imaging System. **F** Crystal violet intensity data showing the relative difference in 2D colony formation capacity of gRen and AURKA, CDK2, and VPS37A gRNA expressing cells. Image Studio software was used to measure signal intensities. Bar graphs are presented as the mean ± SD of three replicates. Two-tailed Student’s *t*-test was used for statistical analysis. *p < 0.05, **p < 0.01, ***p < 0.001, ns not significant. **G** Box–Whisker plot of AURKA, CDK2, and VPS37A mRNA expression levels in MPM (*n* = 39) and normal tissues (*n* = 9) from GSE42977 dataset. Statistical significance was calculated by a two-tailed Mann–Whitney test. **p < 0.01, ***p < 0.001. **H** The Kaplan–Meier survival plots for *AURKA*, *CDK2*, and *VPS37A* genes indicate survival rates of MPM patients (the TCGA MPM dataset) whose tumors have high gene expression (orange) relative to the low/medium patients (blue). *p* values are shown on the plots.

To verify the specificity of this platform in revealing cell-type-specific gene dependencies, we constructed CRISPR viability scores for all genes in the library. Top scoring hits with non-discriminatory nature across all four cell lines included common essential genes [16] with known fitness functions in fundamental cellular processes, such as DNA replication (*PCNA*, *POLE2*), regulation of cell cycle (*ANAPC1*, *AURKA*, *BUB3*, *CDC7*), transcription (*MED11*, *POLR2L*, *POLR3A*), spliceosome formation (*SF3B5*, *SFPQ*, *SNRPF*, *WDR77*), ribosome biogenesis (*FAU*, *RPL11*, *RPL17*, *RPL24*), protein transport (*WASH1*, *XPO1*), and telomere protection (*TERF2*) (Fig. S3A). To systematically uncover MPM-relevant vulnerabilities, we conducted a comparative analysis, ensuring that the gRNA responses in the nonmalignant cell line were not more pronounced than in the three cancer cell lines. This hit filtering strategy allowed for flexible MeT-5A cell line response to the gRNAs targeting the gene of interest while confining MPM cell lines to depletion of the same gRNAs relative to normal cells. Through this approach, we identified eight high-confidence genes (*ATG9A*, *BUB1*, *CCND1*, *CDK2*, *PIKFYVE*, *TMEM41B*, *USP17L25*, *VPS37A*) scored by at least two gRNAs (Fig. S3B). CRISPR viability scores of these genes exhibited negative enrichment in cancer cells (Fig. S3C). For molecular validation, we selected *AURKA* as a control gene, expecting non-discriminatory effects between cancer and normal cells (Figs. 2A, B and S3A). Despite its pronounced depletion in MeT-5A cells, likely due to its inherent nature as a core essential gene, AURKA dysregulation has been implicated in a wide range of cancers with several inhibitors targeting its oncogenic activity [17–19]. Our focus also extended to *CDK2* and *VPS37A* genes, both of unknown biological significance in MPM, whose depletion by CRISPR should selectively affect cancer cells (Figs. 2A, B and S3C). Notably, CDK2 is a clinically relevant druggable liability strongly associated with tumor growth in multiple cancers [20–22]. In contrast to our screening results, existing data on VPS37A suggest its downregulation as a tumor promoter and an unfavorable prognostic indicator in breast and gastric cancers [23, 24], indicating a potential MPM-specific role.

To dissect the effects of genetic perturbation, we employed a Venus-expressing CRISPR knockout vector [25]. Cells were targeted with selected gRNAs based on their scores (Fig. S3D), and Venus-positive cells were tracked via a competitive cell proliferation assay (Fig. 2C). As expected, AURKA ablation significantly inhibited proliferation in all cell lines, whereas CDK2 and VPS37A knockout selectively suppressed growth in cancer cells (Fig. 2D). Further confirmation was achieved by establishing CRISPR knockout clones (Fig. S3E). As shown in Fig. 2E, F, cells expressing gRNAs against AURKA exhibited significantly impaired colony formation in all cell lines. In contrast, CDK2 and VPS37A knockout induced growth disadvantage in MPM cell lines, with less pronounced effects in H28 cells (Fig. 2B). From a generalizability standpoint, we integrated gene essentiality data from Project Achilles and CCLE RNA-seq profiles for each hit. This analysis revealed concordant gene essentiality and constitutive high expression with low variance across all MPM cell lines (Fig. S3F, G). Having shown that targeting AURKA, CDK2, and VPS37A is indispensable for MPM cell proliferation and growth, we next investigated the significance of these genes from a clinical perspective. Gene expression analysis of the GSE42977 dataset revealed significant upregulation of AURKA, CDK2, and VPS37A in MPM tumor tissues compared to normal mesothelial and lung tissues (Fig. 2G). More significantly, UALCAN analysis of The Cancer Genome Atlas (TCGA) MPM data indicated that high expression levels of these genes correlate with significantly shorter overall patient survival (Fig. 2H). Therefore, AURKA, CDK2, and VPS37A potentially play critical roles in MPM progression or therapy response, making them promising candidates for predictive biomarkers and therapeutic targets. These findings strongly validate the hit discovery potential of our CRISPR screening approach.

### *BUB1* is an essential gene for MPM cell survival

**Fig. 3:**
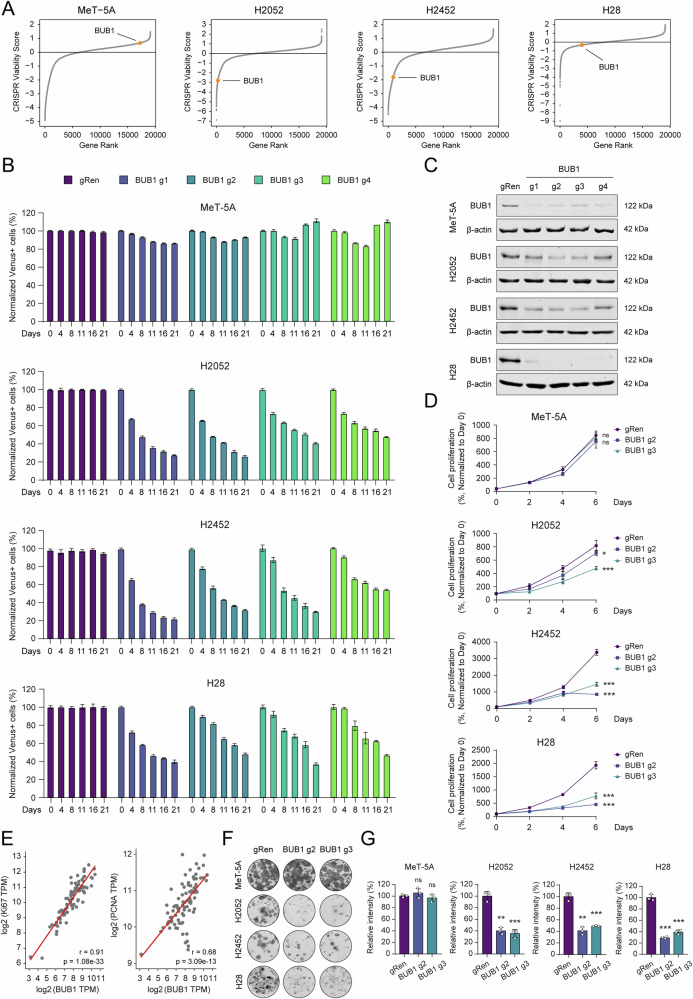
BUB1 is a critical regulator of MPM cell survival and proliferation. **A** Dot plots indicate the CRISPR viability scores of the *BUB1* gene in all four cell lines. **B** Competitive cell proliferation assay results of MeT-5A, H2052, H2452, and H28 cells infected with gRNAs targeting the *BUB1* gene. The percentage of Venus-positive cells at Day 0 was normalized to 100%, and the following measurements were calculated accordingly. Bar graphs are presented as the mean ± SD of three replicates. **C** Western blot assessment of *BUB1* knockout in MeT-5A, H2052, H2452, and H28 cells. Cells were infected with lentiCRISPR v2 gRen (control) or lentiCRISPR v2 BUB1 (g1, g2, g3, and g4) lentiviral vectors and selected with puromycin for 3 days. BUB1 levels were detected at 6 days post-infection. β-actin was used as the equal loading control. **D** The MTT assay measures the proliferation rates of BUB1-depleted MeT-5A, H2052, H2452, and H28 cells. Data are presented as the mean ± SD of six replicates. Two-tailed Student’s *t*-test was used for statistical analysis. *p < 0.05, ***p < 0.001, ns not significant. **E** Correlation analysis of expression levels between BUB1 and proliferation markers Ki67 and PCNA in the TCGA MPM dataset. Log2 transformed mRNA levels were compared through cBioPortal. Pearson correlation coefficient (*r*) for Ki67 is 0.91 (*p* value = 1.08e-33), and for PCNA, it is 0.68 (*p* value = 3.09e-13). **F** Representative images showing decreased 2D colony formation capacity of MeT-5A, H2052, H2452, and H28 cell lines upon BUB1 depletion. Colony formation assay was performed in triplicates in 12-well cell culture plates for 10–14 days. High-resolution images of the plates were acquired by LI-COR Odyssey CLx Imaging System. **G** Crystal violet intensity data showing the relative difference in 2D colony forming capacity of gRen and BUB1 gRNA expressing cells. Image Studio software was used to measure signal intensities. Bar graphs are presented as the mean ± SD of three replicates. Two-tailed Student’s *t*-test was used for statistical analysis. **p < 0.01, ***p < 0.001, ns not significant.

Among the uncharacterized high-confidence screening hits, we prioritized BUB1 for in-depth functional and translational interrogation. Despite exhibiting a relatively lower CRISPR viability score in H28 cells, BUB1 depletion imposed a pronounced and selective growth disadvantage in cancer cells compared to nonmalignant controls (Fig. 3A). Critically, BUB1 interaction partners and substrates annotated in the StringDB database, including *BUB3*, *CDC20*, *CDK1*, and *NDC80*, were also among the highly scoring genes. Intriguingly, however, these hits did not recapitulate the cancer-selective depletion phenotype (Fig. S4A).

*BUB1* encodes a serine-threonine kinase that phosphorylates core mitotic checkpoint proteins, orchestrating spindle assembly and chromosome segregation during mitosis [9]. Aberrant expression of BUB1 is associated with chromosomal misalignment, genomic instability, and aneuploidy [26], and its oncogenic role is increasingly recognized in human malignancies [27, 28]. Furthermore, BUB1 is considered a druggable target, supported by existing selective inhibitors or bioactive compounds [29, 30], and structural models from canSAR (https://cansar.ai) [31], that highlight ligandable cavities within the protein kinase domain (Fig. S4B). Nonetheless, the specific relevance of BUB1 in MPM pathogenesis and its potential as a therapeutic vulnerability remains elusive.

To bridge this knowledge gap, we dissected the BUB1 function in MPM, first assessing its impact on cellular survival. Using multiple gRNAs designed for BUB1 ablation, we performed competitive cell proliferation assays to compare growth dynamics between MPM cells and the MeT-5A cell line. Strikingly, BUB1 knockout selectively compromised the proliferation of MPM cells (Fig. 3B). In parallel, we generated BUB1-depleted clones using the same set of gRNAs, and selected two clones (g2 and g3) with the greatest overall knockout efficacy (Fig. 3C). Consistent with screening results, BUB1 loss reduced MPM cell proliferation, as measured by the cell viability assay (Fig. 3D). Additionally, analysis of BUB1 mRNA expression in the TCGA MPM dataset—as an indicator of tumor cell proliferation—revealed a strong positive correlation between BUB1 expression and well-established proliferation and cell cycle markers, Ki67, PCNA, CCNB1, and CDC20 (Figs. 3E and S4C). Long-term crystal violet assays further indicated a notable reduction in the 2D clonogenic capacity of BUB1-deleted cancer cells (Fig. 3F, G). Integration of Project Achilles data with CCLE RNA-seq profiles consistently acknowledged the essential nature and high expression of BUB1 across all MPM cell lines (Fig. S4D, E). Significantly, the introduction of a gRNA-resistant BUB1 cDNA constructs into endogenous BUB1-targeted cells fully rescued protein levels (Fig. S4F) and effectively counteracted the lethal phenotype caused by BUB1 genetic perturbation (Fig. S4G, H). These results collectively establish BUB1 as a key regulator of MPM cell survival and proliferation.

### BUB1 depletion impairs cancer cell growth and proliferation

**Fig. 4:**
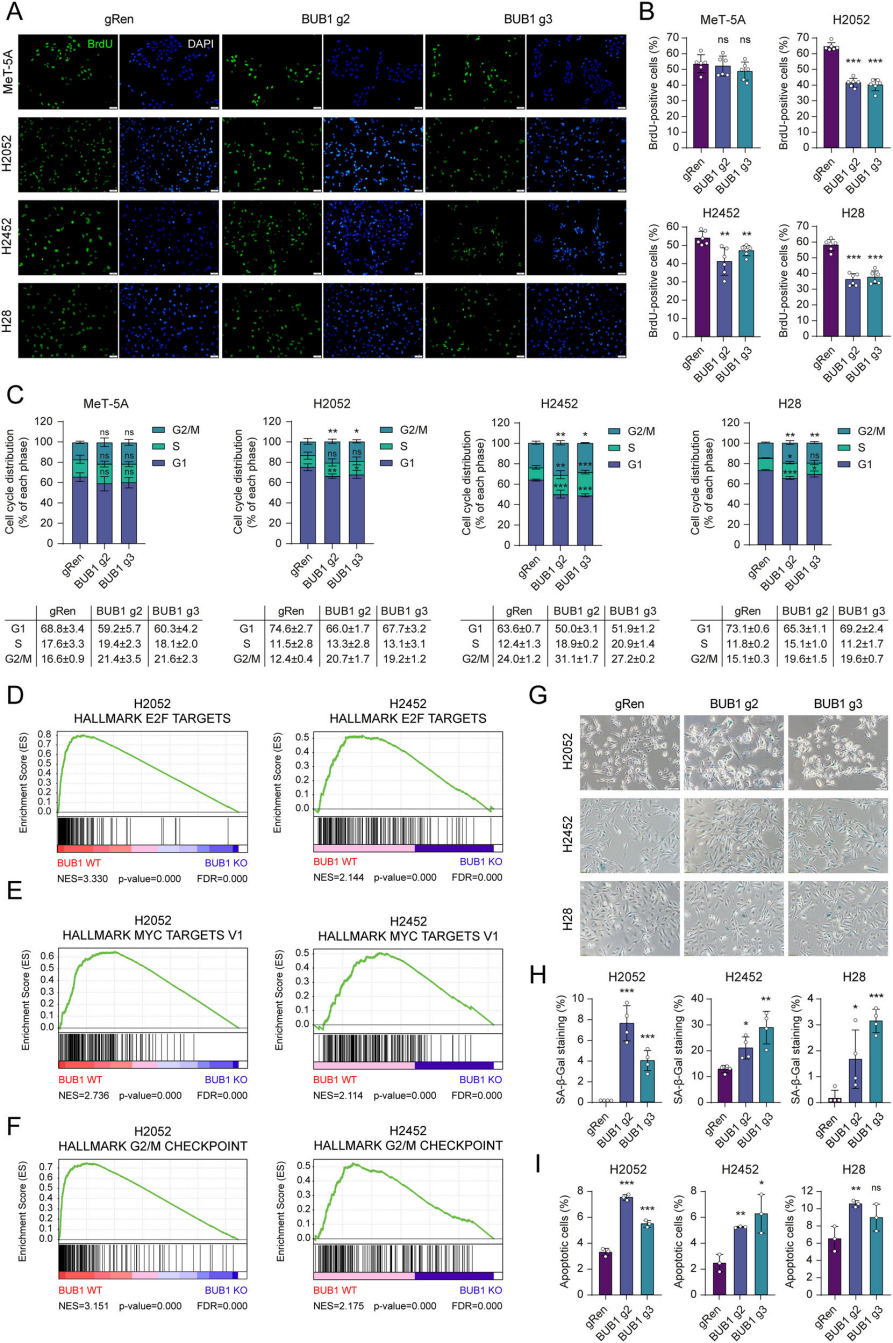
BUB1 depletion disrupts cell cycle progression and induces senescence and apoptosis in MPM cells. **A** Representative image of BrdU incorporation assay showing reduced cell proliferation index (BrdU positivity, green, 12 h labeling) of BUB1-depleted cells. DAPI was used as a nuclear counterstain (Blue). Scale bar: 50 μm. **B** BrdU-positive cell percentages are presented as the mean ± SD, *n* = 6. Two-tailed Student’s *t*-test was used for statistical analysis. **p < 0.01, ***p < 0.001, ns not significant. **C** Distribution of cell cycle phases of BUB1-depleted cells. Data are presented as mean ± SD, *n* = 3. Statistical analysis for multiple comparisons was performed by two-way ANOVA. *p < 0.05, **p < 0.01, ***p < 0.001, ns not significant. GSEA plots from H2052 and H2452 cell lines. The plots indicate significantly depleted E2F (**D**), MYC (**E**), and G2/M checkpoint (**F**) target gene expression, all reflecting repression of cell proliferation in MPM cell lines with *BUB1* knockout (*BUB1* KO). The *y*-axis represents the enrichment score (ES). The significance of correlation is depicted on the *x*-axis, by the red color for positive and the blue color for negative correlation. Normalized enrichment score (NES), false discovery rate (FDR), and *p* values are shown. **G** Representative images of SA-β-Gal staining (blue) assay identifying increased senescence in BUB1-depleted cells compared to gRen control cells. Scale bar: 100 µm. **H** Percentage of SA-β-Gal staining. All cell populations as well as SA-β-Gal stained cells were counted and the percentage of SA-β-Gal staining was calculated by proportioning the number of stained cells to the total number of cells. Data are presented as mean ± SD, *n* = 4. Two-tailed Student’s *t*-test was used for statistical analysis. *p < 0.05, **p < 0.01, ***p < 0.001. **I** Bar graphs of the Annexin V/PI apoptosis assay revealing increased apoptosis in BUB1-depleted cells compared to gRen control cells. The percentage of apoptotic cells was calculated as the sum of early and late apoptosis. Data are presented as mean ± SD, *n* = 3. Two-tailed Student’s *t*-test was used for statistical analysis. *p < 0.05, **p < 0.01, ***p < 0.001, ns not significant.

To gain a comprehensive understanding of how BUB1 depletion impacts cellular programs that govern proliferation and survival, we performed a series of assays. First, cells were labeled with 5′-bromo-2′-deoxyuridine (BrdU) to measure DNA synthesis during the S phase of the cell cycle [32]. Consistent with earlier reports in other malignancies [28, 33], BUB1 targeting triggered a significant decline in the rate of newly synthesized DNA in MPM cell lines (Fig. 4A, B). Given the established role of BUB1 in cell cycle machinery [34], we reasoned that the proliferative arrest might reflect aberrant cell cycle progression. Indeed, flow cytometry-based cell cycle profiling indicated a pronounced G2/M arrest with a concurrent accumulation in the S phase in BUB1-depleted cancer cells, particularly prominent in H2452 cells (Fig. 4C). To delineate the molecular underpinnings of these phenomena, we performed genome-wide RNA expression profiling. Principal component analysis and volcano plots revealed extensive transcriptomic reprogramming in BUB1 knockout (KO) cells (Fig. S5A, B). Differential expression analysis identified numerous genes modulated by BUB1 loss, with 1540, 1749, and 648 genes altered in H2052, H2452, and H28 cells, respectively (Fig. S5C). Importantly, gene set enrichment analysis (GSEA) highlighted significant depletion of E2F and MYC target gene signatures, as well as downregulation of G2/M checkpoint pathways (Figs. 4D–F and S5D, E). Parallel interrogation of independent MPM patient cohorts (GSE2549 [35], GSE29211 [36], GSE42977 [37], and GSE163722 [38]) corroborated the enrichment of proliferation and cell cycle-related gene sets in tumors with elevated BUB1 levels (Fig. S6A–C), implying that high BUB1 expression promotes tumor cell proliferation in vivo. Furthermore, existing literature links lower BUB1 levels to cellular senescence, a process dependent on intact p53 pathway [39]. Consistent with the knowledge that our MPM cell lines harbor wildtype p53, transcriptomic landscapes in BUB1-targeted cells indicated an enrichment of p53- and senescence-related gene signatures (Fig. S5D). At the cellular level, BUB1 depletion led to an accumulation of SA-β-Gal–positive cells (Fig. 4G, H), with more significant effects in single-cell clones (Fig. S5F). However, the extent of senescence induction was modest, suggesting that this phenotype is only one facet of the antiproliferative program unleashed by BUB1 loss. Concomitantly, we observed activation of TNF/NF-kB and IL6/JAK/STAT3 signaling in BUB1-deficient cells (Fig. S5D), with corresponding changes in target gene expression (Fig. S5G). Consistent with studies implicating BUB1 in the regulation of other signaling networks [40, 41], we also noted alterations in TGF-β and MTORC1 pathways, specifically in H2052 cells (Fig. S5D). Lastly, we investigated whether BUB1 depletion would induce programmed cell death. With the exception of a mild, non-significant increase in H28 *BUB1* g3 cells, other clones showed a modest but significant induction in apoptotic rates (Fig. 4I), confirmed by gene set enrichment in genes mediating apoptosis by caspase activation (Fig. S5D). Collectively, our cellular and transcriptomic findings position BUB1 as a critical node in MPM pathogenesis, linking its depletion to G2/M cell cycle arrest, partial senescence, and apoptosis.

### BUB1 knockout attenuates aggressive cancer cell traits

**Fig. 5:**
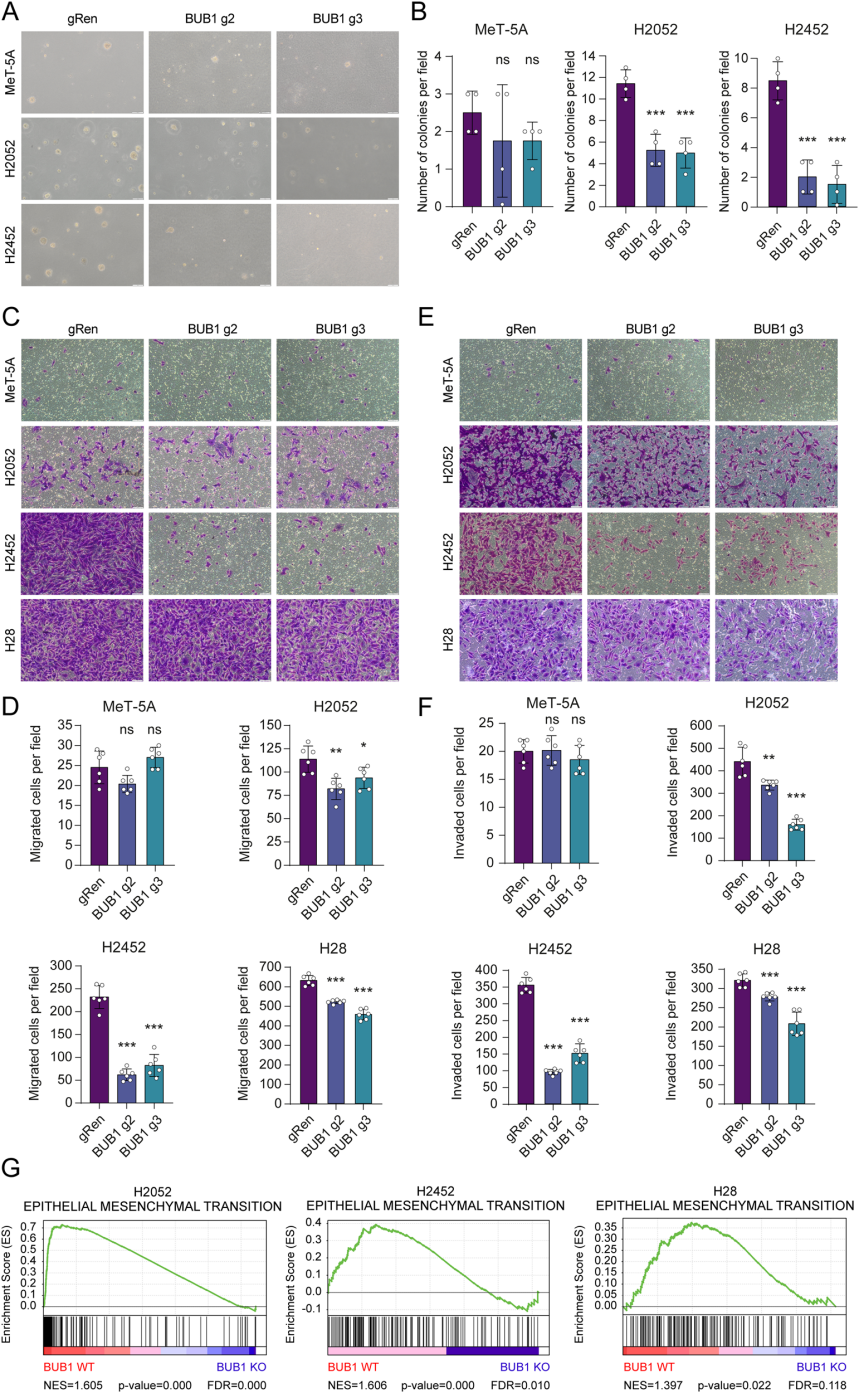
Impact of BUB1 knockout on aggressive cell fates and EMT signature. **A** Representative image of soft agar colony formation assay. Scale bar: 100 µm. **B** Bar graphs showing the number of colonies with a diameter greater than 35 µm. Data are presented as mean ± SD, *n* = 4. Two-tailed Student’s *t*-test was used for statistical analysis. ***p < 0.001, ns not significant. Representative images of transwell migration (**C**) and invasion (**E**) assays in BUB1-depleted cells, with their relative controls. Scale bar: 100 μm. Migrated (**D**) and invaded (**F**) number of cells per field. ImageJ software was used for manual cell counting. Data are presented as the mean ± SD, *n* = 6. Two-tailed Student’s *t*-test was used for statistical analysis. *p < 0.05, **p < 0.01, ***p < 0.001, ns not significant. **G** Representative GSEA plots from H2052, H2452, and H28 cell lines. The plots show significantly depleted EMT target genes, reflecting decreased cell motility in MPM cells with BUB1 knockout (BUB1 KO). The *y*-axis represents the enrichment score (ES). The degree of correlation is represented on the *x*-axis, by the red color for positive and the blue color for negative correlation. Normalized enrichment score (NES), false discovery rate (FDR), and *p* values are shown.

Beyond sustaining proliferation, many oncogenes reinforce functional hallmarks of malignancy [42]. We explored whether BUB1 influences anchorage-independent growth, motility, and invasiveness. Remarkably, soft agar assays revealed a substantial decline in the anchorage-independent clonogenic capacity of BUB1-depleted H2052 and H2452 cells, while MeT-5A cells, which already exhibited very low clonogenic potential, remained unaffected (Fig. 5A, B). H28 cells were incapable of colony formation in soft agar, precluding their evaluation. Our subsequent objective was to examine the role of BUB1 on the invasive nature of MPM cells through transwell assays. Conclusively, the absence of BUB1 significantly reduced both migration (Fig. 5C, D) and invasion (Fig. 5E, F) in MPM cells. In contrast, MeT-5A cells were inherently characterized by low invasive capacity with no significant alterations by BUB1 ablation. Thus, BUB1 is indispensable for sustaining 3D growth and aggressive phenotypes of MPM cells.

Considering the landscape of molecular mechanisms governing MPM tumor invasiveness and their prognostic implications, particularly the epithelial-to-mesenchymal transition (EMT) [43, 44], we interrogated the potential nexus between BUB1 expression and EMT-associated traits. Consistent with the attenuated motility and invasiveness, GSEA indicated significant downregulation of hallmark EMT signatures in BUB1-deleted cells (Fig. 5G). Supporting this conjecture, the GSEA of publicly available datasets (GSE2549, GSE29211, and GSE163722) confirmed a positive correlation between high BUB1 levels and EMT phenotypes in MPM tumors (Fig. S7).

**Fig. 6:**
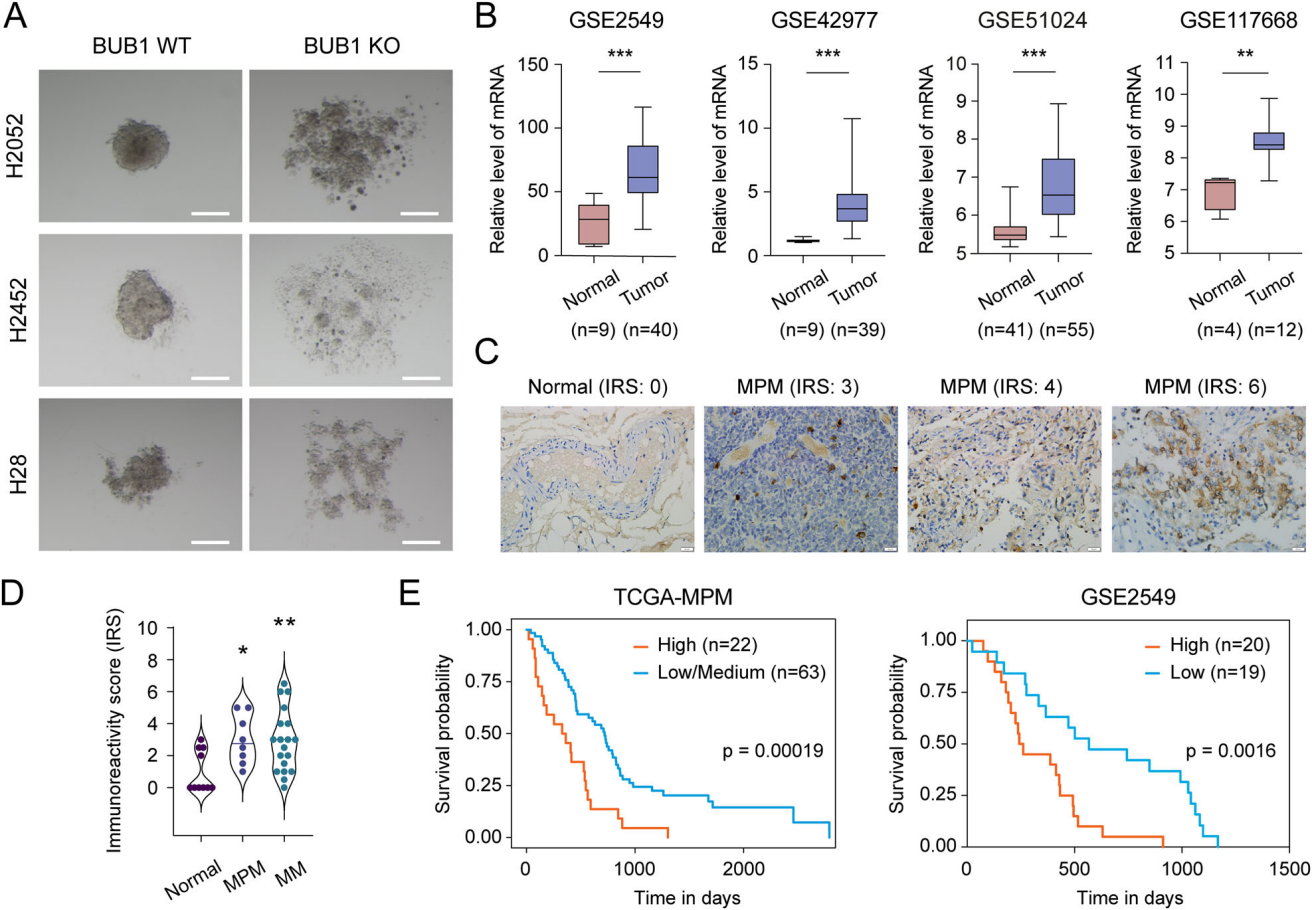
BUB1 depletion disrupts spheroid growth and highlights prognostic significance in MPM. **A** Representative image of hanging drop assay. BUB1 depletion impaired the spheroid formation capacity of MPM cells. Spheroids (*n* = 13, for each condition) were imaged using a stereo microscope. Scale bar: 200 μm. **B** The Box–Whisker plot of BUB1 mRNA expression levels in GSE2549, GSE42977, GSE51024, and GSE117668 datasets. **C** Representative IHC images depicting different immunoreactivity scores (IRS, ranging from 0 to 6) of BUB1 staining in MPM tissues. IRS was calculated by the multiplication of proportion of positive cells and the intensity of staining. Scale bar: 20 μm. **D** IHC-based analysis of BUB1 protein expression in human MPM tumor tissues (*n* = 10) compared to noncancerous normal samples (*n* = 10). MM: mesothelioma of other tissues. Two-tailed Student’s *t*-test was used for statistical analysis. *p < 0.05, **p < 0.01. **E** The Kaplan–Meier survival plots of BUB1 in the TCGA MPM and GSE2549 datasets compare high gene expression (orange) relative to the low/medium or low patients (blue). *p* values are shown on the plots.

To further explore BUB1’s contribution to tumorigenic properties, we examined spheroid formation in a hanging drop culture system [45]. Unlike control groups, cells deficient for BUB1 failed to coalesce into cohesive 3D spheroid structures (Fig. 6A). From a clinical perspective, integrative analysis of multiple datasets, comparing tumor-adjacent normal vs MPM tissues (GSE2549, GSE42977, and GSE51024 [46]) as well as normal stromal vs MPM cell lines (GSE117668), consistently showed that high BUB1 expression characterizes aggressive malignant phenotypes (Fig. 6B). To strengthen these findings, we then conducted IHC profiling of BUB1 expression in a human tissue microarray (TMA) with 10 MPM cases, 17 malignant mesothelioma of other tissues, and 10 normal tissues. The analysis revealed heterogeneous immunoreactivity scores (IRS, ranging from 0 to 6) associated with both nuclear and cytoplasmic staining patterns (Figs. 6C and S8). Specifically, MPM tissues scored for mild (IRS: 2–3) to moderate (IRS: 4–6) staining. In contrast, normal mesothelium specimens were predominantly negative (IRS: 0 in 6 out of 10 cases). Thus, BUB1 levels were elevated in MPM tumors and other mesothelioma tissues (MM) (Fig. 6C, D). Consistently, Kaplan–Meier survival analyses across different genomic platforms (i.e., TCGA MPM cohort and GSE2549 dataset) linked higher BUB1 expression with shorter overall survival (Fig. 6E). Taken together, these findings reinforce BUB1’s role as an oncogenic driver in MPM progression.

### Ectopic BUB1 expression enhances cancer cell proliferation and aggressive traits

**Fig. 7:**
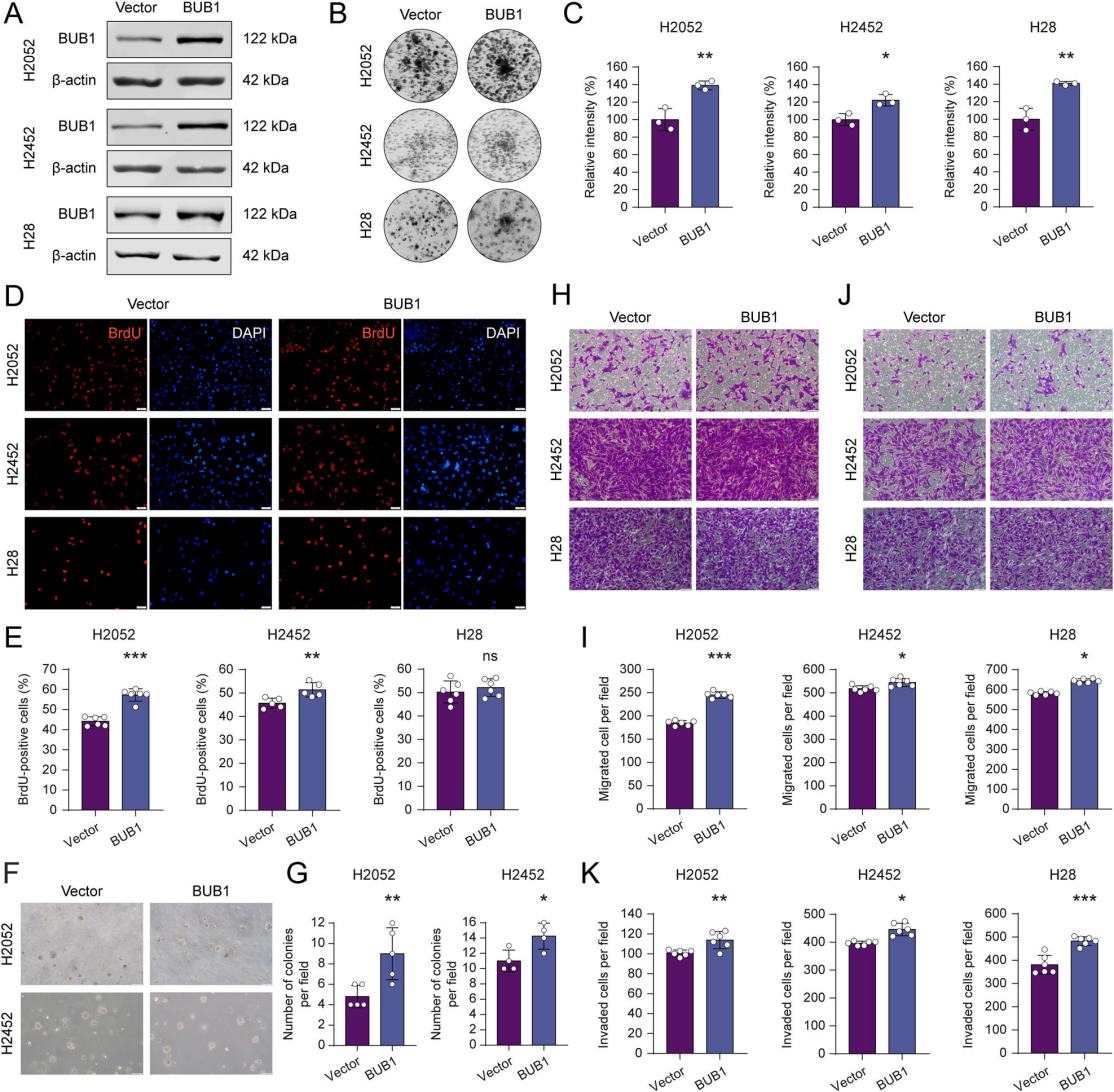
BUB1 overexpression amplifies proliferative and malignant phenotypes. **A** BUB1 protein levels in empty vector (Vector) and BUB1 overexpression vector (BUB1) transduced H2052, H2452, and H28 cells. β-actin was used as a loading control. **B** Representative images showing increased 2D colony formation capacity of H2052, H2452, and H28 cell lines upon BUB1 overexpression. Colony formation assay was performed in triplicates in 12-well cell culture plates for 10–14 days. High-resolution images of the plates were acquired by LI-COR Odyssey CLx Imaging System. **C** Crystal violet intensity data showing the relative difference in 2D colony forming capacity of Vector and BUB1-overexpressing cells. Image Studio software was used to measure signal intensities. Bar graphs are presented as the mean ± SD of three replicates. Two-tailed Student’s *t*-test was used for statistical analysis. *p < 0.05 and **p < 0.01. **D** Representative images of BrdU incorporation assay identifying increased cell proliferation index (BrdU positivity, red, 12 h incubation) in BUB1-overexpressing H2052, H2452, and H28 cells compared to Vector control cells. DAPI was used as the nuclear counterstain (Blue). Scale bar: 100 μm. **E** BrdU-positive cell percentages are presented as the mean ± SD, *n* = 6, *n* = 5 for H2452. Two-tailed Student’s *t*-test was used for statistical analysis. **p < 0.01 and ***p < 0.001, ns not significant. **F** Representative images of soft agar colony formation assay. Scale bar: 100 µm. **G** Bar graphs showing the number of colonies with a diameter greater than 35 µm. Data are presented as mean ± SD, *n* = 5 for H2052, *n* = 4 for H2452. Two-tailed Student’s *t*-test was used for statistical analysis. *p < 0.05 and **p < 0.01. Representative images of transwell migration (**H**) and invasion (**J**) assays upon BUB1 overexpression with their relative controls. Scale bar: 100 μm. Migrated (**I**) and invaded (**K**) number of cells per field. ImageJ software was used for manual cell counting. Data are presented as the mean ± SD, *n* = 6. Two-tailed Student’s *t*-test was used for statistical analysis. *p < 0.05, **p < 0.01, ***p < 0.001.

Encouraged by the rescue experiments (Fig. S4F–H), we investigated whether ectopic expression of BUB1 further augments oncogenic phenotypes. Indeed, stable overexpression of BUB1 (Fig. 7A) enhanced 2D clonogenic cell growth (Fig. 7B, C) and modestly increased the overall rate of active DNA synthesis, except in the H28 cell line which did not demonstrate a similar enhancement (Fig. 7D, E). Akin to the effects on cell proliferation, forced BUB1 expression also enhanced 3D anchorage-independent growth (Fig. 7F, G). Finally, transwell assays revealed a moderate but significant increase in the migration (Fig. 7H, I) and invasion (Fig. 7J, K) abilities of BUB1-overexpressing cells. The cumulative evidence from these studies accentuates BUB1’s capacity to fuel malignant phenotypes in MPM cells, strengthening its value as a therapeutic target.

### BUB1 is indispensable for spindle assembly checkpoint function and cytokinesis

**Fig. 8:**
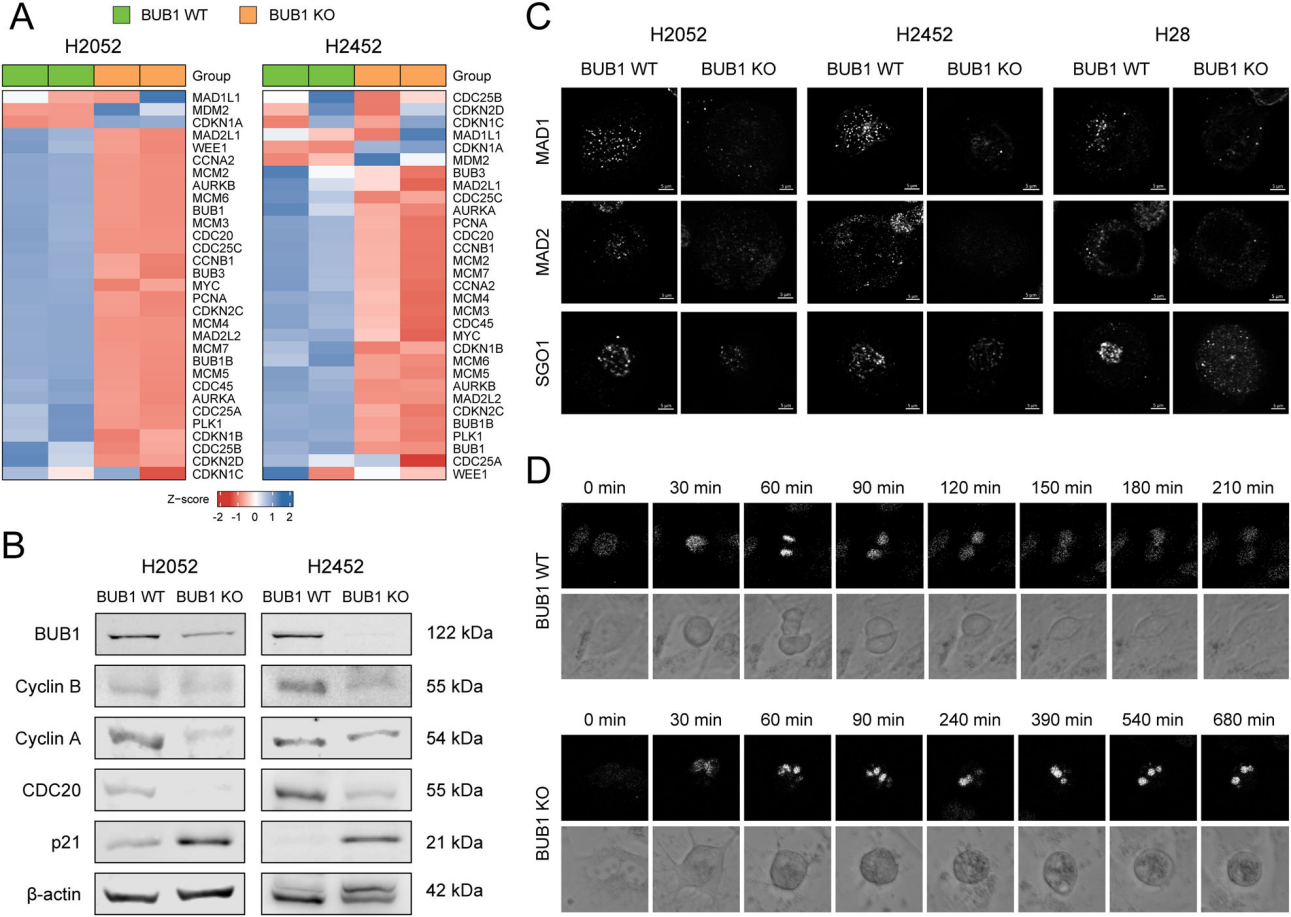
BUB1 is indispensable for maintaining SAC integrity and cytokinesis. **A** Heatmap depicting the expression levels of BUB1 local network genes (highlighted in Fig. S9A) in BUB1 knockout (BUB1 KO) H2052 and H2452 cells. **B** BUB1, Cyclin B, Cyclin A, CDC20 and p21 protein levels in BUB1 WT vs BUB1 KO cells. β-actin was used as a loading control. **C** Localization of MAD1, MAD2, and SGO1 in mitotic MPM cells demonstrated through immunofluorescence staining. Cells were synchronized with a 12 h thymidine block followed by a 12 h release in a complete culture medium or 150 mM nocodazole for H28 cells. The images were captured as z-stacks using Apotome 3 (Zeiss) and subsequently processed into stacked projections. **D** Time-lapse live cell microscopy illustrating mitotic progression of BUB1 knockout H2452 cells compared to control. Cells were stained with Hoechst and monitored using confocal microscopy by acquiring z-stacks every 10 min for 20 h. Representative image sequences were aligned on the time axis and presented in the figures.

As a keystone regulator of the spindle assembly checkpoint (SAC), BUB1 ensures accurate chromosome alignment and segregation by delaying anaphase onset until all kinetochores engage with spindle microtubules [47]. It recruits and activates MAD1 (Mitotic Arrest Deficient 1) and MAD2 (Mitotic Arrest Deficient 2), forming a complex that inhibits the anaphase-promoting complex (APC/C) [48], and cooperates with Shugoshin-1 (SGO1) to safeguard centromeric cohesion [49]. BUB1 also interacts with CDC20 (Cell Division Cycle 20) and other mitotic checkpoint complex (MCC) components, framing a tightly regulated network that governs mitotic progression [50]. Intriguingly, transcriptomic landscape of BUB1 local network genes (Fig. S9A), indicated global dysregulation of SAC, MCC, and cell cycle machinery, particularly in H2052 and H2452 cells (Figs. 8A and S9B). This includeda pronounced decrease in the expression levels of CDC20, Cyclin A (encoded by *CCNA2*), and Cyclin B (encoded by *CCNB1*), coupled with a reciprocal increase in the cyclin-dependent kinase inhibitor p21 (encoded by *CDKN1A*) (Figs. 8A, B and S9B, C). Fluorescence imaging of genetically ablated cells confirmed the defective localization of MAD1, MAD2, and SGO1 (Fig. 8C), suggesting diminished SAC efficiency and compromised centromeric cohesion. Long-term time-lapse imaging of Hoechst-stained nuclei during mitosis further revealed multinucleation in the absence of BUB1 (Fig. 8D), consistent with a failure to complete cytokinesis. These changes indicate a G2/M cell cycle arrest or prolonged mitosis due to impaired mitotic fidelity, emphasizing BUB1’s indispensable function in maintaining SAC integrity and cytokinesis in MPM cells.

### Pharmacological inhibition of BUB1 inhibits cancer cell phenotypes

**Fig. 9:**
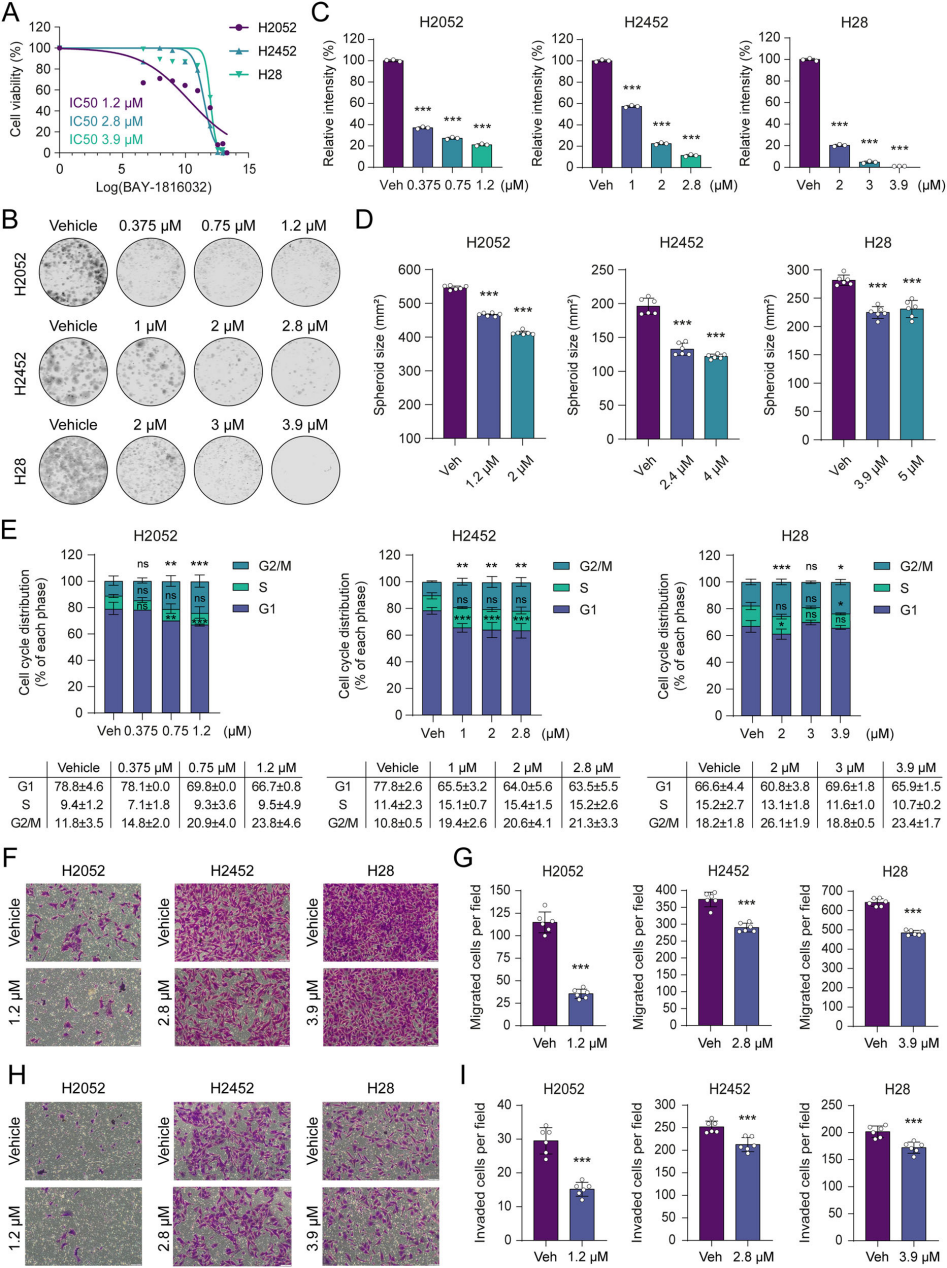
Pharmacological inhibition of BUB1 kinase attenuates MPM cell phenotypes. **A** MTT assay measuring the IC50 values of H2052, H2452, and H28 cells. Cells were treated with increasing doses of BAY-1816032 ranging from 0.25 to 10 μM. Control cells were treated with DMSO. The experiment was performed in 2 biological and 6 technical replicates. **B** Representative images showing 2D colony formation capacity of H2052, H2452, and H28 cell lines upon BAY-1816032 treatment. Experiments were performed in triplicates in 12-well plates. Cells were treated for 3 days with IC50 and two doses under IC50 of BAY-1816032 and cultured for another 7–11 days. High-resolution images were captured with the LI-COR Odyssey CLx Imaging System. **C** Crystal violet intensity data showing the relative difference in 2D colony forming capacity of Vehicle (DMSO) and BAY-1816032 treated cells. Image Studio software was used to measure signal intensities. Bar graphs are presented as the mean ± SD of three replicates. Two-tailed Student’s *t*-test was used for statistical analysis. ***p < 0.001. **D** Bar graphs showing the decrease in size of spheroids upon pharmacological BUB1 inhibition. Data are presented as mean ± SD, *n* = 6. Two-tailed Student’s t-test was used for statistical analysis. ***p < 0.001. **E** Distribution of cell cycle phases of BAY-1816032 treated H2052, H2452, and H28 cells. Data are presented as mean ± SD, *n* = 3. Statistical analysis was performed by two-way ANOVA multiple comparison. *p < 0.05, **p < 0.01, ***p < 0.001, ns not significant. Representative images of transwell migration (**F**) and invasion (**H**) assays of BAY-1816032 treated cells, with their relative controls. Scale bar: 100 μm. Migrated (**G**) and invaded (**I**) number of cells per field. ImageJ software was used for manual cell counting. Data are presented as the mean ± SD, *n* = 6. Two-tailed Student’s *t*-test was used for statistical analysis. ***p < 0.001.

To recapitulate genetic loss-of-function findings and interrogate the druggability of BUB1, we conducted pharmacological inhibition experiments with BAY-1816032, a potent and highly selective small-molecule inhibitor designed to target its kinase activity [29] (Fig. S4B). Drug treatment studies identified low micromolar IC50 values for H2052, H2452, and H28 cell lines through viability assays, calculated as 1.2, 2.8, and 3.9 µM, respectively (Fig. 9A). Moreover, BAY-1816032 treatment abrogated 2D colony formation in a dose-dependent manner (Fig. 9B, C), and reduced tumor spheroid growth on ultra-low adhesion surfaces (Figs. 9D and S10A). Mechanistically, BUB1 inhibition induced cell cycle arrest in the G2/M phase in a dose-dependent manner in H2052 and H2452 cells (Fig. 9E), phenocopying the cell cycle perturbations observed upon genetic ablation. Although apoptosis induction was notable yet statistically insignificant (Fig. S10B), the inhibitor dramatically attenuated MPM cell migration (Fig. 9F, G) and invasion (Fig. 9H, I). These novel findings suggest that MPM tumor-promoting activities of BUB1 depend, at least in part, on its kinase function and provide support for the druggability of this kinase in MPM.

## Discussion

MPM poses a formidable clinical challenge, with current treatment modalities offering limited efficacy for achieving prolonged survival [51]. Unlike certain other cancer types [52], MPM has an evident dearth of well-defined landscapes of actionable molecular targets. Recent advances in genetics and functional genomics, particularly genome-wide or pooled loss-of-function CRISPR screens, have proven effective for unveiling context-dependent vulnerabilities and high-confidence therapeutic targets in oncology [53].

Our primary objective was to identify MPM-relevant vulnerabilities with direct translational potential. Harnessing the Brunello knockout library, we conducted systematic and cross-comparative CRISPR screens in three MPM cell lines and one nonmalignant mesothelial line. Pathway and biological process enrichment analyses, supported by correlation with DepMap datasets [14], accentuated the robust performance of our CRISPR screening platform. Leveraging the MAGeCK algorithm and CRISPR viability scores, we focused on consistent depletions in tumor cells that displayed minimal or absent effects in the mesothelial cell line. This approach unveiled cancer cell-selective hits such as BUB1, CDK2, and VPS37A. Notably, CDK2, a kinase central to G1/S control and G2-phase progression by ensuring proper DNA replication and entry into mitosis [20], emerged as a druggable candidate, with small-molecule inhibitors like Roscovitine/Seliciclib and Dinaciclib showing antitumor activity in preclinical and clinical settings [54]. Arguably, targeted inhibition of CDK2 could synergize with therapies exploiting checkpoint dependencies in the setting of MPM. Similarly, VPS37A, an ESCRT component linked to autophagy and membrane trafficking, has been implicated in tumor progression [55, 56]. While direct pharmacologic targeting remains to be interrogated, VPS37A showed potential for translational exploration. Most notably, both genes correlated inversely with patient survival, underscoring their prognostic and therapeutic relevance for MPM. In addition to probing tumor-specific vulnerabilities, our screens identified AURKA, a prominent oncology target undergoing clinical evaluation [57], as a recurrent hit across all cell lines. Although AURKA emerged as indispensable in nonmalignant cells, this aligns with the concept that certain hits with a high therapeutic index [58] may also manifest as human pan-essential genes. This would postulate that our screen data has the potential to map additional recurrent yet high-confidence druggable hits. Supporting this, recent work in MSTO-211H cells proposed AURKA as a downstream target of KAP1, a chromatin reader identified as a non-genetic dependency in MPM [59].

The prioritization of BUB1 as a high-confidence differential gene is justified by its druggable nature, mechanistic plausibility, canonical function in spindle assembly control and chromosome alignment and segregation [9], and potential clinical tractability. In MPM, where stringent cell cycle control is paramount for maintaining genome integrity [60], BUB’s primary function in mitotic machinery offers further rationale for functional dissection and translational investigation. The significance of BUB1 as a key driver in cancer progression is an evolving field of interest. Elevated BUB1 expression correlates with aggressive phenotypes and unfavorable prognosis in various cancers, such as pancreatic ductal adenocarcinoma, breast cancer, glioblastoma, and papillary thyroid cancer [61–64]. Additionally, dysregulation of the BUB1 network has been identified as a central hub in the genomic landscape of lung adenocarcinoma [65]. In transgenic models, BUB1 overexpression promotes chromosomal missegregation and aneuploidy, leading to an increased predisposition to spontaneous cancer [27].

Functional studies in MPM cells confirmed that BUB1 depletion disrupts 2D colony formation and 3D anchorage-independent growth, impairs DNA synthesis, and provokes G2/M arrest. Consistent with its critical role in sustaining kinetochore integrity and SAC signaling [66], BUB1 loss attenuated SAC activity and precipitated multinucleation, likely stemming from defective cytokinesis, which can instigate tumor-suppressive mechanisms via p53 signaling [67]. At the molecular level, BUB1 ablation resulted in extensive gene expression alterations within its regulatory network, including downregulation of CDC20, cyclin A, and cyclin B, coupled with upregulation of p21 [68, 69]. These changes highlight the central and multifaceted role of BUB1 in maintaining mitotic fidelity and controlling cell cycle dynamics.

Clinically, the positive correlation between BUB1 expression and E2F/MYC transcriptional targets, along with the G2/M checkpoint components, positions BUB1 as a potential marker of proliferative capacity in MPM tumors. Beyond these insights, BUB1 loss impaired migration and invasion, consistent with reports linking BUB1 to TGF-β signaling, cancer stemness, and EMT axis in lung and breast cancer cells [40, 62, 70]. Crucially, the demonstration that BUB1 is essential for aggressive MPM tumor cell fates, combined with its elevated expression predicting poor patient prognosis, strengthen the rationale for its therapeutic exploitation.

Preclinical evidence supports the druggability of BUB1 as a kinase. Recent studies have identified BAY-1816032, a highly selective compound with low micromolar antiproliferative activity in many cell lines, as a suitable tool for proof of principle studies [29]. Importantly, combining BUB1 inhibition with additional therapeutic agents enhances antitumor efficacy. For example, blocking BUB1 kinase activity can effectively reverse taxane resistance in castration-resistant prostate cancer [71]. Similarly, dual targeting of BUB1 with ATR or PARP inhibitors yields synergistic activity against triple-negative breast cancer cells [29]. In our study, pharmacological BUB1 inhibition at single-digit micromolar concentrations attenuated 2D and 3D survival of MPM cells, induced G2/M cell cycle arrest, and impaired functional traits of malignant cells.

Our findings establish a strong foundation for the translational pertinence of BUB1 inhibition in MPM, advocating for its optimal testing in clinically relevant models. The mechanistic underpinnings through which BUB1 inhibition provokes anti-oncogenic responses involve transcriptomic reprogramming and molecular outcomes that perturb SAC function and cytokinesis fidelity. Synthesizing this information with CRISPR viability scores of BUB1 interaction partners, substrates, and other mitotic kinases, it is plausible to speculate that the G2/M checkpoint regulation constitutes a unique vulnerability in MPM cells. This hypothesis is reinforced by a recent report demonstrating that KAP1-mediated modulation of G2/M-specific gene programs is indispensable for the correct execution of mitosis and MPM cell survival [59]. Accordingly, selective targeting of mitotic regulatory networks may represent an effective therapeutic strategy. Most critically, our findings highlight the potency of unbiased comparative CRISPR screening in exposing cancer-cell-specific vulnerabilities, further proposing that focused libraries can be deployed to unmask additional exploitable liabilities within the mitotic G2/M checkpoint machinery.

## Methods

### Cell lines and culture conditions

Nonmalignant mesothelial cell line MeT-5A (CRL-9444) and MPM cell lines H2052 (NCI-H2052; CRL-5915), H2452 (NCI-H2452; CRL-5946), and H28 (NCI-H28; CRL-5820) were purchased from American Type Culture Collection (ATCC; Manassas, VA). All cell lines were cultured in RPMI-1640 medium (21875034, Gibco) supplemented with 10% fetal bovine serum (FBS; 10500064, Gibco) and penicillin-streptomycin (100 U/mL; 15140122, Gibco). HEK293T cell line, a kind gift from Dr. Nuri Ozturk (Gebze Technical University, Kocaeli, Türkiye), was cultured in high glucose Dulbecco’s Modified Eagle’s Medium (DMEM; 41965039, Gibco) supplemented with 10% FBS and penicillin-streptomycin (100 U/mL). All cell lines were maintained at 37 °C in a humidified incubator containing 5% CO_2_. Routine mycoplasma testing was conducted using a Mycoplasma PCR Detection Kit (G238, ABM), assuring the absence of contamination in all cell lines. Cryo-stock vials were made and low-passage cultures were used for all experiments. The selective BUB1 kinase inhibitor, BAY-1816032 (HY-103020, MedChemExpress), was reconstituted in DMSO (472301, Sigma Aldrich) under sterile conditions, aliquoted, and stored at −80 °C until used. Repeated freeze and thaw cycles were avoided.

### Lentiviral packaging and cell transduction

HEK293T cells were seeded in 60 mm plates to achieve ~80% confluency within 24 h. The cells were co-transfected with a target vector and two helper plasmids, psPAX2 (Addgene plasmid #12260) and pMD2.G (Addgene plasmid #12259), at a ratio of 4:2:1, using a total DNA amount of 6 µg. Polyethylenimine (PEI; 23966, Polysciences) was used as the transfection reagent at a 1:5 DNA-to-PEI ratio. Cell supernatants were collected 48 h after transfection and filtered through a 0.45 μm SFCA filter (431220, Corning). Target cells were seeded at ~50–60% confluency. The medium containing viral particles was diluted in complete growth media supplemented with polybrene (H9268, Sigma Aldrich) at a final concentration of 8 µg/mL and added to cells. After transduction for 48 h, infected cells were selected by puromycin treatment (ant-pr-1, Invivogen) or fluorescence-associated cell sorting (FACS) by FACSAria III (BD Biosciences), depending on the selection marker of the target plasmid.

### Generation of monoclonal cell lines with stable Cas9 expression

Monoclonal cell lines expressing stable Cas9 were established by lentiviral transduction of the lentiCas9-EGFP vector (Addgene plasmid #63592). The pool of GFP-positive cells was enriched via FACS and manually seeded into 96-well plates at a ratio of 0.5 cells per well. The wells were assessed for single-cell cloning, and the clones were allowed to proliferate in conditioned media collected from each parental cell line. The media were supplemented with 20% FBS to enhance colony growth. Single-cell clones were further expanded by transferring into larger growth cultures, resulting in the generation of 11–12 clonal sublines for each cell line. Cas9 protein expression in sublines was verified by western blotting. The genome editing performance of Cas9 cleavage was validated by the T7 Endonuclease I (T7EI) and competitive cell proliferation assays.

### gRNA cloning

Sequences compatible with restriction sites were appended at the 5′ and 3′ regions of forward and reverse primer sequences of designed gRNAs, and oligonucleotides were synthesized by Macrogen (South Korea). Oligonucleotide pairs were annealed, phosphorylated, and ligated into CRISPR-targeting plasmids, digested, and dephosphorylated with FastDigest BsmBI (Esp3I; FD0454, Thermo Scientific) and FastAP Thermosensitive Alkaline Phosphatase (EF0651, Thermo Scientific), respectively, as described in a previously published protocol [72]. Ligation products were transformed into Stbl3 *E. coli*-competent cells (Invitrogen). Single colonies grown overnight on LB agar plates with ampicillin selection were initially validated through colony PCR. Successful clones for gRNA insertion were further amplified by plasmid miniprep (740588, Macherey Nagel) or midiprep (740412, Macherey Nagel), and sequence-verified by Sanger sequencing.

### T7 Endonuclease I (T7EI) assay

To demonstrate genome targeting efficiency, clonal cell lines with Cas9 expression were infected for 48 h with lentiGuide-Puro vector (Addgene plasmid #52963) containing gRNAs targeting the Renilla luciferase gene (*Ren*, non-targeting control) and the *EGFR* gene [25]. Transduced cells were selected with puromycin for 72 h. Oligonucleotide sequences used in gRNA cloning are listed in Supplementary Table 1. Genomic DNA (gDNA) was salt-extracted from puromycin-selected cells and the *EGFR* gene region was amplified by PCR using the primer pairs listed in Supplementary Table 2. PCR amplicons were separated by agarose gel electrophoresis and gel-extracted using the NucleoSpin Gel and PCR Clean‑up kit (740609, Macherey Nagel). T7EI assay was conducted according to the manufacturer’s instructions. Briefly, 200 ng of purified PCR amplicons were incubated with 10X NEB Buffer and digested with 0.5 µL T7EI (M0302S, NEB) at 37 °C for 30 min. Fragmented PCR products were visualized on 2% agarose gel.

### Competitive cell proliferation assay

To assess the functional activity of Cas9 in clonal sublines, we employed a competitive cell proliferation assay, as previously described [25]. In brief, cells were partially infected with lentiGuide-Hygro-dTomato (Addgene plasmid #99376) lentiviral particles expressing gRNAs against the non-targeting control (Ren) and a cell essential gene (*RPA3*; Replication Protein A3), using oligonucleotide sequences listed in Supplementary Table 1 for RPA3 gRNA cloning. The percentage of dTomato-positive cells was tracked by flow cytometry every 4 days for 12 days, starting on Day 3 post-infection (referred to as Day 0). The percentage of tomato-positive cells at Day 0 was normalized to 100%, and the following measurements were calculated accordingly.

For validation studies, *AURKA*, *CDK2*, and *VPS37A* genes were targeted using gRNA sequences from the Brunello gRNA library. *BUB1* targeting gRNA sequences were retrieved from the Brunello, GeCKO, and Sabatini gRNA libraries. Oligonucleotide sequences used in gRNA cloning are listed in Supplementary Table 1. The gRNAs were inserted into the pECPV plasmid (EFS-Cas9-P2A-Venus), derived from the lentiCRISPR v2 backbone (Addgene plasmid #52961) by replacing the puromycin selection cassette with the Venus coding sequence [25, 73]. MeT-5A, H2052, H2452, and H28 parental cell lines were partially infected, and Venus-positive cells were monitored over time through flow cytometry (LSRFortessa, BD), starting on Day 0 (post-infection Day 5) and continuing with measurements at Days 4, 8, 11, 16, and 21. The percentage of Venus-positive cells at day 0 was set as 100%, and subsequent measurements were normalized accordingly.

### Brunello library amplification and lentivirus packaging

Human CRISPR Knockout Pooled Library (Brunello) in lentiGuide-puro backbone was purchased from Addgene (plasmid #73178). The Brunello library contains 76,441 unique gRNAs designed to target 19,114 genes in the human genome. It also includes a collection of 1000 negative control gRNAs, for a total of 77,441 gRNAs [74]. The library was amplified according to the supplier’s protocol. Specifically, 400 ng of the library was mixed with 100 µL of Endura ElectroCompetent Cells (60242-1, Lucigen). The plasmid-bacteria mixture was transferred into four pre-chilled electroporation cuvettes with a 0.1 cm gap, and electroporation was performed using a BioRad MicroPulser electroporator set to EC1 (6 ms, 1.8 kV). The library-bacteria mixtures were promptly collected into 13 mL tubes with 1 mL of pre-warmed recovery medium, and the total volume was adjusted to 10 mL. Subsequently, the culture was incubated at 225 rpm and 37 °C for 1 h. Transformed bacteria were divided into four groups as 2.5 mL and spread onto four bioassay plates (240835, Thermo Fisher) containing 100 µg/mL ampicillin. The plates were then incubated at 32 °C for 18 h. Bioassay dishes were scraped using a biospreader with 20 mL cold LB per plate, and the bacteria from the two dishes were collected into 50 mL conical tubes kept on ice. The tubes were centrifuged at 3900 rpm for 30 min, after which the supernatants were discarded and pellets were weighed. Each conical tube represented a single Maxiprep. Plasmid isolation was performed with the HiSpeed Plasmid Maxi Kit (12662, Qiagen). The sequencing library was prepared through PCR amplification, and the integrity of the amplified gRNA library was validated on the HiSeq2500 system (Illumina). HEK293T cells were employed for lentiviral gRNA library packaging. In brief, cells were seeded in 8 × T75 flasks at ~80% confluency. At 24 h post-seeding, co-transfection was performed with 33 µg Brunello gRNA library, 16.5 µg psPAX2, and 8.25 µg pMD2.G using PEI at 1:5 DNA:PEI ratio. The supernatants were harvested at both 48 h and 72 h post-transfection, pooled, and then filtered through a 0.45 μm SFCA filter. Aliquots were either used immediately or stored at −80 °C until further use.

### Genome-wide CRISPR screening

For each cell line screen, 120 million Cas9-expressing monoclonal cells were seeded into 5-layer flasks (353144, Corning). These cells were then transduced with titrated library virus stocks at a multiplicity of infection (MOI) of 0.3, ensuring single viral integrations at ~250× fold library coverage. After 48 h of infection, target cells were selected with puromycin (ant-pr-1, Invivogen) at a concentration of 2 µg/mL for MeT-5A and H28, and 3 µg/mL for H2052 and H2452 for 72 h. At least 20 million library infected cells (equivalent to 250× gRNA representation) were passaged in triplicate every 3–4 days and maintained in cell culture for 14 population doubling times. Cell pellets with at least 20 million cells were harvested at T0 (time zero following puromycin selection, input DNA) and T14 (14 doubling times) at which point the screen was completed.

### Genomic DNA sequencing

Genomic DNA from the cell pellets was extracted using the QIAamp Blood Maxi Kit (51192, Qiagen). To determine the gRNA representation in each sample, gRNA-targeted regions were amplified by PCR at 100X representation using NEBNext High-Fidelity 2X PCR Master Mix (M0541L, NEB) and Illumina TruSeq adapters P5 and P7 (25 µL of each 10 µM primer). To preserve sequence diversity in the PCR library, a P5 primer mix of at least eight primers with distinct stagger regions of different lengths was used. In addition, unique index sequences were introduced on the P7 primers, allowing for the simultaneous sequencing of different samples on the same flow cell. Primer sequences used in the amplification of sequence libraries are listed in Supplementary Table 3. The mastermix was prepared as 500 µL and divided into five tubes, each containing 100 µL. PCR was performed under the following conditions: 30 s at 98 °C; 28 cycles of 10 s at 98 °C, 30 s at 53 °C, 30 s at 72 °C; and 2 min at 72 °C. The library size was confirmed by gel electrophoresis. Amplified fragments were gel purified, quantified by NanoDrop 2000 (Thermo Fisher Scientific), and analyzed by Bioanalyzer (Agilent). Samples were pooled in equimolar quantities and sequenced on the HiSeq2500 system (Illumina).

### Analysis of CRISPR screen results

FastQC tool (v0.11.9) (https://www.bioinformatics.babraham.ac.uk/projects/fastqc/) was used for quality control of the raw FASTQ files, allowing the identification of low-quality reads and adapter content. The raw FASTQ files were processed with the Cutadapt tool (v4.5) to omit low-quality reads and to extract gRNA sequences using the following barcode sequence: GAAACACCG [75]. Subsequent processing involved the extraction of gRNA sequences and preparing the data for integration into the MAGeCK pipeline [13]. The MAGeCK software and algorithms were sourced from https://sourceforge.net/p/mageck/wiki/Home/. The instructions on running these algorithms were obtained from this repository and previously published protocols [76]. Following alignment of the trimmed raw data to the sequences of the Brunello library using the MAGeCK algorithm (v0.5.9), quantification of gRNA abundance in each sample and normalization of read counts were performed for downstream analysis. Robust Rank Aggregation (RRA) analysis, comparing T0 and T14 groups for each cell line independently, was conducted to identify the distribution of differentially enriched gRNAs. After the RRA analysis, quality control procedures and integrative analyses were executed using the MAGeCKFlute algorithm. MAGeCKFlute enables the comprehensive analysis of CRISPR screening data by applying MAGeCK or its extension MAGeCK-VISPR for target gene identification. Downstream pathway enrichment analysis was conducted using FluteRRA [76]. Log2 fold change (LFC) of gene scores was ranked and cut-off of LFC ≤ −1 was used for MeT-5A, H2052, and H2452 cell lines and LFC ≤ −0.5 was used for H28 cell line. For each gene, a CRISPR viability score was calculated, as previously suggested [77]. It is defined as the median of *z*-scored transformed LFC of the relative abundance of gRNAs targeting each gene. *Z*-scores were derived with the following equation: $Z-{score\; of\; each\; gRNA}=\frac{\left({gRNA\; fold\; change}\right)-{Mean}({gRNA\; fold\; change})}{{Standard\; deviation}({gRNA\; fold\; change})}$. DepMap data related to CRISPR screening experiments were retrieved from DepMap Public 23Q2+Score, Chronos at https://depmap.org/portal/download/ [16, 78].

### CRISPR/Cas9-mediated genome editing and ectopic expression of *BUB1*

Unique gRNA sequences targeting human BUB1 kinase were cloned into the lentiCRISPR v2 backbone. Lentiviral particles carrying these constructs were used to infect MeT-5A, H2052, H2452, and H28 cells, followed by puromycin selection for 72 h. Polyclonal cell lines with *BUB1* knockout were confirmed by western blotting. BUB1 knockout monoclonal cells were generated following transfection with pX459 vector (H2052 cells) or infection with lentiCRISPR v2 vector (H2452 cells). Engineered cells were seeded into 96-well plates at a density of one cell per well. Isogenic clones with BUB1 knockout or BUB1 wildtype background (the gRen control clones) were expanded and validated for loss of BUB1 protein by western blotting.

To achieve stable ectopic expression, the human BUB1 coding sequence was extracted from the pBI-GFP-Bub1-wt backbone (Addgene plasmid #16624) using NotI (R3189S, NEB) and BamHI (R3136S, NEB) restriction enzymes, and subcloned into the multiple cloning site (MCS) of the lentiviral overexpression vector, pHIV-Zsgreen (Addgene plasmid #18121). H2052, H2452 and H28 cells were separately infected with either the Empty vector or the BUB1 overexpression vector. Zsgreen-positive cells were enriched through FACS. Validation of BUB1 overexpression was performed through western blotting. For rescue experiments, gRNA-resistant cDNA of the *BUB1* gene was designed by introducing silent mutations on the critical “G” in the PAM motif (NGG) and on the targeting sequence of g2 (gRNA # 2). gRNA-resistant BUB1 coding sequence was amplified by overlapping PCR using the constructed overexpression vector as a template with the oligonucleotide sequences listed in Supplementary Table 2. Amplified sequence was digested with NotI and BamHI restriction enzymes, and cloned into predigested pHIV-Zsgreen. H2052, H2452 and H28 cells were separately infected with either the Empty vector or the gRNA-resistant BUB1 overexpression vector. Zsgreen-positive cells were enriched through FACS, followed by targeting the endogenous *BUB1* locus with relevant constructs. Validation of rescue for *BUB1* knockout was performed through western blotting.

### Western blot analysis

Cells were harvested by trypsinization or scraping, rinsed with ice-cold PBS, and lysed in modified RIPA Buffer [50 mM Tris pH 7.4, 150 mM NaCl, 1% Triton X-100, 0.5% Na-deoxycholate, 0.1% SDS, 1 mM EDTA, 1 mM EGTA, 10 mM β-glycerophosphate, 10 mM NaF, 1 mM PMSF, 1 mM Na-orthovanadate containing Leupeptin, Aprotinin, and Pepstatin (each with 10 µg/mL concentration)] for 30 min on ice. Following centrifugation at 11,000 × *g* for 30 min at 4 °C, the supernatants were collected, and the total protein concentrations were assessed using the Pierce BCA Protein Assay Kit (23227, Thermo Fisher Scientific). Protein samples (20 µg) were separated on 8–15% SDS-PAGE gels, followed by wet transfer to 0.20 µm nitrocellulose membranes (10600004, GE Healthcare) at 350 mA for 90 min using a wet transfer tank system (Bio-Rad). The membranes were blocked with 5% skim milk powder dissolved in TBS for 1 h at room temperature, and then incubated with primary antibodies overnight at 4 °C. The following primary antibodies were used: BUB1 (1/1000; ab195268, Abcam), Flag-M2 (1/1000; F1804, Sigma Aldrich), β-actin (1/1000; 3700T, Cell Signaling Technology), α-tubulin (1/1000; sc-32293, Santa Cruz Biotechnology), AURKA (1/1000; 07-648, Upstate), CDK2 (1/500; sc-6248, Santa Cruz Biotechnology), VPS37A (1/1000; sc-376978, Santa Cruz Biotechnology), CDC20 (1/200; sc-13162, Santa Cruz Biotechnology), Cyclin A (1/200; sc-596, Santa Cruz Biotechnology), Cyclin B (1/500; 4138, Cell Signaling Technology), p21 (1/1000; 10355-1-AP, Proteintech). Membranes were then incubated with anti-mouse secondary antibody tagged with IRDye 680LT fluorophores (1/10,000; 926-68022, LI-COR), anti-rabbit secondary antibody tagged with IRDye 800CW fluorophores (1/20,000; 926-32213, LI-COR), anti-mouse IgG, HRP-linked antibody (1/1000; 7076, Cell Signaling Technology), or anti-rabbit IgG, HRP-linked antibody (1/1000; 7074, Cell Signaling Technology) at room temperature for 1 h. After each incubation, the membranes were rinsed with TBS-T three times for 10 min each at room temperature. Images were obtained using the Odyssey CLx Imaging System and the Image Studio Lite software (LI-COR) or ChemiDoc MP Imaging System (BioRad) upon ECL (RPN2232, Amersham) visualization. β-actin or α-tubulin levels were studied as equal loading controls.

### Two-dimensional (2D) colony formation assay

Cells were inoculated into 12-well plates at a seeding density of 600 cells per well. The growth medium was refreshed every 3–4 days over a clonal growth of 10–14 days. Cells were then fixed with 3.7% formaldehyde for 20 min, followed by staining with crystal violet solution (0.5% w/v crystal violet powder dissolved in 20% ethanol) for 40 min at room temperature. After full decolorization through extensive rinsing with tap water, the plates were inverted and air-dried at room temperature. High-resolution images of the plates were obtained using the Odyssey CLx Imaging System, and the signal intensities were quantified using ImageJ 1.53e software [79]. To assess the effect of BAY-1816032 treatment on 2D colony formation, cells were treated once with three different concentrations of BAY-1816032 for 72 h. Medium was replaced with drug-free fresh medium every 3–4 days over a period of 10–14 days.

### Three-dimensional (3D) anchorage-independent growth assay

The bottom of the 12-well plate was coated with a mixture of 800 µL of equal volume of culture medium and 1% noble agar (w/v) (J10907-100gr, Alfa Aesar), providing even distribution, and then left to solidify in the hood at room temperature for 30 min. Cells with genetic manipulations were seeded at a density of 8000 cells/800 µL per well by mixing equal volume of the growth medium and 0.6% noble agar (w/v). The cell-agar mixture was left to solidify in the hood at room temperature for 15 min, followed by the addition of 800 µL medium into each well. Cells were allowed to grow for 21 days, with fresh medium replenished every 3–4 days. Colonies were visualized under a light microscope (Olympus CKX41) and quantified using ImageJ 1.53e software.

### MTT assay

Cells were plated at a density of 750 cells per well into 96-well plates. Following cell growth at 37 °C for 48 h, 96 h, 144 h, and 192 h 20 µL of thiazolyl blue tetrazolium bromide (MTT; 1334GR001, Neofroxx) labeling reagent was added to each well at a final concentration of 0.5 mg/mL and allowed to incubate for 4 h at 37 °C. Subsequently, the medium was discarded, and MTT crystals were solubilized with 100 µL DMSO (LC-5071.2, Neofroxx) for 45 min at room temperature. Absorbance signals were measured at 570 nm with a reference wavelength of 720 nm using the Multiskan GO Microplate Spectrophotometer (Thermo Fisher Scientific). To determine the IC50 values of BUB1 inhibitor, parental cells were seeded into 96-well plates as 1500 cells per well and cultured in complete growth medium for 24 h. The medium was then removed and replaced with different doses of BAY-1816032 ranging from 0.25 to 10 μM. The cells were incubated at 37 °C for 72 h. The MTT assay was performed as described above, and IC50 values were calculated using GraphPad Prism 8.0 software.

### Bromodeoxyuridine (BrdU) incorporation assay

Cells were plated on 18 mm round glass slides in 12-well plates at a density of 20,000 cells per well and allowed to grow for 48 h. Subsequently, cells were labeled with 30 μM BrdU (B5002, Sigma Aldrich) for 12 h, followed by rinsing with 1X PBS and fixation with ice-cold 70% ethanol for 10 min on ice. DNA was denatured with 2N HCl treatment for 30 min. Slides were then washed three times with PBS containing Tween 20 (PBS-T) for 5 min each with shaking. BrdU monoclonal antibody (1/1000; 5292, Cell Signaling Technology) diluted in PBS was applied to the slides and incubated for 2 h at room temperature. Slides were washed three times with PBS-T for 5 min each with shaking. Cells were then labeled with either donkey anti-mouse Alexa Fluor 488 (ab150105, Abcam) or goat anti-mouse Alexa Fluor 594 (8890S, Cell Signaling Technology) for 1 h at room temperature. Finally, slides were rinsed three times with PBS-T for 5 min each with shaking. Nuclei were counterstained with DAPI (1322MG005, Neofroxx). Coverslips were mounted onto glass slides. Images were acquired with a fluorescence microscope (Olympus BX61). The percentage of proliferative cells was calculated as the number of BrdU-labeled cells over the total number of DAPI-stained nuclei.

### Senescence-associated beta-galactosidase (SA-β-Gal) assay

Cells were seeded into 12-well plates at a density of 20,000 cells per well and allowed to grow for 48 h. Cells were fixed with 500 µL fixative solution (0.2% glutaraldehyde solution, 1.85% formaldehyde in PBS) for 10 min at room temperature. Cells were washed twice with 1X PBS and treated with staining solution (40 mM citric acid/Na phosphate buffer, 5 mM potassium ferrocyanide, 5 mM potassium ferricyanide, 150 mM NaCl, 2 mM MgCl_2_, 1 mg/mL X-gal) for 12 h at 37 °C. Blue staining was monitored under a phase contrast microscope (Olympus IX71).

### Cell cycle analysis and apoptosis assay

To analyze cell cycle distribution by propidium iodide (PI) staining, cells were seeded into 6-well plates and allowed to grow for 48 h. After incubation, cells were collected by trypsinization and resuspended in 300 µL PBS and 700 µL 100% ice-cold ethanol and fixed for 15 min on ice. Fixed cells were collected by centrifugation at 1200 rpm for 15 min at 4 °C and resuspended in 70 µL PI solution (50 μg/mL propidium iodide, 0.1 mg/mL RNase A and 0.05% Triton X-100), and incubated for 40 min at 37 °C by vortexing every 10 min. After centrifugation at 6000 rpm for 5 min at 4 °C, stained cells were transferred to polystyrene tubes in 100 μL PBS and analyzed using flow cytometer (LSRFortessa, BD). Data was processed using FlowJo v10.8.0 software (BD Biosciences).

FITC Annexin V Apoptosis Detection Kit with PI (640914, Biolegend) was used for apoptosis analysis. Exponentially growing cells were collected and washed with ice-cold PBS. After centrifugation, cells were resuspended in 30 µL of Annexin V Binding Buffer, and 1 µL of Annexin V and 2 µL of PI solution were added onto each sample and incubated for 15 min at room temperature. Cells were transferred to polystyrene tubes with an additional 120 µL of Annexin V Binding Buffer and analyzed using a flow cytometer. Apoptotic cells were analyzed by FlowJo software (v10.8.0). To test the effect of BAY-1816032 on cell cycle distribution and apoptosis, cells were treated with indicated concentrations of BAY-1816032 for 72 h.

### Transwell migration and invasion assays

For migration assay, cells were loaded onto the upper well of the cell culture inserts (353097, Falcon or 37224, SPL) in a 24-well plate at a density of 25,000 cells in 200 μL serum-free medium. The lower well was filled with 600 μL of RPMI-1640 containing 10% FBS, and the cells were incubated for 48 h. After incubation, the medium was aspirated and the chambers were washed twice with 1X PBS. Cells were fixed with 3.7% formaldehyde for 2 min and washed twice with 1X PBS. After permeabilization with 100% methanol for 20 min, chambers were washed twice with 1X PBS and stained with crystal violet for 15 min. Subsequently, crystal violet stain was removed and chambers were washed twice with 1X PBS. For invasion assay, chambers were coated with Matrigel basement membrane matrix (356234, Corning) diluted in serum-free RPMI-1640 (1–40 ratio, v/v) in a final volume of 50 μL for each well and incubated for 2 h at 37 °C. The remaining liquid was removed from the permeable support membrane without disturbing the layer of matrigel matrix on the membrane prior to cell seeding. Non-migrating/non-invading cells were scraped off with cotton swabs. Migrated/invaded cells were captured under a light microscope and quantified by manual counting. To test the effect of BAY-1816032 on migration and/or invasion capacity, cell loading to the transwell inserts was performed after 3 days of inhibitor treatment.

### Spheroid formation assay

To evaluate spheroid formation in BUB1 knockout cells, the hanging drop cell culture method was utilized as previously described [80]. Briefly, 1000 cells were suspended in 20 µL droplets and pipetted onto the inverted lid of a 10 cm dish. To minimize evaporation, 1x PBS was added to the base of the dish prior to carefully sealing the lid. After 3 days of incubation, spheroid formation was visualized using Stereo microscopy equipped with 5× zoom.

To assess the impact of pharmacological BUB1 inhibition on spheroid growth, cells were seeded into ultra-low attachment 96-well plates at 5000 cells per well in 200 µL of medium. Plates were centrifuged at 800 rpm for 5 min at room temperature to promote cell aggregation [81]. Following a 3-day incubation to allow spheroid formation, 100 µL of medium was carefully removed, and BAY-181603 was applied at IC50 and higher concentrations. Spheroids were treated for 7 days and imaged using a light microscope (Olympus CKX41). Colony sizes were measured using ImageJ 1.53e software.

### MPM samples and tissue microarray

TMA containing 40 cases of MPM, other mesothelioma tissues, and normal mesothelium tissues with duplicate cores per case (MS801b) was obtained from Tissue Array (Rockville, MD, USA). Tissue sections were stained under the guidance of a pathologist who was blinded to sample identity, following the standard immunohistochemical staining (IHC) procedure involving deparaffinization, rehydration, and heat-induced antigen retrieval (Ventana HE 600, Roche). The rabbit recombinant monoclonal BUB1 (EPR18947) antibody (ab195268, Abcam) was applied at an optimized 1/50 dilution, and counterstaining was performed with hematoxylin. High-resolution microscopic images were captured using the BX61 microscope with 40× and 100× objectives and a DP72 camera (Olympus). BUB1 antibody showed both nuclear and cytoplasmic brown staining with a range of intensities. The scoring of IHC staining for each core was conducted blindly, without access to the related clinical data. The IRS was derived from the multiplication of the ordinal scores assessing both the proportion of positive cells and the intensity of immunostaining [82]. The IRS score was categorized as follows: no staining (0–1), mild staining (2–3), moderate staining (4–8), and strong staining (9–12), and calculated by multiplying the proportion (0: no staining, 1: 1%–10%, 2: 11%–50%, 3: 51%–80%, and 4: 81%–100%) with the intensity (0: negative, 1: mild, 2: moderate, and 3: intense).

### Immunofluorescence staining

Cells were plated on 12 mm coverslips in 24-well plates at a density of 15,000 cells per well. After 36 h, H2052 and H2452 cells were synchronized with a 12 h thymidine block (2 mM for H2052 and 4 mM for H2452) followed by a 12 h release in complete culture medium, while H28 cells were synchronized with a 12 h thymidine block (2 mM) followed by treatment with 150 mM nocodazole for 12 h. Cells were fixed using PTEMF buffer (20 mM PIPES, pH 6.8, 0.2% Triton X-100, 10 mM EGTA, 1 mM MgCl2, 4% formaldehyde) and subjected to immunofluorescence staining as previously described [30]. Following antibodies were used: MAD1 (1/200; 18322-1-AP, Proteintech), MAD2 (1/200; 10337-1-AP, Proteintech) or SGO1 (1/200; 16977-1-AP, Proteintech). DAPI was used for nuclear counterstaining, and coverslips were mounted for imaging. Mitotic cells were visualized using a Zeiss Axio Observer 7 with Apotome 3 and a 63× oil objective for high-resolution z-stack images.

### Time-lapse cell tracking

Mitotic progression in BUB1-deficient cells was monitored as previously described [83]. Briefly, H2452 cells were seeded into 12-well plates. After 18 h, chromosomes were labeled with 0.005 µg/mL Hoechst 33340 (H3570, Invitrogen). Time-lapse imaging was conducted using Leica SP8 DMi8 confocal laser scanning microscope, capturing z-stacks every 10 min from randomly selected fields over a 20 h period.

### BUB1 expression in human MPM and genomic analyses

BUB1 expression levels in MPM tumors with comparison to normal tissues were accessed in the Gene Expression Omnibus (GEO) [84] database. Four independent cohorts of gene expression microarray datasets of MPM tissues or cell lines, and normal mesothelium and lung tissues or cell lines (GSE2549, GSE42977, GSE51024, and GSE117668) were retrieved from the GEO database. Specifically, the GSE2549 dataset includes 40 MPM tissues and 9 non-tumoral tissues, comprising 5 normal pleura and 4 normal lung tissues. The GSE42977 dataset consists of 39 MPM tissues and 9 non-tumoral tissues, with 7 normal pleura and 2 normal lung tissues. The GSE51024 dataset consists of 55 MPM tissues along with 41 paired normal tissues. The GSE117668 dataset consists of 12 MPM cell lines and 4 healthy stromal cell lines. Samples in the datasets were processed on Affymetrix Human Genome U133A Array, Illumina HumanRef-6 v2.0 expression beadchip, Affymetrix Human Genome U133 Plus 2.0 Array, and Affymetrix Human Gene 2.0 ST Array platforms, respectively. The datasets were processed and normalized using the RMA normalization algorithm. Box plots demonstrating differential expression of BUB1 were plotted using R script programming. DepMap Expression Public 23Q4 data related to gene expression profiles were extracted from the Cancer Cell Line Encyclopedia datasets [85]. Mann–Whitney *U* test was used to compare statistical differences between the two groups.

### RNA isolation and quantitative real-time PCR

Total RNA was isolated using the NucleoSpin RNA kit (740955, MN) following the manufacturer’s protocol. Subsequently, 1 μg of RNA was reverse transcribed into cDNA using the iScript cDNA Synthesis Kit (1708891, Bio-Rad) as per the manufacturer’s instructions. Quantitative real-time PCR (qRT-PCR) was carried out with gene-specific primers and the BrightGreen qPCR MasterMix kit (MasterMix-S-XL, ABM) on an Applied Biosystems 7500 Fast Real-Time PCR System. Target mRNA levels were normalized to β-actin mRNA using the 2^−ΔΔCt^ method. The primers used in qRT-PCR reactions are listed in Table 4.

### RNA-sequencing and analysis

Transcriptome profiling of H2052, H2452, and H28 BUB1 KO cells, relative to BUB1 WT clones, was performed in duplicates on the Illumina NovaSeqX platform (Macrogen, Seoul, Republic of Korea). Approximately 100 million paired-end reads (151-bp) per sample were generated. Quality control was performed using FASTQC (v0.11.7) and low-quality reads were removed using Cutadapt (v3.7). High-quality reads were aligned to the GRCh38 human reference genome (Gencode release 34) using the Rsubread package (v1.34.7). Gene expression levels were quantified with the featureCounts function of Rsubread and converted to counts per million. Differential expression analysis between groups was performed using the edgeR package (v3.24.3) in the R computational environment (v3.5.2). Gene expression data were visualized using the ggplot2 package in the R environment. Genes with significant expression changes were identified based on *p* values < 0.05.

### Gene set enrichment analysis

To correlate the level of BUB1 expression with target gene set enrichment in MPM samples, the GSE2549, GSE29211, GSE42977, and GSE163722 datasets, as well as the BUB1 KO H2052, H2452 and H28 clones, were analyzed. The GSE29211 dataset consists of 53 MPM tissues and the GSE163722 dataset contains 129 MPM tumor tissues. All GEO datasets were individually divided into two cohorts based on the median value of *BUB1* expression. These cohorts were represented as *BUB1* High and *BUB1* Low groups. Differentially expressed genes (DEGs) were evaluated for enriched gene sets using the GSEA tool from the Broad Institute. As for the GSEA, a list of DEGs with phenotype labels “High” vs. “Low” or “BUB1 WT” vs. “BUB1 KO” was uploaded to the GSEA tool [86], and normalized enrichment scores (NES), nominal *p* values (*p*), and false discovery rates (FDR) were computed after 1000 random permutations of the gene set. Several gene sets and datasets were used to demonstrate the proliferative implications of elevated *BUB1* expression in MPM tumors. The MSigDB gene set database was set to the *collection h.all.v2023.2.Hs.symbols.gmt*, where H stands for “Hallmark”. Among the hallmark collections, the HALLMARK_E2F_TARGETS, HALLMARK_MYC_TARGETS_V1 and HALLMARK_G2M_CHECKPOINT were used, all of which have 200 members that are linked to 200 genes. Similarly, a gene set related to cell motility was examined using a gene set obtained from the MSigDB gene set database. This collection contains the HALLMARK_EPITHELIAL_MESENCHYMAL_TRANSITION gene set, which includes 200 members that correspond to epithelial cell migration target genes. Additionally, the MSigDB collections *c2.all.v2024.1.Hs.symbols.gmt* and *c5.go.v2024.1.Hs.symbols.gmt* were used to explore other biological processes and phenotypes. NES > 1, *p* value < 0.05, and an FDR < 0.25 were considered to be statistically significant.

### Statistical analysis

To assess the efficacy of CRISPR screening in different cell lines, the receiver operating characteristic (ROC)—area under the curve (AUC) analysis [87] was performed and true positive and false positive rates for a predefined set of essential (1580) and nonessential genes (927), were defined, respectively. The gene list was extracted from a previously published CRISPR screening study [14]. The ROC-AUC plots were generated by constructing a confusion matrix using the pROC R package, and thus calculating true positive and false positive rates at various thresholds based on the LFC of all genes targeting each of the genes in the essential and nonessential gene lists. The data for each group are reported as the mean ± standard deviation (SD). Two-tailed unpaired Student’s *t*-test was employed for experiments involving two experimental conditions or comparisons to evaluate the difference between means. Statistical significance in TMA analysis was assessed using the Mann–Whitney *U* method. Pearson correlation test was used to determine correlation of gene expression. The statistical significance between more than two conditions or comparisons was calculated by one-way analysis of variance (ANOVA) followed by Dunnet’s multiple comparisons test. Details of statistical significance are provided in the figure legends. Statistical analyses were conducted using GraphPad Prism version 8.0 (GraphPad Software, San Diego, USA). The results with *p* values less than 0.05 were considered statistically significant, and significance levels were set as follows: *p < 0.05, **p < 0.01, ***p < 0.001.

## Supplementary information

Supplementary Figure Legends

Supplementary Tables

Supplementary Figure 1

Supplementary Figure 2

Supplementary Figure 3

Supplementary Figure 4

Supplementary Figure 5

Supplementary Figure 6

Supplementary Figure 7

Supplementary Figure 8

Supplementary Figure 9

Supplementary Figure 10

Uncropped WB

## Data Availability

The authors state that the data underlying the findings of this study are provided in the article and the Supplementary Information. Bulk RNA-Seq data have been deposited in the Gene Expression Omnibus (GEO) repository under accession number GSE292620. Additional data can be obtained from the corresponding author upon reasonable request.

## References

[1] Sung H, Ferlay J, Siegel RL, Laversanne M, Soerjomataram I, Jemal A, et al. Global Cancer Statistics 2020: GLOBOCAN estimates of incidence and mortality worldwide for 36 cancers in 185 countries. CA Cancer J Clin. 2021;71:209–49. 33538338 10.3322/caac.21660

[2] Beebe-Dimmer JL, Fryzek JP, Yee CL, Dalvi TB, Garabrant DH, Schwartz AG, et al. Mesothelioma in the United States: a Surveillance, Epidemiology, and End Results (SEER)-Medicare investigation of treatment patterns and overall survival. Clin Epidemiol. 2016;8:743–50. 27822122 10.2147/CLEP.S105396 PMC5087771

[3] Cakiroglu E, Senturk S. Genomics and functional genomics of malignant pleural mesothelioma. Int J Mol Sci. 2020;21:6342. 32882916 10.3390/ijms21176342 PMC7504302

[4] Scherpereel A, Wallyn F, Albelda SM, Munck C. Novel therapies for malignant pleural mesothelioma. Lancet Oncol. 2018;19:e161–72. 29508763 10.1016/S1470-2045(18)30100-1

[5] Bronte G, Incorvaia L, Rizzo S, Passiglia F, Galvano A, Rizzo F, et al. The resistance related to targeted therapy in malignant pleural mesothelioma: Why has not the target been hit yet? Crit Rev Oncol Hematol. 2016;107:20–32. 27823648 10.1016/j.critrevonc.2016.08.011

[6] Nicolini F, Bocchini M, Bronte G, Delmonte A, Guidoboni M, Crinò L, et al. Malignant pleural mesothelioma: state-of-the-art on current therapies and promises for the future. Front Oncol. 2019;9:1519. 32039010 10.3389/fonc.2019.01519 PMC6992646

[7] Przybyla L, Gilbert LA. A new era in functional genomics screens. Nat Rev Genet. 2022;23:89–103. 34545248 10.1038/s41576-021-00409-w

[8] Shalem O, Sanjana NE, Zhang F. High-throughput functional genomics using CRISPR-Cas9. Nat Rev Genet. 2015;16:299–311. 25854182 10.1038/nrg3899 PMC4503232

[9] Zhang Y, Song C, Wang L, Jiang H, Zhai Y, Wang Y, et al. Zombies never die: the double life Bub1 lives in mitosis. Front Cell Dev Biol. 2022;10. 870745. 35646932 10.3389/fcell.2022.870745 PMC9136299

[10] Doench JG, Fusi N, Sullender M, Hegde M, Vaimberg EW, Donovan KF, et al. Optimized sgRNA design to maximize activity and minimize off-target effects of CRISPR-Cas9. Nat Biotechnol. 2016;34:184–91. 26780180 10.1038/nbt.3437 PMC4744125

[11] Jagadeeswaran R, Ma PC, Seiwert TY, Jagadeeswaran S, Zumba O, Nallasura V, et al. Functional analysis of c-Met/hepatocyte growth factor pathway in malignant pleural mesothelioma. Cancer Res. 2006;66:352–61. 16397249 10.1158/0008-5472.CAN-04-4567

[12] Lechner JF, Tokiwa T, LaVeck M, Benedict WF, Banks-Schlegel S, Yeager H, et al. Asbestos-associated chromosomal changes in human mesothelial cells. Proc Natl Acad Sci USA. 1985;82:3884–8. 2987952 10.1073/pnas.82.11.3884 PMC397893

[13] Li W, Xu H, Xiao T, Cong L, Love MI, Zhang F, et al. MAGeCK enables robust identification of essential genes from genome-scale CRISPR/Cas9 knockout screens. Genome Biol. 2014;15. 554. 25476604 10.1186/s13059-014-0554-4 PMC4290824

[14] Hart T, Chandrashekhar M, Aregger M, Steinhart Z, Brown KR, MacLeod G, et al. High-resolution CRISPR screens reveal fitness genes and genotype-specific cancer liabilities. Cell. 2015;163:1515–26. 26627737 10.1016/j.cell.2015.11.015

[15] Sherman BT, Hao M, Qiu J, Jiao X, Baseler MW, Lane HC, et al. DAVID: a web server for functional enrichment analysis and functional annotation of gene lists (2021 update). Nucleic Acids Res. 2022;50:W216–21. 35325185 10.1093/nar/gkac194 PMC9252805

[16] Dempster JM, Boyle I, Vazquez F, Root DE, Boehm JS, Hahn WC, et al. Chronos: a cell population dynamics model of CRISPR experiments that improves inference of gene fitness effects. Genome Biol. 2021;22. 343. 34930405 10.1186/s13059-021-02540-7 PMC8686573

[17] Miralaei N, Majd A, Ghaedi K, Peymani M, Safaei M. Integrated pan-cancer of AURKA expression and drug sensitivity analysis reveals increased expression of AURKA is responsible for drug resistance. Cancer Med. 2021;10:6428–41. 34337875 10.1002/cam4.4161 PMC8446408

[18] Wang L, Arras J, Katsha A, Hamdan S, Belkhiri A, Ecsedy J, et al. Cisplatin-resistant cancer cells are sensitive to Aurora kinase A inhibition by alisertib. Mol Oncol. 2017;11:981–95. 28417568 10.1002/1878-0261.12066 PMC5537695

[19] Yan M, Wang C, He B, Yang M, Tong M, Long Z, et al. Aurora-A kinase: a potent oncogene and target for cancer therapy. Med Res Rev. 2016;36:1036–79. 27406026 10.1002/med.21399

[20] Tadesse S, Anshabo AT, Portman N, Lim E, Tilley W, Caldon CE, et al. Targeting CDK2 in cancer: challenges and opportunities for therapy. Drug Discov Today. 2020;25:406–13. 31839441 10.1016/j.drudis.2019.12.001

[21] Wang J, Yang T, Xu G, Liu H, Ren C, Xie W, et al. Cyclin-dependent kinase 2 promotes tumor proliferation and induces radio resistance in glioblastoma. Transl Oncol. 2016;9:548–56. 27863310 10.1016/j.tranon.2016.08.007 PMC5118617

[22] Yamamoto H, Monden T, Miyoshi H, Izawa H, Ikeda K, Tsujie M, et al. Cdk2/cdc2 expression in colon carcinogenesis and effects of cdk2/cdc2 inhibitor in colon cancer cells. Int J Oncol. 1998;13:233–9. 9664116 10.3892/ijo.13.2.233

[23] Xu H, Miao ZF, Wang ZN, Zhao TT, Xu YY, Song YX, et al. HCRP1 downregulation confers poor prognosis and induces chemoresistance through regulation of EGFR-AKT pathway in human gastric cancer. Virchows Arch. 2017;471:743–51. 28963677 10.1007/s00428-017-2237-5

[24] Yang W, Wang JG, Wang Q, Qin Y, Lin X, Zhou D, et al. Decreased HCRP1 promotes breast cancer metastasis by enhancing EGFR phosphorylation. Biochem Biophys Res Commun. 2016;477:222–8. 27311861 10.1016/j.bbrc.2016.06.046

[25] Senturk S, Shirole NH, Nowak DG, Corbo V, Pal D, Vaughan A, et al. Rapid and tunable method to temporally control gene editing based on conditional Cas9 stabilization. Nat Commun. 2017;8:14370. 28224990 10.1038/ncomms14370 PMC5322564

[26] Cahill DP, Lengauer C, Yu J, Riggins GJ, Willson JK, Markowitz SD, et al. Mutations of mitotic checkpoint genes in human cancers. Nature. 1998;392:300–3. 9521327 10.1038/32688

[27] Ricke RM, Jeganathan KB, van Deursen JM. Bub1 overexpression induces aneuploidy and tumor formation through Aurora B kinase hyperactivation. J Cell Biol. 2011;193:1049–64. 21646403 10.1083/jcb.201012035 PMC3115799

[28] Fujibayashi Y, Isa R, Nishiyama D, Sakamoto-Inada N, Kawasumi N, Yamaguchi J, et al. Aberrant BUB1 overexpression promotes mitotic segregation errors and chromosomal instability in multiple myeloma. Cancers. 2020;12:2206. 32781708 10.3390/cancers12082206 PMC7464435

[29] Siemeister G, Mengel A, Fernández-Montalván AE, Bone W, Schröder J, Zitzmann-Kolbe S, et al. Inhibition of BUB1 kinase by BAY 1816032 sensitizes tumor cells toward taxanes, ATR, and PARP inhibitors in vitro and in vivo. Clin Cancer Res. 2019;25:1404–14. 30429199 10.1158/1078-0432.CCR-18-0628

[30] Baron AP, von Schubert C, Cubizolles F, Siemeister G, Hitchcock M, Mengel A, et al. Probing the catalytic functions of Bub1 kinase using the small molecule inhibitors BAY-320 and BAY-524. eLife. 2016;5:e12187. 26885717 10.7554/eLife.12187 PMC4769170

[31] Mitsopoulos C, Di Micco P, Fernandez EV, Dolciami D, Holt E, Mica IL, et al. canSAR: update to the cancer translational research and drug discovery knowledgebase. Nucleic Acids Res. 2021;49:D1074–82. 33219674 10.1093/nar/gkaa1059 PMC7778970

[32] Senturk S, Mumcuoglu M, Gursoy-Yuzugullu O, Cingoz B, Akcali KC, Ozturk M. Transforming growth factor-beta induces senescence in hepatocellular carcinoma cells and inhibits tumor growth. Hepatology. 2010;52:966–74. 20583212 10.1002/hep.23769

[33] Zhu LJ, Pan Y, Chen XY, Hou PF. BUB1 promotes proliferation of liver cancer cells by activating SMAD2 phosphorylation. Oncol Lett. 2020;19:3506–12. 32269624 10.3892/ol.2020.11445 PMC7114935

[34] Bolanos-Garcia VM, Blundell TL. BUB1 and BUBR1: multifaceted kinases of the cell cycle. Trends Biochem Sci. 2011;36:141–50. 20888775 10.1016/j.tibs.2010.08.004 PMC3061984

[35] Gordon GJ, Rockwell GN, Jensen RV, Rheinwald JG, Glickman JN, Aronson JP, et al. Identification of novel candidate oncogenes and tumor suppressors in malignant pleural mesothelioma using large-scale transcriptional profiling. Am J Pathol. 2005;166:1827–40. 15920167 10.1016/S0002-9440(10)62492-3 PMC1363736

[36] Bott M, Brevet M, Taylor BS, Shimizu S, Ito T, Wang L, et al. The nuclear deubiquitinase BAP1 is commonly inactivated by somatic mutations and 3p21.1 losses in malignant pleural mesothelioma. Nat Genet. 2011;43:668–72. 21642991 10.1038/ng.855 PMC4643098

[37] De Rienzo A, Richards WG, Yeap BY, Coleman MH, Sugarbaker PE, Chirieac LR, et al. Sequential binary gene ratio tests define a novel molecular diagnostic strategy for malignant pleural mesothelioma. Clin Cancer Res. 2013;19:2493–502. 23493352 10.1158/1078-0432.CCR-12-2117 PMC3644001

[38] De Rienzo A, Coleman MH, Yeap BY, Severson DT, Wadowski B, Gustafson CE, et al. Association of RERG expression with female survival advantage in malignant pleural mesothelioma. Cancers. 2021;13:565. 33540554 10.3390/cancers13030565 PMC7867122

[39] Gjoerup OV, Wu J, Chandler-Militello D, Williams GL, Zhao J, Schaffhausen B, et al. Surveillance mechanism linking Bub1 loss to the p53 pathway. Proc Natl Acad Sci USA. 2007;104:8334–9. 17488820 10.1073/pnas.0703164104 PMC1895950

[40] Nyati S, Schinske-Sebolt K, Pitchiaya S, Chekhovskiy K, Chator A, Chaudhry N, et al. The kinase activity of the Ser/Thr kinase BUB1 promotes TGF-β signaling. Sci Signal. 2015;8:ra1. 25564677 10.1126/scisignal.2005379 PMC4440544

[41] Qiu J, Zhang S, Wang P, Wang H, Sha B, Peng H, et al. BUB1B promotes hepatocellular carcinoma progression via activation of the mTORC1 signaling pathway. Cancer Med. 2020;9:8159–72. 32977361 10.1002/cam4.3411 PMC7643650

[42] Hanahan D, Weinberg RA. Hallmarks of cancer: the next generation. Cell. 2011;144:646–74. 21376230 10.1016/j.cell.2011.02.013

[43] Ramundo V, Zanirato G, Aldieri E. The epithelial-to-mesenchymal transition (EMT) in the development and metastasis of malignant pleural mesothelioma. Int J Mol Sci. 2021;22:12216. 34830097 10.3390/ijms222212216 PMC8621591

[44] Schramm A, Opitz I, Thies S, Seifert B, Moch H, Weder W, et al. Prognostic significance of epithelial-mesenchymal transition in malignant pleural mesothelioma. Eur J Cardiothorac Surg. 2010;37:566–72. 19781955 10.1016/j.ejcts.2009.08.027

[45] Foty R. A simple hanging drop cell culture protocol for generation of 3D spheroids. J Vis Exp. 2011;51:2720. 10.3791/2720 PMC3197119 21587162

[46] Suraokar MB, Nunez MI, Diao L, Chow CW, Kim D, Behrens C, et al. Expression profiling stratifies mesothelioma tumors and signifies deregulation of spindle checkpoint pathway and microtubule network with therapeutic implications. Ann Oncol. 2014;25:1184–92. 24669013 10.1093/annonc/mdu127 PMC4037861

[47] Kim T, Gartner A. Bub1 kinase in the regulation of mitosis. Anim Cells Syst. 2021;25:1–10. 10.1080/19768354.2021.1884599 PMC7935115 33717411

[48] Zhang G, Kruse T, López-Méndez B, Sylvestersen KB, Garvanska DH, Schopper S, et al. Bub1 positions Mad1 close to KNL1 MELT repeats to promote checkpoint signalling. Nat Commun. 2017;8. 15822. 28604727 10.1038/ncomms15822 PMC5472792

[49] Perera D, Taylor SS. Sgo1 establishes the centromeric cohesion protection mechanism in G2 before subsequent Bub1-dependent recruitment in mitosis. J Cell Sci. 2010;123:653–9. 20124418 10.1242/jcs.059501

[50] Tang Z, Shu H, Oncel D, Chen S, Yu H. Phosphorylation of Cdc20 by Bub1 provides a catalytic mechanism for APC/C inhibition by the spindle checkpoint. Mol Cell. 2004;16:387–97. 15525512 10.1016/j.molcel.2004.09.031

[51] Yap TA, Aerts JG, Popat S, Fennell DA. Novel insights into mesothelioma biology and implications for therapy. Nat Rev Cancer. 2017;17:475–88. 28740119 10.1038/nrc.2017.42

[52] Lee YT, Tan YJ, Oon CE. Molecular targeted therapy: treating cancer with specificity. Eur J Pharmacol. 2018;834:188–96. 30031797 10.1016/j.ejphar.2018.07.034

[53] Fellmann C, Gowen BG, Lin PC, Doudna JA, Corn JE. Cornerstones of CRISPR-Cas in drug discovery and therapy. Nat Rev Drug Discov. 2017;16:89–100. 28008168 10.1038/nrd.2016.238 PMC5459481

[54] Cicenas J, Kalyan K, Sorokinas A, Stankunas E, Levy J, Meskinyte I, et al. Roscovitine in cancer and other diseases. Ann Transl Med. 2015;3:135. 26207228 10.3978/j.issn.2305-5839.2015.03.61 PMC4486920

[55] Fukuoka M, Kodama T, Murai K, Hikita H, Sometani E, Sung J, et al. Genome-wide loss-of-function genetic screen identifies INSIG2 as the vulnerability of hepatitis B virus-integrated hepatoma cells. Cancer Sci. 2024;115:859–70. 38287498 10.1111/cas.16070 PMC10920982

[56] Zhao N, Guo M, Wang K, Zhang C, Liu X. Identification of pan-cancer prognostic biomarkers through integration of multi-omics data. Front Bioeng Biotechnol. 2020;8:268. 32300588 10.3389/fbioe.2020.00268 PMC7142216

[57] Du R, Huang C, Liu K, Li X, Dong Z. Targeting AURKA in cancer: molecular mechanisms and opportunities for cancer therapy. Mol Cancer. 2021;20. 15. 33451333 10.1186/s12943-020-01305-3 PMC7809767

[58] Chang L, Ruiz P, Ito T, Sellers WR. Targeting pan-essential genes in cancer: challenges and opportunities. Cancer Cell. 2021;39:466–79. 33450197 10.1016/j.ccell.2020.12.008 PMC8157671

[59] Lorenzini E, Torricelli F, Zamponi R, Donati B, Manicardi V, Sauta E, et al. KAP1 is a new non-genetic vulnerability of malignant pleural mesothelioma (MPM). NAR Cancer. 2022;4. zcac024. 35910692 10.1093/narcan/zcac024 PMC9336180

[60] Hiltbrunner S, Fleischmann Z, Sokol ES, Zoche M, Felley-Bosco E, Curioni-Fontecedro A. Genomic landscape of pleural and peritoneal mesothelioma tumours. Br J Cancer. 2022;127:1997–2005. 36138075 10.1038/s41416-022-01979-0 PMC9681755

[61] Piao J, Zhu L, Sun J, Li N, Dong B, Yang Y, et al. High expression of CDK1 and BUB1 predicts poor prognosis of pancreatic ductal adenocarcinoma. Gene. 2019;701:15–22. 30898709 10.1016/j.gene.2019.02.081

[62] Han JY, Han YK, Park GY, Kim SD, Lee CG. Bub1 is required for maintaining cancer stem cells in breast cancer cell lines. Sci Rep. 2015;5:15993. 26522589 10.1038/srep15993 PMC4629164

[63] Zhang J, Wei W, Zhong Q, Feng K, Yang R, Jiang Q. Budding uninhibited by benzimidazoles 1 promotes cell proliferation, invasion, and epithelial-mesenchymal transition via the Wnt/β-catenin signaling in glioblastoma. Heliyon. 2023;9:e16996. 37342577 10.1016/j.heliyon.2023.e16996 PMC10277463

[64] Jiang W, Yu Y, Bhandari A, Hirachan S, Dong X, Huang X, et al. Budding uninhibited by benzimidazoles 1 might be a poor prognosis biomarker promoting the progression of papillary thyroid cancer. Environ Toxicol. 2023;38:2047–56. 37163344 10.1002/tox.23812

[65] Mengyan X, Kun D, Xinming J, Yutian W, Yongqian S. Identification and verification of hub genes associated with the progression of non-small cell lung cancer by integrated analysis. Front Pharmacol. 2022;13:997842. 36176446 10.3389/fphar.2022.997842 PMC9513139

[66] Kim T, Gartner A. Bub1 kinase in the regulation of mitosis. Animal Cells Syst. 2021;25:1–10. 10.1080/19768354.2021.1884599 PMC7935115 33717411

[67] Ganem NJ, Cornils H, Chiu SY, O’Rourke KP, Arnaud J, Yimlamai D, et al. Cytokinesis failure triggers hippo tumor suppressor pathway activation. Cell. 2014;158:833–48. 25126788 10.1016/j.cell.2014.06.029 PMC4136486

[68] Hégarat N, Crncec A, Suarez Peredo Rodriguez MF, Echegaray Iturra F, Gu Y, Busby O, et al. Cyclin A triggers mitosis either via the greatwall kinase pathway or Cyclin B. EMBO J. 2020;39:e104419. 32350921 10.15252/embj.2020104419 PMC7265243

[69] Kreis NN, Louwen F, Yuan J. Less understood issues: p21(Cip1) in mitosis and its therapeutic potential. Oncogene. 2015;34:1758–67. 24858045 10.1038/onc.2014.133

[70] Zhou R, Liu M, Li M, Peng Y, Zhang X. BUB1 as a novel marker for predicting the immunotherapy efficacy and prognosis of breast cancer. Transl Cancer Res. 2024;13:4534–54. 39430818 10.21037/tcr-24-704 PMC11483447

[71] Martinez MJ, Lyles RDZ, Peinetti N, Grunfeld AM, Burnstein KL. Inhibition of the serine/threonine kinase BUB1 reverses taxane resistance in prostate cancer. iScience. 2023;26:107681. 37705955 10.1016/j.isci.2023.107681 PMC10495664

[72] Ran FA, Hsu PD, Wright J, Agarwala V, Scott DA, Zhang F. Genome engineering using the CRISPR-Cas9 system. Nat Protoc. 2013;8:2281–308. 24157548 10.1038/nprot.2013.143 PMC3969860

[73] Nowak DG, Katsenelson KC, Watrud KE, Chen M, Mathew G, D’Andrea VD, et al. The PHLPP2 phosphatase is a druggable driver of prostate cancer progression. J Cell Biol. 2019;218:1943–57. 31092557 10.1083/jcb.201902048 PMC6548123

[74] Sanson KR, Hanna RE, Hegde M, Donovan KF, Strand C, Sullender ME, et al. Optimized libraries for CRISPR-Cas9 genetic screens with multiple modalities. Nat Commun. 2018;9:5416. 30575746 10.1038/s41467-018-07901-8 PMC6303322

[75] Martin M. Cutadapt removes adapter sequences from high-throughput sequencing reads. EMBnet j. 2011;17:10.

[76] Wang B, Wang M, Zhang W, Xiao T, Chen CH, Wu A, et al. Integrative analysis of pooled CRISPR genetic screens using MAGeCKFlute. Nat Protoc. 2019;14:756–80. 30710114 10.1038/s41596-018-0113-7 PMC6862721

[77] Szlachta K, Kuscu C, Tufan T, Adair SJ, Shang S, Michaels AD, et al. CRISPR knockout screening identifies combinatorial drug targets in pancreatic cancer and models cellular drug response. Nat Commun. 2018;9:4275. 30323222 10.1038/s41467-018-06676-2 PMC6189038

[78] DepMap B. This DepMap release contains data from CRISPR knockout screens from project Achilles, as well as genomic characterization data from the CCLE project. For more information, please see README.txt. figshare. 2023 [cited 2023 Dec 26]. p. 19462523936 Bytes. Available from: https://figshare.com/articles/dataset/DepMap_23Q2_Public/22765112/2.

[79] Schneider CA, Rasband WS, Eliceiri KW. NIH Image to ImageJ: 25 years of image analysis. Nat Methods. 2012;9:671–5. 22930834 10.1038/nmeth.2089 PMC5554542

[80] Kurden-Pekmezci A, Cakiroglu E, Eris S, Mazi FA, Coskun-Deniz OS, Dalgic E, et al. MALT1 paracaspase is overexpressed in hepatocellular carcinoma and promotes cancer cell survival and growth. Life Sci. 2023;323. 121690. 37059355 10.1016/j.lfs.2023.121690

[81] Shi H, Rath EM, Lin RCY, Sarun KH, Clarke CJ, McCaughan BC, et al. 3-Dimensional mesothelioma spheroids provide closer to natural pathophysiological tumor microenvironment for drug response studies. Front Oncol. 2022;12:973576. 36091141 10.3389/fonc.2022.973576 PMC9462830

[82] Meyerholz DK, Beck AP. Principles and approaches for reproducible scoring of tissue stains in research. Lab Invest. 2018;98:844–55. 29849125 10.1038/s41374-018-0057-0

[83] Uchida KSK, Jo M, Nagasaka K, Takahashi M, Shindo N, Shibata K, et al. Kinetochore stretching-mediated rapid silencing of the spindle-assembly checkpoint required for failsafe chromosome segregation. Curr Biol. 2021;31:1581–91.e3. 33651990 10.1016/j.cub.2021.01.062

[84] Edgar R, Domrachev M, Lash AE. Gene Expression Omnibus: NCBI gene expression and hybridization array data repository. Nucleic Acids Res. 2002;30:207–10. 11752295 10.1093/nar/30.1.207 PMC99122

[85] Ghandi M, Huang FW, Jané-Valbuena J, Kryukov GV, Lo CC, McDonald ER, et al. Next-generation characterization of the cancer cell line encyclopedia. Nature. 2019;569:503–8. 31068700 10.1038/s41586-019-1186-3 PMC6697103

[86] Subramanian A, Tamayo P, Mootha VK, Mukherjee S, Ebert BL, Gillette MA, et al. Gene set enrichment analysis: a knowledge-based approach for interpreting genome-wide expression profiles. Proc Natl Acad Sci USA. 2005;102:15545–50. 16199517 10.1073/pnas.0506580102 PMC1239896

[87] Robin X, Turck N, Hainard A, Tiberti N, Lisacek F, Sanchez JC, et al. pROC: an open-source package for R and S+ to analyze and compare ROC curves. BMC Bioinforma. 2011;12:77. 10.1186/1471-2105-12-77 PMC3068975 21414208

[88] Hart T, Brown KR, Sircoulomb F, Rottapel R, Moffat J. Measuring error rates in genomic perturbation screens: gold standards for human functional genomics. Mol Syst Biol. 2014;10:733. 24987113 10.15252/msb.20145216 PMC4299491

