# Supplementary material for: Genome-wide CRISPR screen identifies BUB1 kinase as a druggable vulnerability in malignant pleural mesothelioma
Source: Cell Death Dis. 2025 Apr 3;16(1):241. doi: 10.1038/s41419-025-07587-z (PMC11968822; doi:10.1038/s41419-025-07587-z)
Supplement: Supplementary file 1 — Supplementary Figure Legends [file 41419_2025_7587_MOESM1_ESM.docx]

**Supplementary Figures**

**Supplementary Figure 1. Characterization of monoclonal Cas9 expression and activity in MPM cell lines. a** Cas9 expression of infected cells (Cas9) at the protein level compared to parental cells (Control). Flag-M2 antibody was used to measure Cas9 expression and β-actin was used as a loading control. **b** EGFP levels of Cas9 expressing MeT-5A, H2052, H2452, and H28 single cell clones (n=11-12). EGFP and Cas9 are co-expressed on the same mRNA via the P2A self-cleaving peptide, thus Cas9 expression is expected to be at a similar rate as EGFP expression. Selection of cells with high EGFP expression was considered to be advantageous for Cas9 expression and gene editing activity. **c** Western blot results of Cas9 protein levels tagged with Flag in five clones with higher GFP expression for each cell line. α-tubulin served as a loading control. **d** Competitive cell proliferation assay results of Cas9 expressing MeT-5A (Clones 2 and 3 ceases proliferation, thus Clone 5 was selected), H2052 (Clone 8), H2452 (Clone 4), and H28 (Clone 4) single cell clones infected with gRNAs targeting Renilla luciferase or human *RPA3* genes. The percentage of tomato-positive cells at Day 0 was normalized to 100%, and the following measurements were calculated accordingly. **e** Agarose gel images of *EGFR* amplicons digested with T7EI enzyme. While a ~1000 bp intact band was detected in gRen cells, a ~1000 bp intact band, as well as ~350 bp and ~650 bp bands were detected in cells targeted with EGFR gRNA consistent with mismatches resulting from indels. **f** The Lorenz curve drawn for the original library (orange) and the amplified library (blue). The AUC value was calculated as 0.66 for both libraries, indicating completely overlapping results.

**Supplementary Figure 2. Quality assessment of genome-wide CRISPR screens. a, b,** and **c** Distribution of mapped and unmapped gRNAs (**a**), missed gRNAs (**b**), and the Gini index of gRNAs (**c**) of each CRISPR screening group in Cas9 expressing MeT-5A, H2052, H2452, and H28 single cell clones. T0: time zero, G1-2-3: triplicates of T14, G: group. **d** Distribution of gRNAs targeting essential genes (orange dots) according to CRISPR viability score. **e** DAVID annotations showing the molecular functions of the top 200 genes with the highest depletion score in each cell line.

**Supplementary Figure 3. Functional characterization of high-confidence hits from CRISPR screening. a** Dot plots indicate CRISPR viability scores of seven genes whose depletion show non-discriminatory nature across all four cell lines and have known fitness functions in fundamental cellular processes. **b** List of genes that score in common in three MPM cell lines with minimal effect in MeT-5A cell line. Their functions are retrieved from GeneCards database. **c** CRISPR viability scores of genes that show selective negative enrichment in MPM cells. **d** gRNA scores of *AURKA*, *CDK2*, and *VPS37A* genes for MeT-5A, H2052, H2452, and H28 cells. X-axis indicates cell lines and the y-axis represents log2FC (fold change) for each gRNA. **e** Western blot assessment of AURKA, CDK2, and VPS37A knockout in MeT-5A, H2052, H2452, and H28 cells. Cells were infected with lentiCRISPR v2 gRen (control) or lentiCRISPR v2 AURKA (g1, g2), CDK2 (g1, g4), and VPS37A (g1, g4) lentiviral vectors and selected with puromycin for 3 days. Protein levels were detected at 6 days post infection. β-actin served as the equal loading control. **f** and **g** CRISPR gene effect scores (**f**) and expression levels (**g**) of *AURKA*, *CDK2*, and *VPS37A* genes across all MPM cell lines in the DepMap database. The MPM cell lines used in this study are highlighted with red borders.

**Supplementary Figure 4. Functional insights into the role of BUB1 in MPM cells. a** Interaction partners and substrates of BUB1 annotated in the StringDB protein interaction database and their CRISPR viability scores obtained from CRISPR screens. **b** Crystal structure of the human BUB1 kinase in complex with BAY-1816032, as retrieved from CanSar and visualized with PDB (6F7B, https://www.rcsb.org/). Yellow color indicates tyrosine kinase domain and red color represents BAY-1816032. **c** Correlation analysis of expression between BUB1 and proliferation markers *CCNB1* and *CDC20* in the TCGA MPM dataset. Log2 transformed mRNA levels were compared through cBioPortal. Pearson correlation coefficient (r) for CCNB1 is 0.81 (p-value = 3.20e-21), and for CDC20, it is 0.85 (p-value = 3.34e-25). **d** and **e** CRISPR gene effect scores (**d**) and expression levels (**e**) of *BUB1* gene across all MPM cell lines in DepMap database. The MPM cell lines used in this study are highlighted with red borders. **f** BUB1 protein levels in H2052, H2452 and H28 cells with or without BUB1 depletion forced to express gRNA resistant cDNA-encoded wildtype human BUB1 protein (mBUB1: mutant for gRNA sequence). Cells were infected with lentiCRISPR v2 gRen (control) or lentiCRISPR v2 BUB1 g2 lentiviral vectors and selected with puromycin for 3 days. BUB1 levels were detected at 6 days post infection. β-actin served as the equal loading control. **g** Representative images showing restored 2D colony formation capacity of H2052, H2452 and H28 cells with or without BUB1 depletion, forced to express gRNA resistant BUB1-cDNA. Colony formation assay was performed in triplicates in 12-well cell culture plates for 10-14 days. High-resolution images of the plates were acquired by LI-COR Odyssey CLx Imaging System. **h** Crystal violet intensity data showing the relative difference in 2D colony forming capacity of each group. Image Studio software was used to measure signal intensities. Bar graphs are presented as the mean ± SD of three replicates. Two-tailed Student’s t-test was used for statistical analysis. Asterisks above the bars indicate significance compared to the Vector+gRen group. *p<0.05, **p<0.01 ***p<0.001, ns: not significant.

**Supplementary Figure 5. Transcriptomic landscapes of BUB1-depleted MPM cells. a** PCA plot elucidating variance (%) in transcriptome data of BUB1 WT vs BUB1 KO MPM cell lines. **b** Volcano plot depicting the differential gene expression profiles between BUB1 WT vs BUB1 KO MPM cell lines. A threshold of *p-value* < 0.05 was applied for all cell lines, with |logFC| ≥ 1 for H2052 and H2452, and |logFC| ≥ 0.8 for the H28 cell line. **c** Bar plot showing the number of differentially expressed genes. Blue denotes upregulated genes, while orange indicates downregulated genes in BUB1 KO MPM cell lines. **d** Gene set enrichment analysis (GSEA) visualized with BubbleGUM, illustrating pathway enrichment for both enriched and depleted gene signatures in BUB1 KO MPM cell lines. Circle area indicates NES, and color intensity represents FDR. NS, not significant. **e** GSEA plots from H28 cell line. The plots indicate significantly depleted E2F, MYC, and G2/M checkpoint target gene expression, all reflecting repression of cell proliferation in MPM cell lines with BUB1 knockout (BUB1 KO). The y-axis represents the enrichment score (ES). The significance of correlation is depicted on the x-axis, by the red color for positive and the blue color for negative correlation. Normalized enrichment score (NES), false discovery rate (FDR) and p-values are shown. **f** (left panel) Representative images of SA-β-Gal staining (blue) assay identifying increased senescence in BUB1 depleted monoclonal cells compared to WT cells. Scale bar: 100 µm. (right panel) Percentage of SA-β-Gal staining. All cell populations as well as SA-β-Gal stained cells were counted and the percentage of SA-β-Gal staining was calculated by proportioning the number of stained cells to the total number of cells. Data are presented as mean ± SD, n=4. Two-tailed Student’s t-test was used for statistical analysis. ***p<0.001. **g** qRT-PCR analysis of BUB1 depleted cells (BUB1 KO), as compared to BUB1 WT control cells, showed increased expression of IL6, IL1⍺ and IL1β. The expression levels were calculated by normalizing to the BUB1 WT group, which was set to 1 and shown as the mean ± SD of three replicates.

**Supplementary Figure 6. Enrichment of proliferation pathways in MPM tumors with high BUB1 expression.** **a,b,** and **c** GSEA plots from GSE2549, GSE29211, GSE42977 and GSE163722 datasets. The plots show significantly enriched E2F (**a**), MYC (**b**), and G2/M checkpoint (**c**) target gene expression, all reflecting activation of cell proliferation in MPM tumors with high BUB1 expression (BUB1 High). The y-axis represents the enrichment score (ES). The significance of correlation is depicted on the x-axis, by the red color for positive and the blue color for negative correlation. Normalized enrichment score (NES), false discovery rate (FDR) and p-values are shown.

**Supplementary Figure 7. Enrichment of EMT phenotype in MPM tumors with high BUB1 expression.** GSEA plots from GSE2549, GSE29211 and GSE163722 datasets. The plots show significantly enriched EMT target gene expression in MPM tumors with high *BUB1* expression (BUB1 High). The y-axis represents the enrichment score (ES). The significance of correlation is depicted on the x-axis, by the red color for positive and the blue color for negative correlation. Normalized enrichment score (NES), false discovery rate (FDR) and p-values are shown.

**Supplementary Figure 8. Immunohistochemical evaluation of tissue samples. a** Representative images (100X magnification) depicting different IRS (ranging from 0 to 6) of normal and MPM tissues shown in Fig. 6C. Scale bar: 10 μm. **b** Representative images (40X magnification) showing different IRS (ranging from 3 to 9) of malignant mesothelioma (MM) tissues shown in Fig. 6D. Scale bar: 20 μm.

**Supplementary Figure 9. Transcriptomic analysis of BUB1-associated genes in MPM cells. a** StringDB analysis of BUB1 local network genes annotated in KEGG_Cell_Cycle Pathway (hsa04110). **b** Heatmap depicting the expression levels of BUB1 local network genes (highlighted in Fig. S9A) in BUB1 knockout (BUB1 KO) H28 cells. **c** BUB1, Cyclin B, Cyclin A, CDC20, and p21 protein levels in BUB1 WT vs BUB1 KO H28 cells. β-actin was used as a loading control.

**Supplementary Figure 10. Effect of BAY-1816032 treatment on cancer cell phenotypes. a** Representative images showing the impact of pharmacological BUB1 inhibition on spheroid growth. MPM cells were grown in 96 well ultra-low attachment plates to form spheroids and treated with BAY-1816032 for 7 days. Scale bar: 400 µm. **b** Bar graphs of the apoptosis assay revealing a slight increase in apoptosis in BAY-1816032 treated cells. Percentage of apoptotic cells was calculated as the sum of early and late apoptosis. Data are presented as mean ± SD of two biological replicates. Two-tailed Student’s t-test was used for statistical analysis. *p<0.05, ns: not significant.
