# Supplementary material for: Genome-wide CRISPR screen identifies BUB1 kinase as a druggable vulnerability in malignant pleural mesothelioma
Source: Cell Death Dis. 2025 Apr 3;16(1):241. doi: 10.1038/s41419-025-07587-z (PMC11968822; doi:10.1038/s41419-025-07587-z)
Supplement: Supplementary file 2 — Supplementary Tables [file 41419_2025_7587_MOESM2_ESM.docx]

**Supplementary Tables**

**Supplementary Table 1.** Primer sequences used for gRNA cloning.

| gRNA | Forward primer (5’-3’) | Reverse primer (5’-3’) |
| --- | --- | --- |
| Renilla (Ren) | CACCGGTAGCGCGGTGTATTATACC | AAACGGTATAATACACCGCGCTACC |
| EGFR | CACCGTCCTCCAGAGCCCGACTCGC | AAACGCGAGTCGGGCTCTGGAGGAC |
| RPA3 | CACCGGATGAATTGAGCTAGCATGC | AAACGCATGCTAGCTCAATTCATCC |
| AURKA (g1) | CACCGCCATATAGAAAATAATCCTG | AAACCAGGATTATTTTCTATATGGC |
| AURKA (g4) | CACCGCCTGAAAACTCACCGAAGGT | AAACACCTTCGGTGAGTTTTCAGGC |
| CDK2 (g1) | CACCGAAGCAGAGAGATCTCTCGGA | AAACTCCGAGAGATCTCTCTGCTTC |
| CDK2 (g2) | CACCGCAAATATTATTCCACAGCTG | AAACCAGCTGTGGAATAATATTTGC |
| VPS37A (g1) | CACCGAGCCGAAATACAGAAAGATG | AAACCATCTTTCTGTATTTCGGCTC |
| VPS37A (g4) | CACCGGGTGGATAAACACTGATCAC | AAACGTGATCAGTGTTTATCCACCC |
| BUB1 (g1) | CACCGCAAGGAGAAGCTTATTCGTG | AAACCACGAATAAGCTTCTCCTTGC |
| BUB1 (g2) | CACCGTCAGAGAGGAATTCAAAACC | AAACGGTTTTGAATTCCTCTCTGAC |
| BUB1 (g3) | CACCGCCCGCCCAGGCAATGTACAG | AAACCTGTACATTGCCTGGGCGGGC |
| BUB1 (g4) | CACCGAACAGACTCCATGTTTGCAG | AAACCTGCAAACATGGAGTCTGTTC |

**Supplementary Table 2.** Primer sequences used for gene amplification.

| Gene | Forward primer (5’-3’) | Reverse primer (5’-3’) |
| --- | --- | --- |
| EGFR | TTGGCTCGACCTGGACATAG | GGGGAAAGTGAGGGAAGAAA |
| BUB1 | TCGTGAGCGGCCGCCACCATGGACA | AGAGGGGCGGATCCTCTAGAACTAG |
| BUB1 g2_res | AGCATGCCAGTGCTGTCCTTCAACGTGGAATCCAGAACCAAGCTGAACCCAGAGAGTTCCT | AGGAACTCTCTGGGTTCAGCTTGGTTCTGGATTCCACGTTGAAGGACAGCACTGGCATGCT |

**Supplementary Table 3.** Primer sequences used in the amplification of sequence libraries.

| Primer | Sequence (5’-3’) |
| --- | --- |
| P5 0 nt stagger | AATGATACGGCGACCACCGAGATCTACACTCTTTCCCTACACGACGCTCTTCCGATCTTTGTGGAAAGGACGAAACACCG |
| P5 1 nt stagger | AATGATACGGCGACCACCGAGATCTACACTCTTTCCCTACACGACGCTCTTCCGATCTCTTGTGGAAAGGACGAAACACCG |
| P5 2 nt stagger | AATGATACGGCGACCACCGAGATCTACACTCTTTCCCTACACGACGCTCTTCCGATCTGCTTGTGGAAAGGACGAAACACCG |
| P5 3 nt stagger | AATGATACGGCGACCACCGAGATCTACACTCTTTCCCTACACGACGCTCTTCCGATCTAGCTTGTGGAAAGGACGAAACACCG |
| P5 4 nt stagger | AATGATACGGCGACCACCGAGATCTACACTCTTTCCCTACACGACGCTCTTCCGATCTCAACTTGTGGAAAGGACGAAACACCG |
| P5 6 nt stagger | AATGATACGGCGACCACCGAGATCTACACTCTTTCCCTACACGACGCTCTTCCGATCTTGCACCTTGTGGAAAGGACGAAACACCG |
| P5 7 nt stagger | AATGATACGGCGACCACCGAGATCTACACTCTTTCCCTACACGACGCTCTTCCGATCTACGCAACTTGTGGAAAGGACGAAACACCG |
| P5 8 nt stagger | AATGATACGGCGACCACCGAGATCTACACTCTTTCCCTACACGACGCTCTTCCGATCTGAAGACCCTTGTGGAAAGGACGAAACACCG |
| P7 index 1 | CAAGCAGAAGACGGCATACGAGATGCTGAGAAGTGACTGGAGTTCAGACGTGTGCTCTTCCGATCTTCTACTATTCTTTCCCCTGCACTGT |
| P7 index 2 | CAAGCAGAAGACGGCATACGAGATTAGTTCGGGTGACTGGAGTTCAGACGTGTGCTCTTCCGATCTTCTACTATTCTTTCCCCTGCACTGT |
| P7 index 3 | CAAGCAGAAGACGGCATACGAGATTAACTCGGGTGACTGGAGTTCAGACGTGTGCTCTTCCGATCTTCTACTATTCTTTCCCCTGCACTGT |
| P7 index 4 | CAAGCAGAAGACGGCATACGAGATTACAGAGGGTGACTGGAGTTCAGACGTGTGCTCTTCCGATCTTCTACTATTCTTTCCCCTGCACTGT |
| P7 index 5 | CAAGCAGAAGACGGCATACGAGATGCACGATTGTGACTGGAGTTCAGACGTGTGCTCTTCCGATCTTCTACTATTCTTTCCCCTGCACTGT |
| P7 index 6 | CAAGCAGAAGACGGCATACGAGATATTGGAGGGTGACTGGAGTTCAGACGTGTGCTCTTCCGATCTTCTACTATTCTTTCCCCTGCACTGT |
| P7 index 7 | CAAGCAGAAGACGGCATACGAGATATACTCGGGTGACTGGAGTTCAGACGTGTGCTCTTCCGATCTTCTACTATTCTTTCCCCTGCACTGT |
| P7 index 8 | CAAGCAGAAGACGGCATACGAGATCGGTTCGGGTGACTGGAGTTCAGACGTGTGCTCTTCCGATCTTCTACTATTCTTTCCCCTGCACTGT |

**Supplementary Table 4.** Primer sequences used for qRT-PCR.

| Gene | Forward primer (5’-3’) | Reverse primer (5’-3’) |
| --- | --- | --- |
| IL6 | AACCTGAACCTTCCAAAGATGG | TCTGGCTTGTTCCTCACTACT |
| IL1α | GAATGACGCCCTCAATCAAAGT | TCATCTTGGGCAGTCACATACA |
| IL1β | GGCCACATTTGGTTCTAAGAAA | TAAATAGGGAAGCGGTTGCTC |
