# Supplementary figures and images for: Genome-wide CRISPR screen identifies BUB1 kinase as a druggable vulnerability in malignant pleural mesothelioma
Source: Cell Death Dis. 2025 Apr 3;16(1):241. doi: 10.1038/s41419-025-07587-z (PMC11968822; doi:10.1038/s41419-025-07587-z)

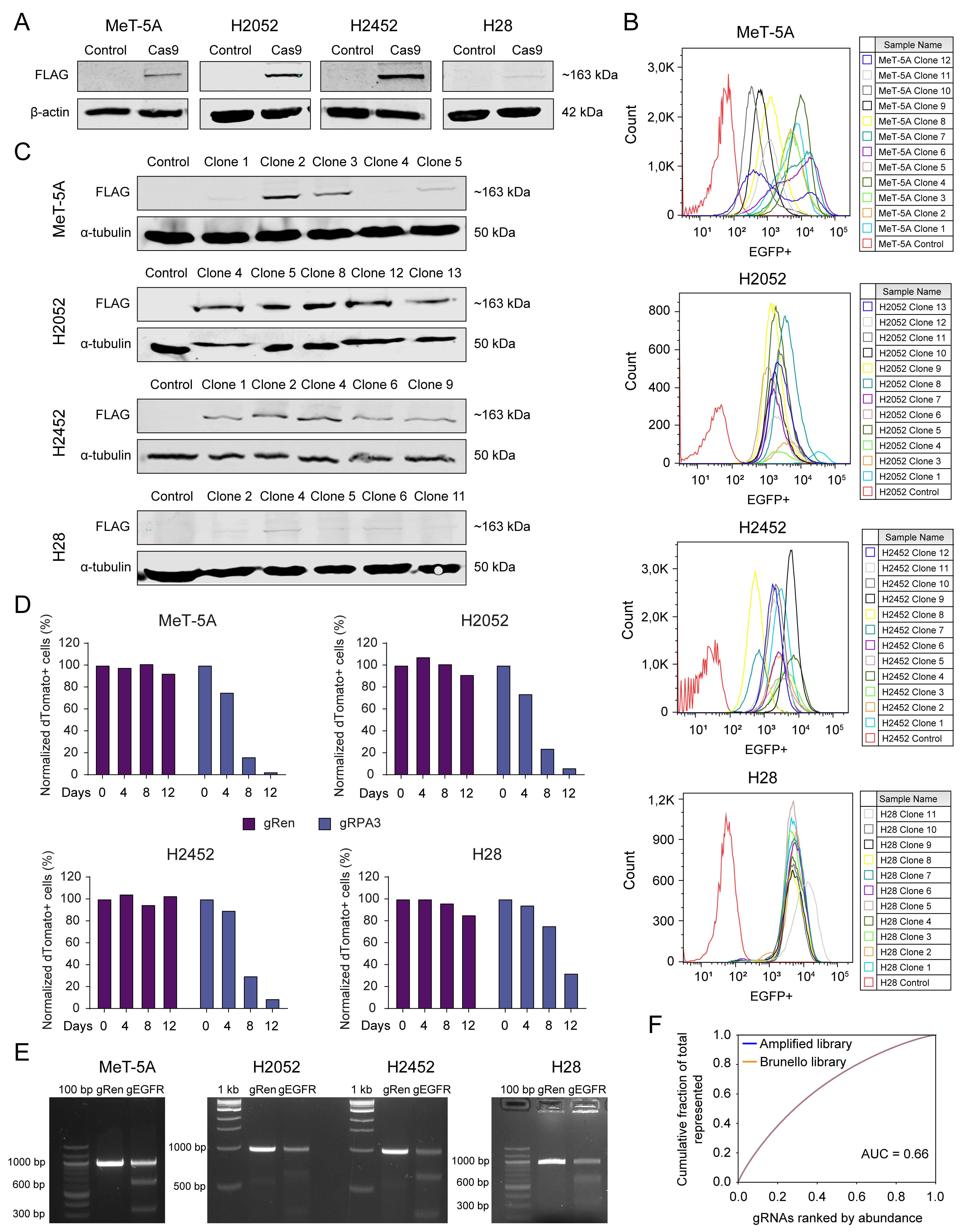

Supplement: Supplementary file 3 — Supplementary Figure 1 [file 41419_2025_7587_MOESM3_ESM.tif]

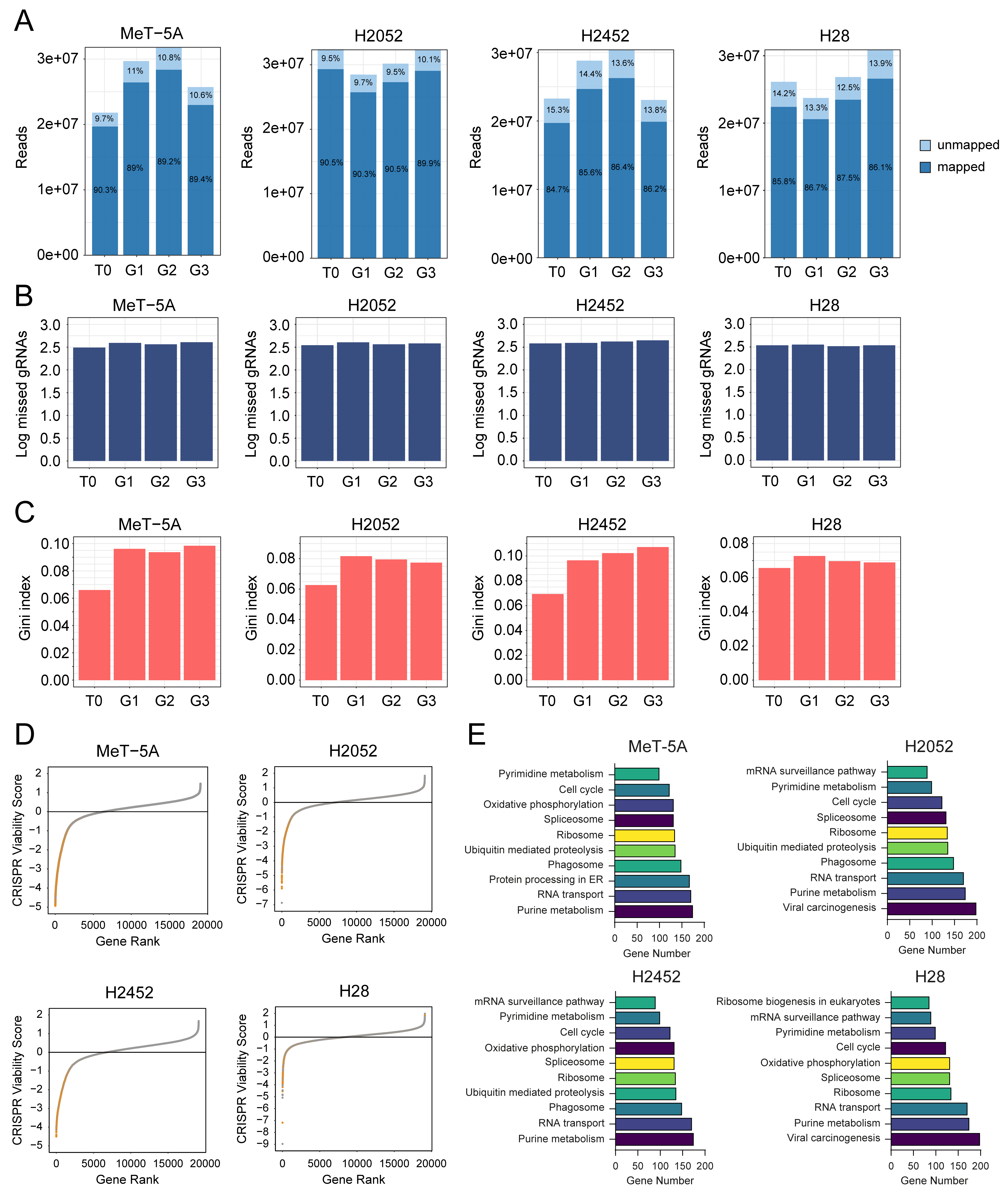

Supplement: Supplementary file 4 — Supplementary Figure 2 [file 41419_2025_7587_MOESM4_ESM.tif]

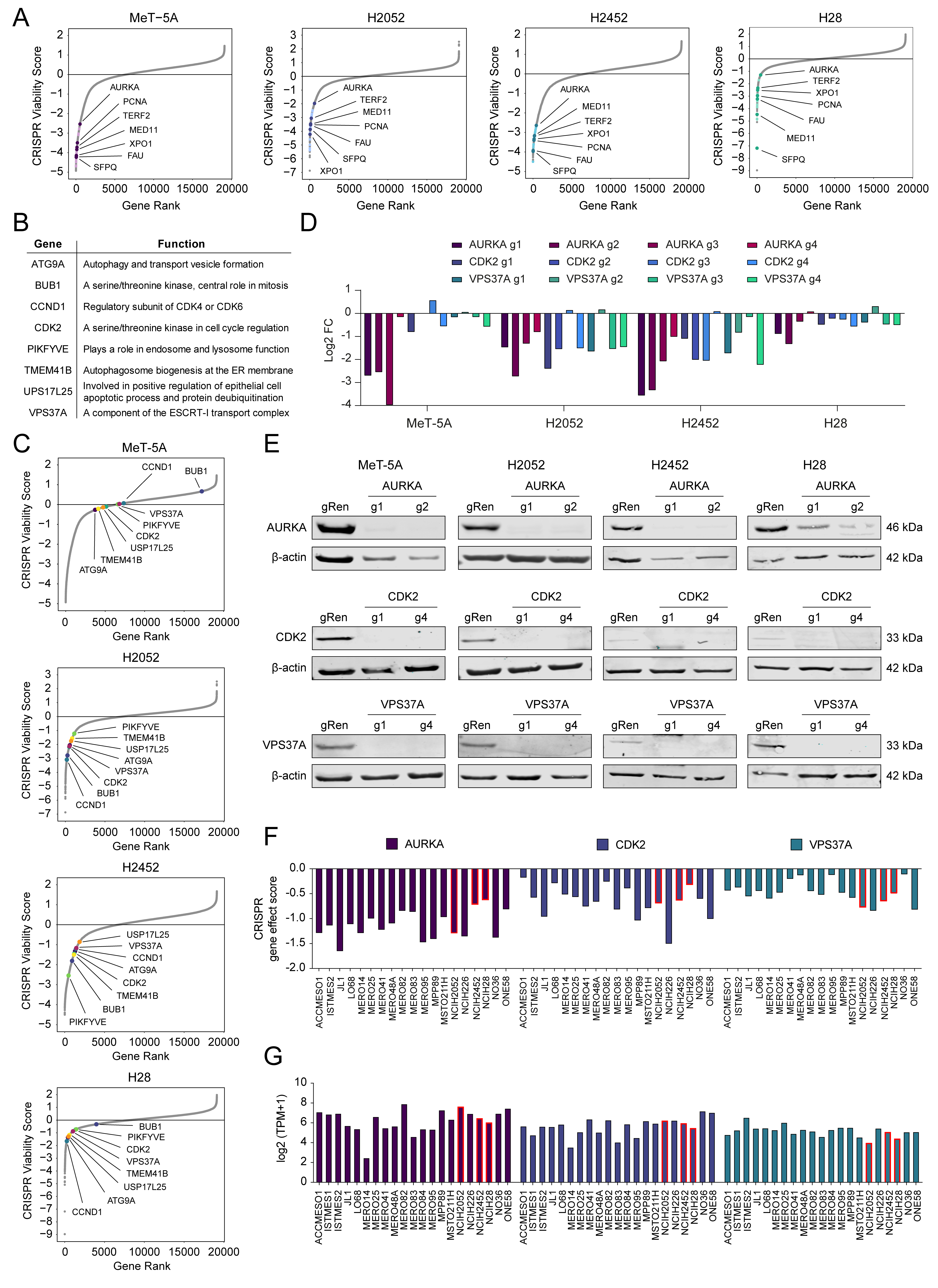

Supplement: Supplementary file 5 — Supplementary Figure 3 [file 41419_2025_7587_MOESM5_ESM.tif]

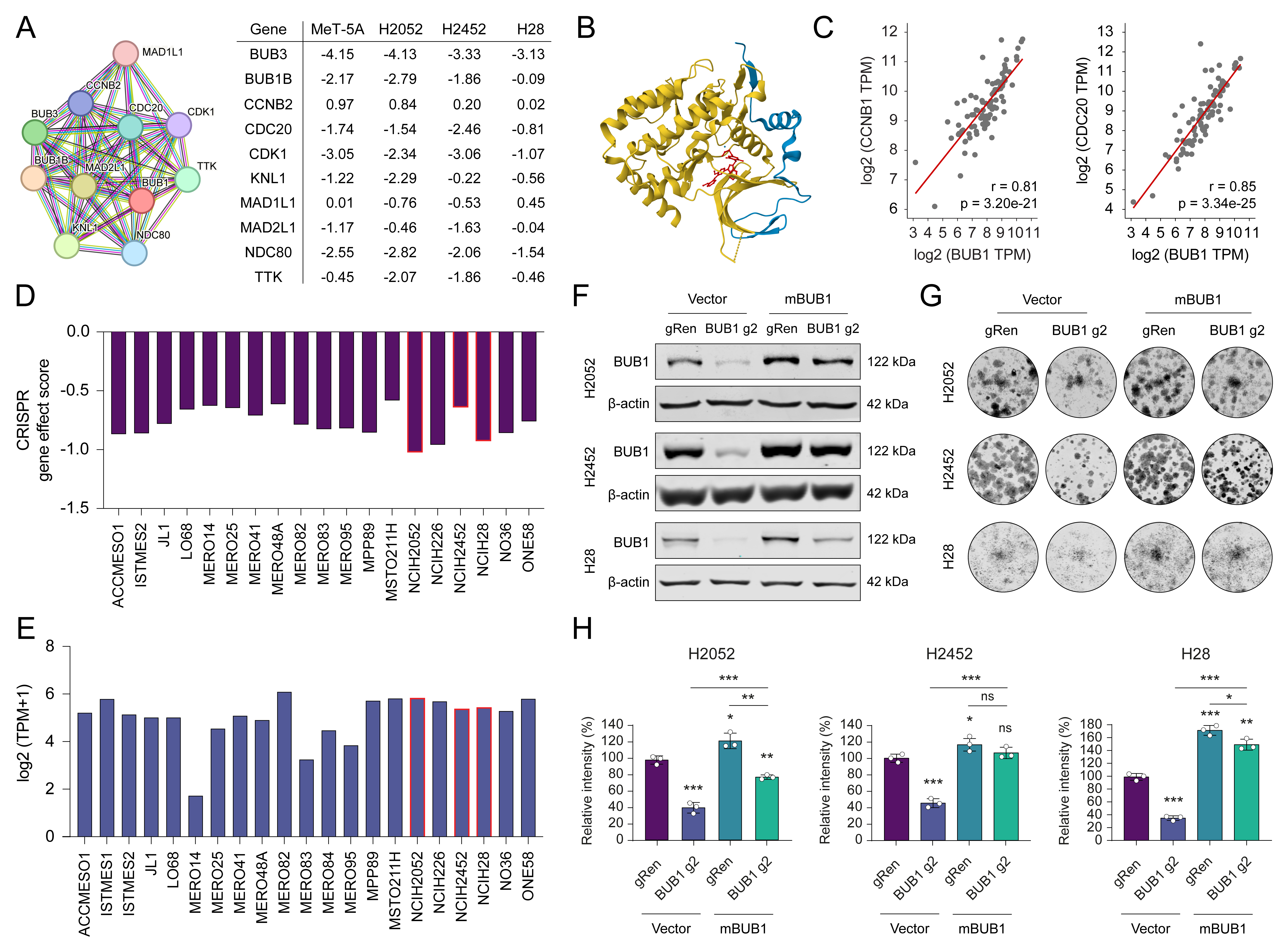

Supplement: Supplementary file 6 — Supplementary Figure 4 [file 41419_2025_7587_MOESM6_ESM.tif]

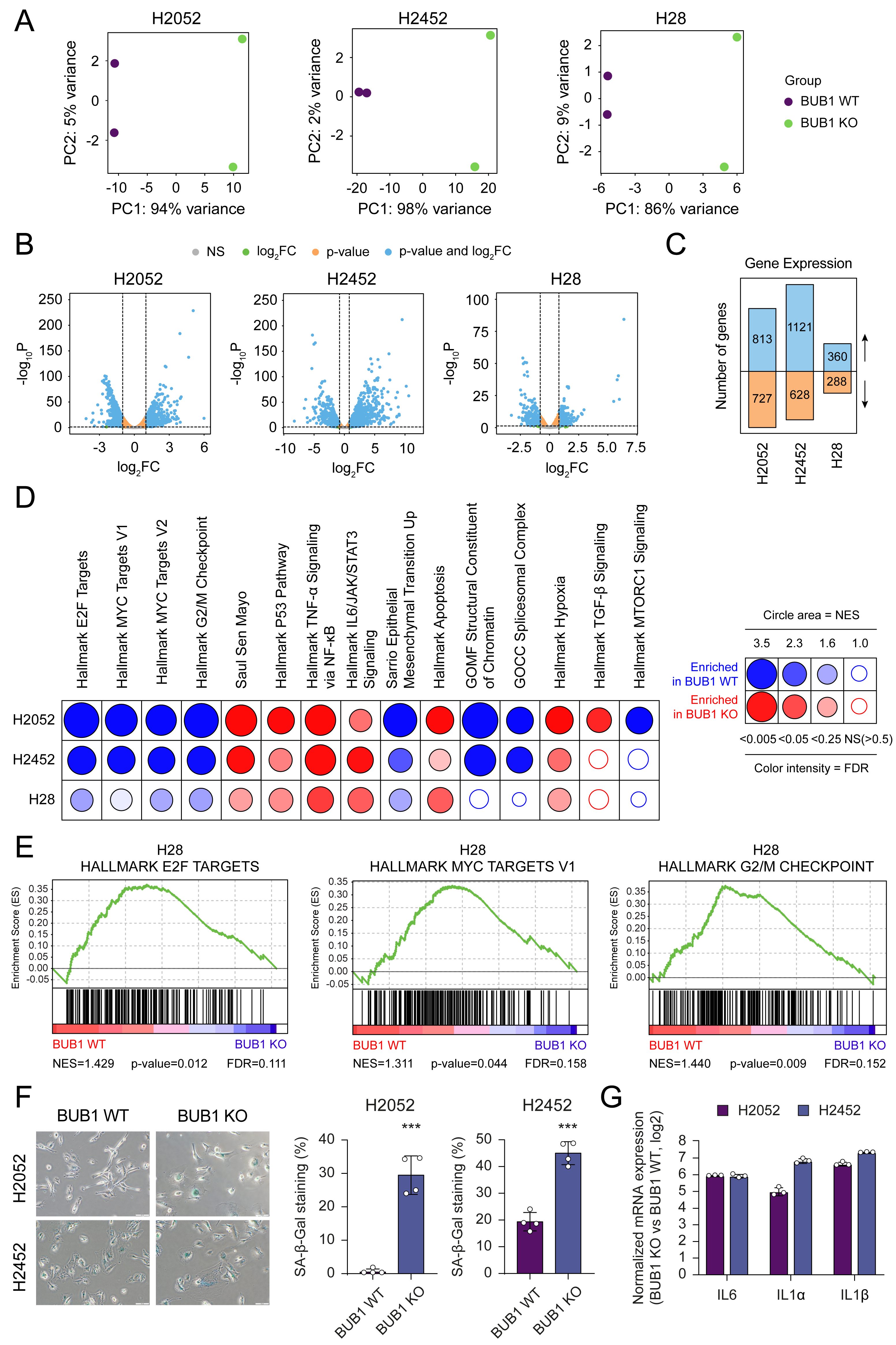

Supplement: Supplementary file 7 — Supplementary Figure 5 [file 41419_2025_7587_MOESM7_ESM.tif]

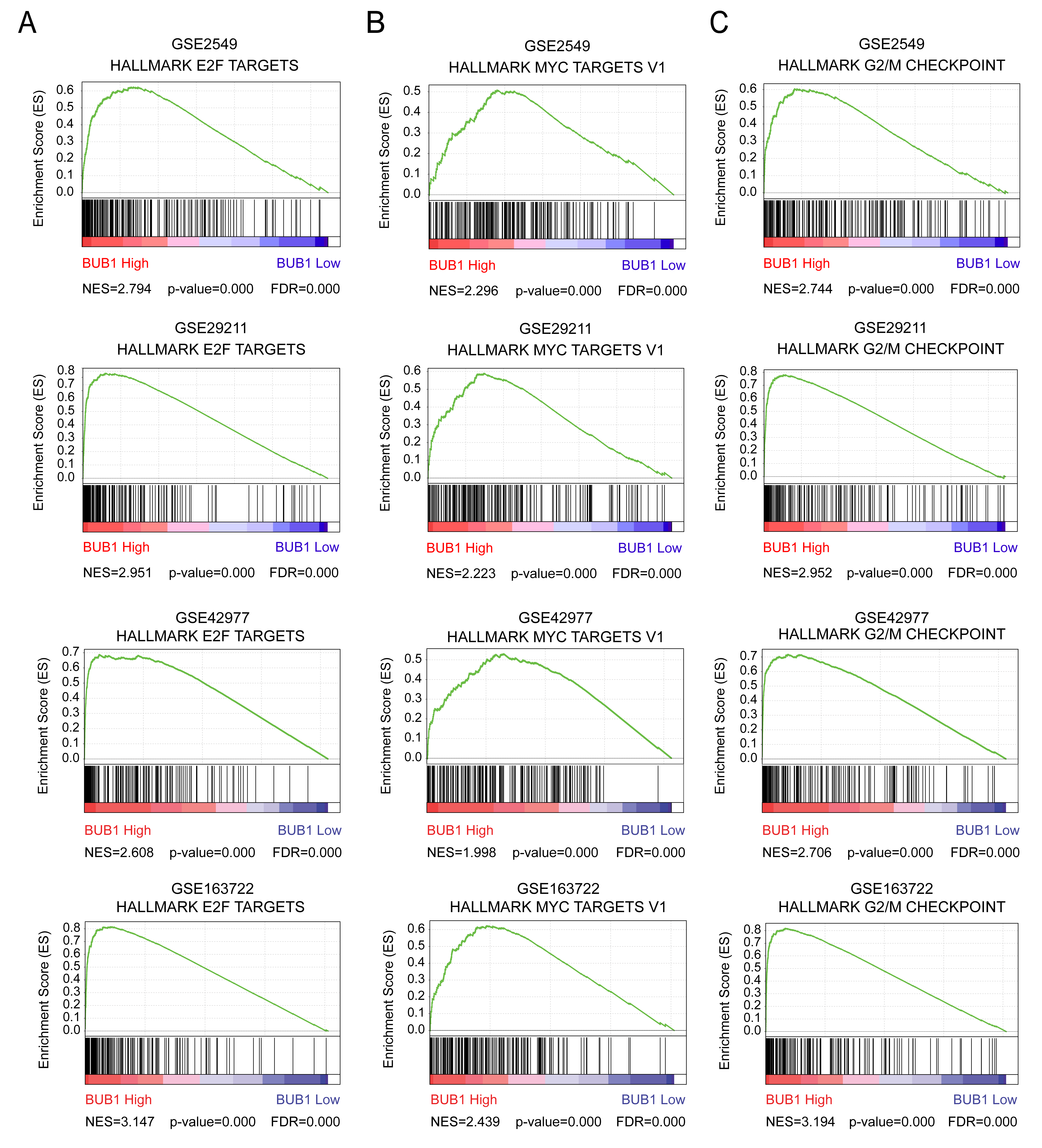

Supplement: Supplementary file 8 — Supplementary Figure 6 [file 41419_2025_7587_MOESM8_ESM.tif]

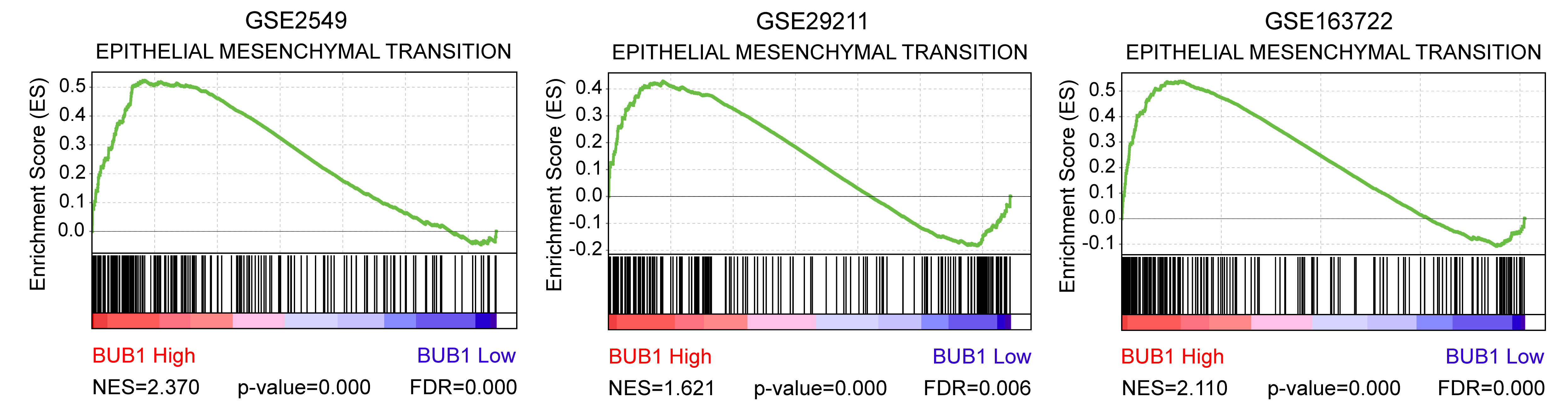

Supplement: Supplementary file 9 — Supplementary Figure 7 [file 41419_2025_7587_MOESM9_ESM.tif]

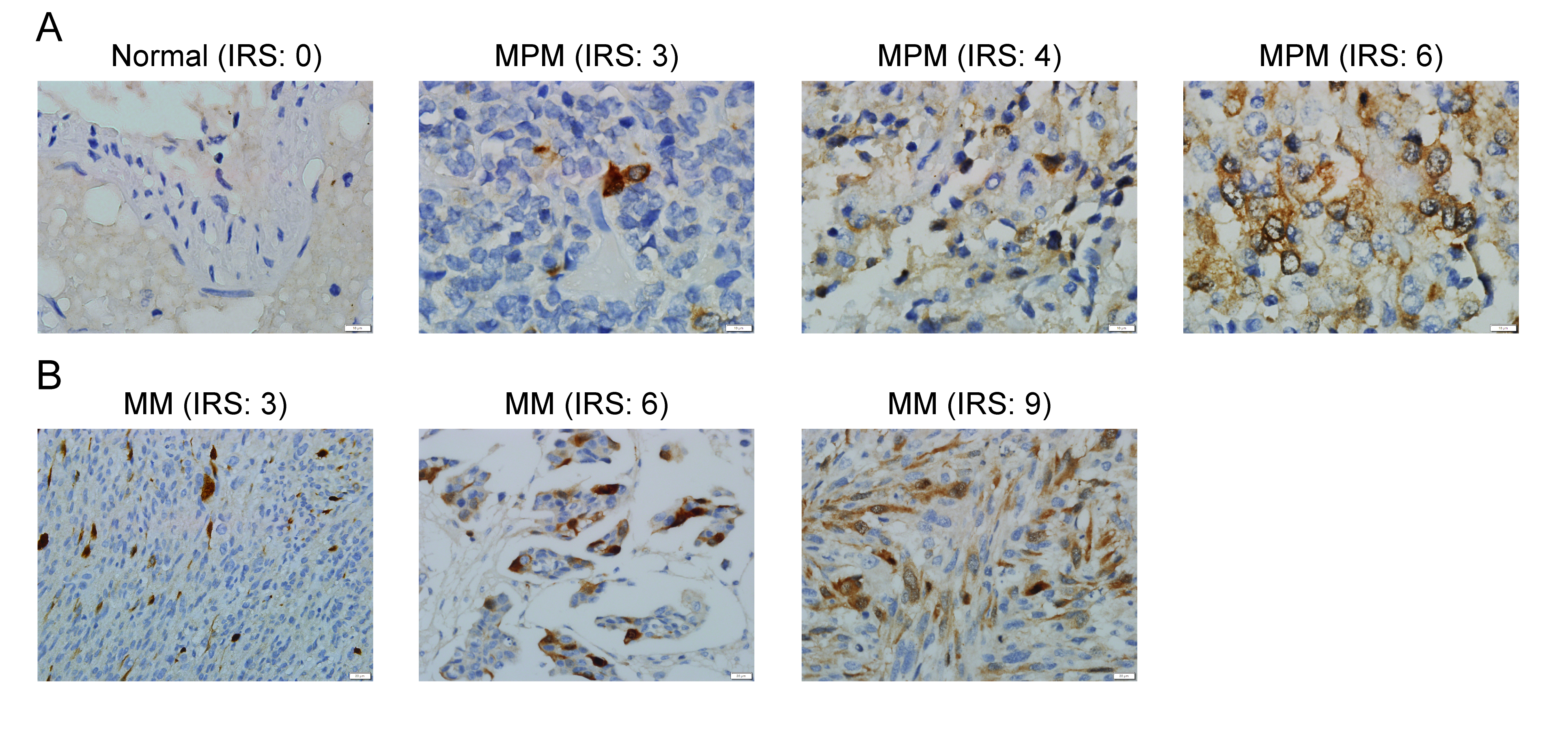

Supplement: Supplementary file 10 — Supplementary Figure 8 [file 41419_2025_7587_MOESM10_ESM.tif]

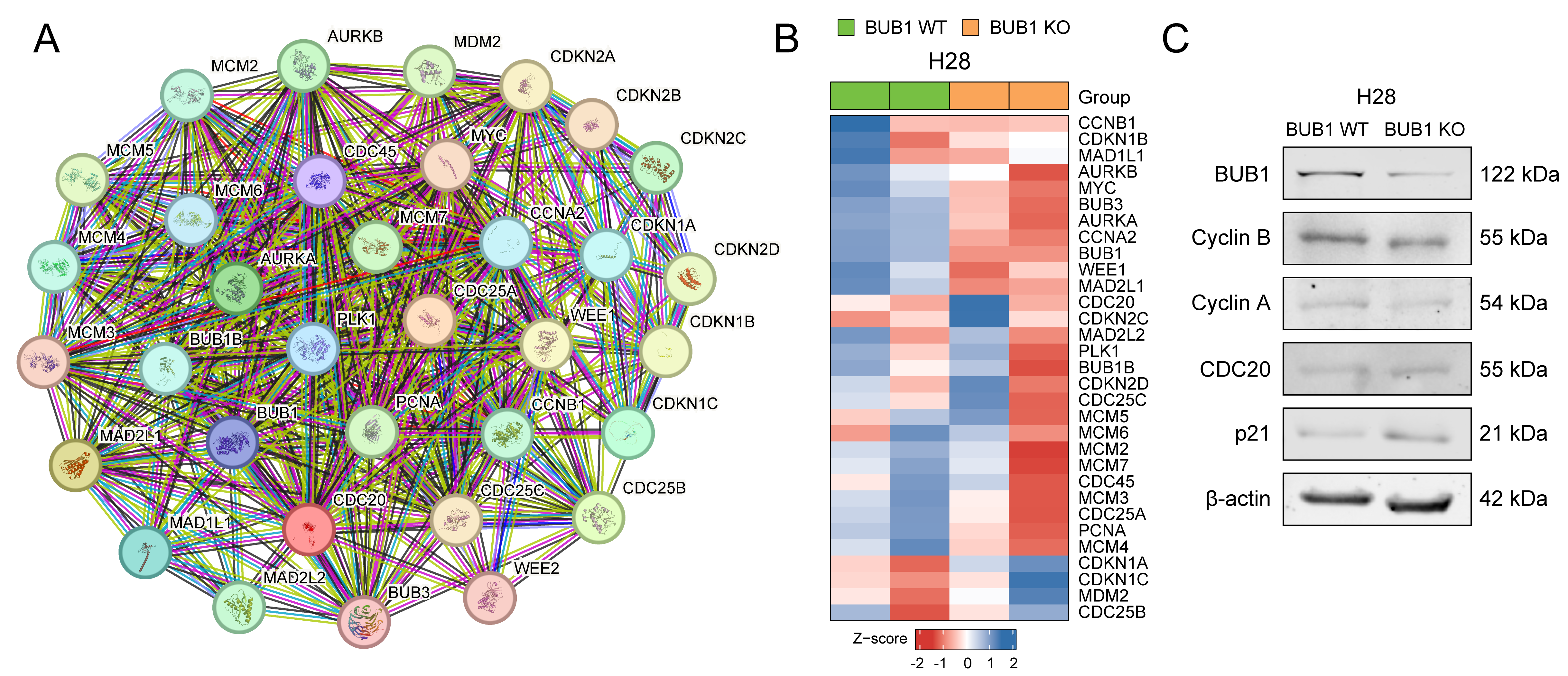

Supplement: Supplementary file 11 — Supplementary Figure 9 [file 41419_2025_7587_MOESM11_ESM.tif]

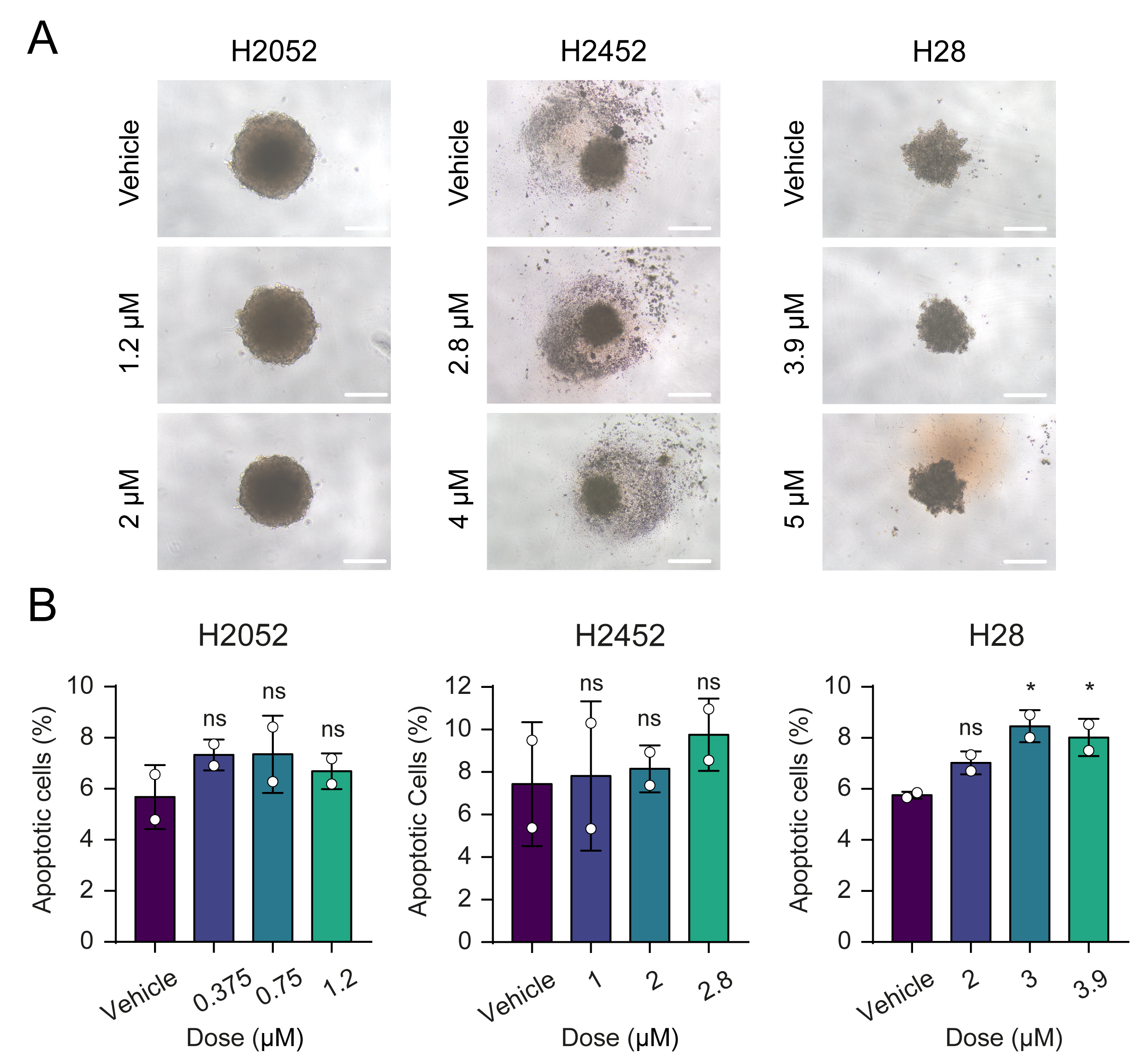

Supplement: Supplementary file 12 — Supplementary Figure 10 [file 41419_2025_7587_MOESM12_ESM.tif]

Fig. S1A

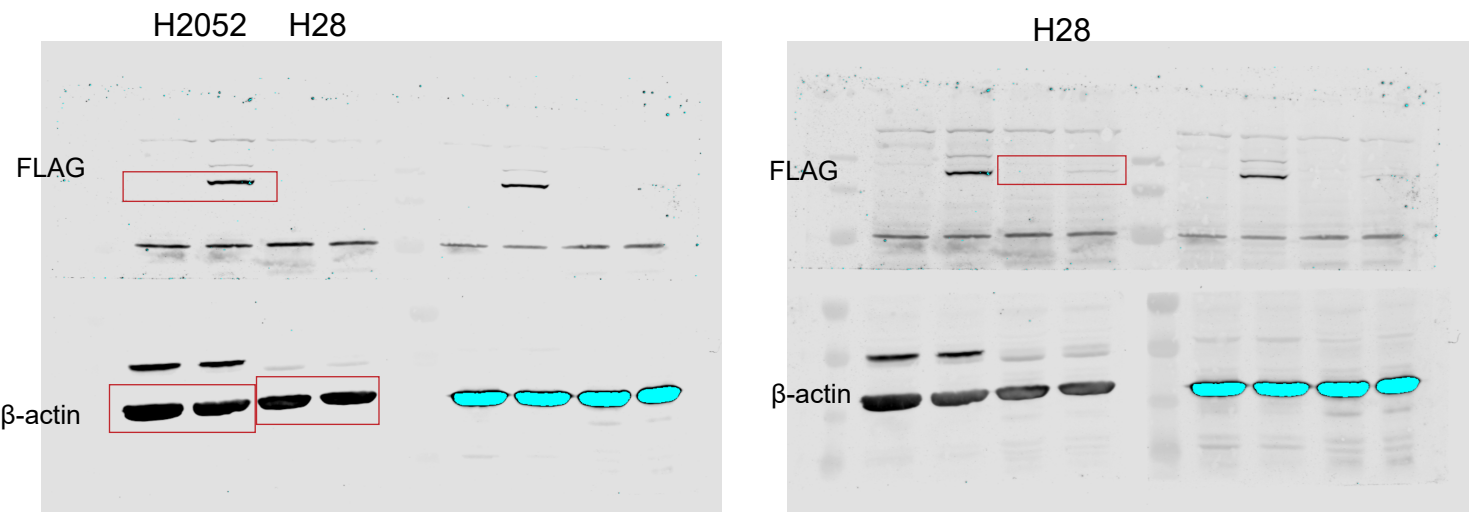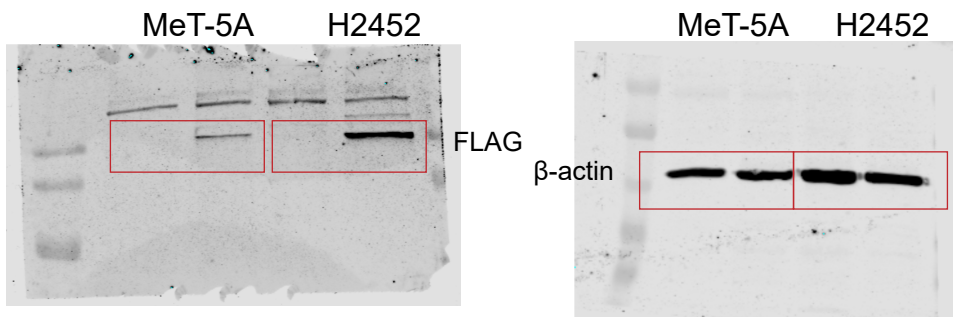

Fig. S1C

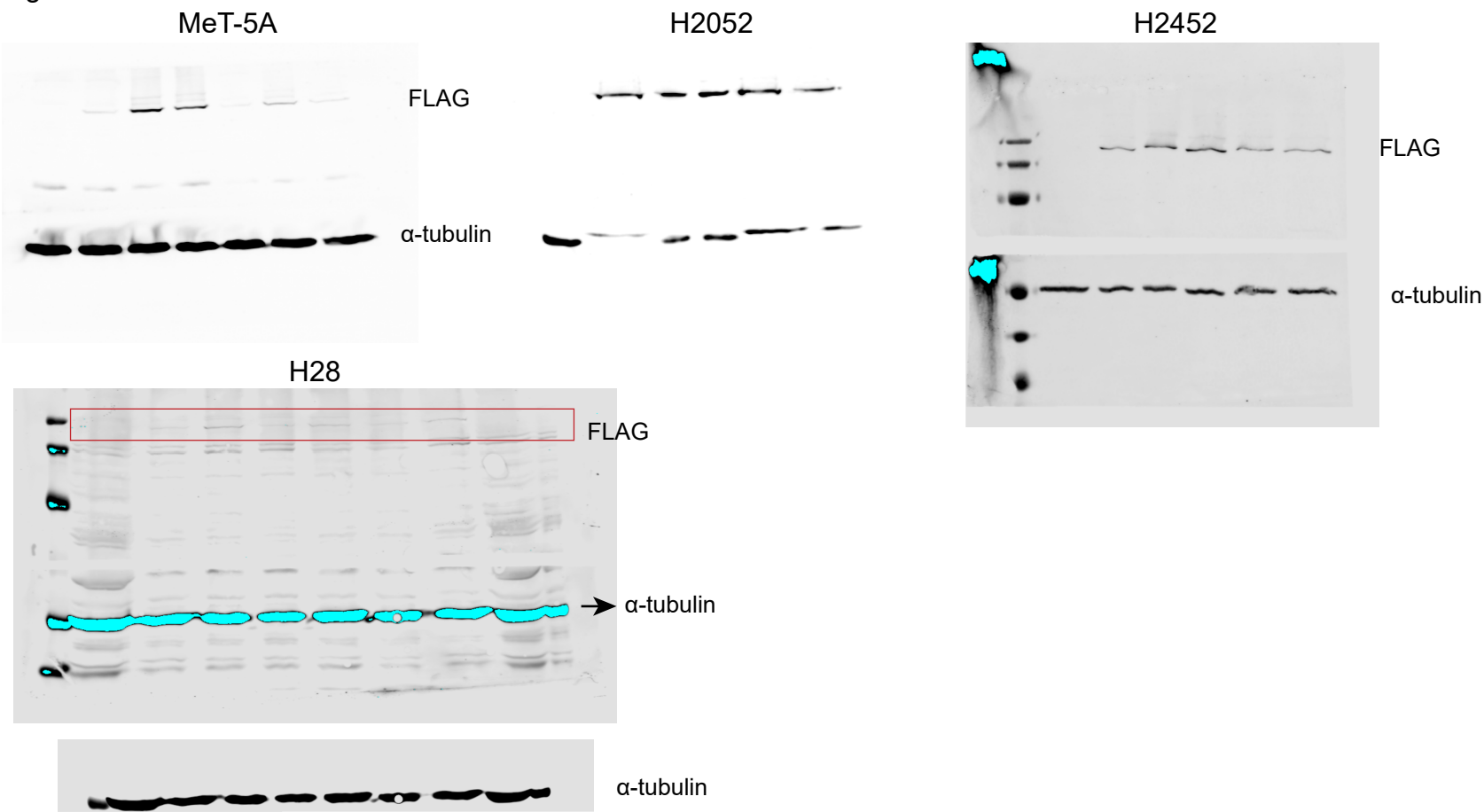

Fig. 3C

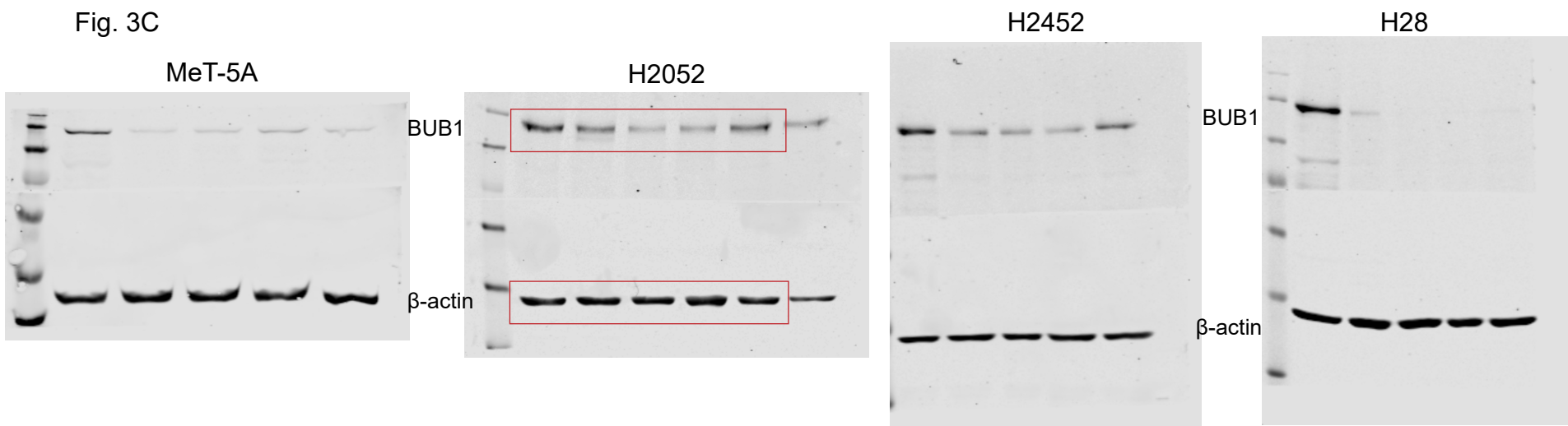

Fig. S4F

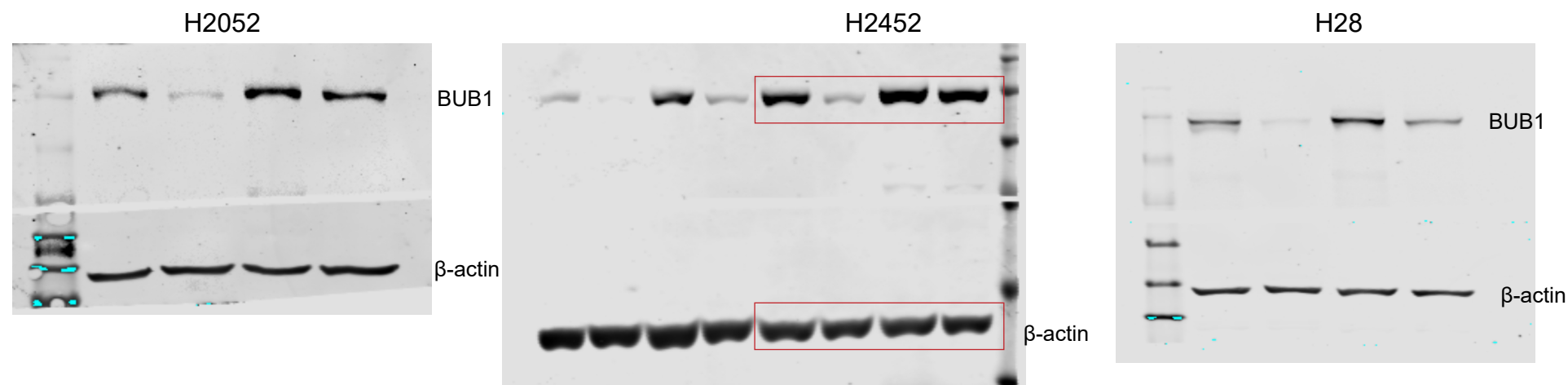

Fig. 7A

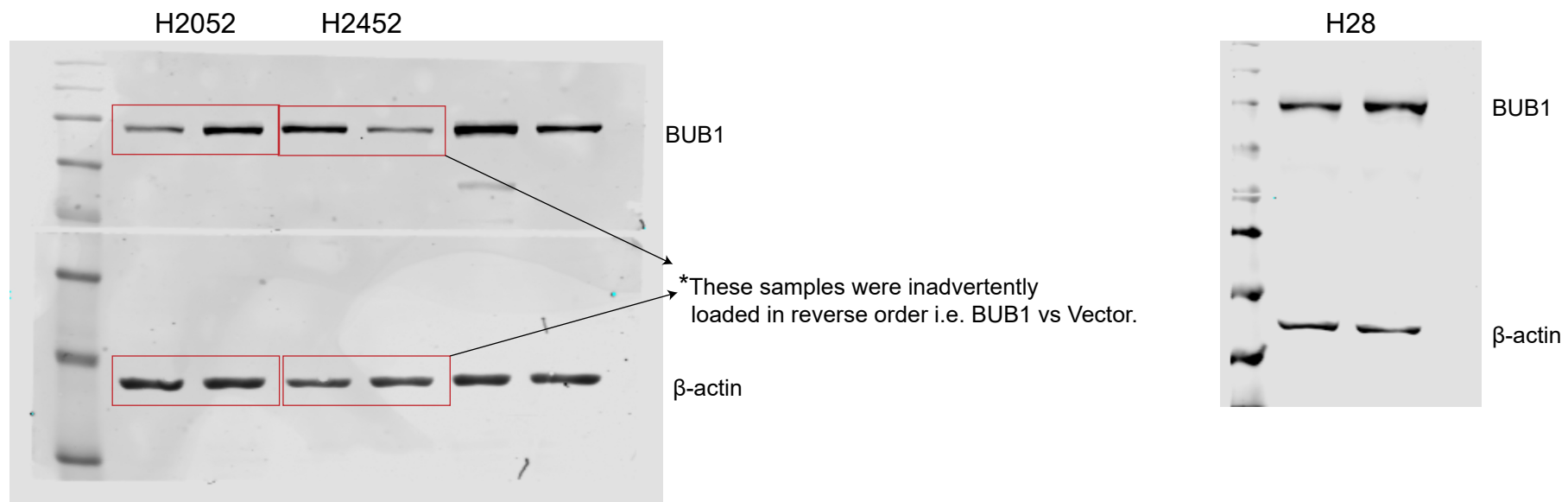

Fig. S3E

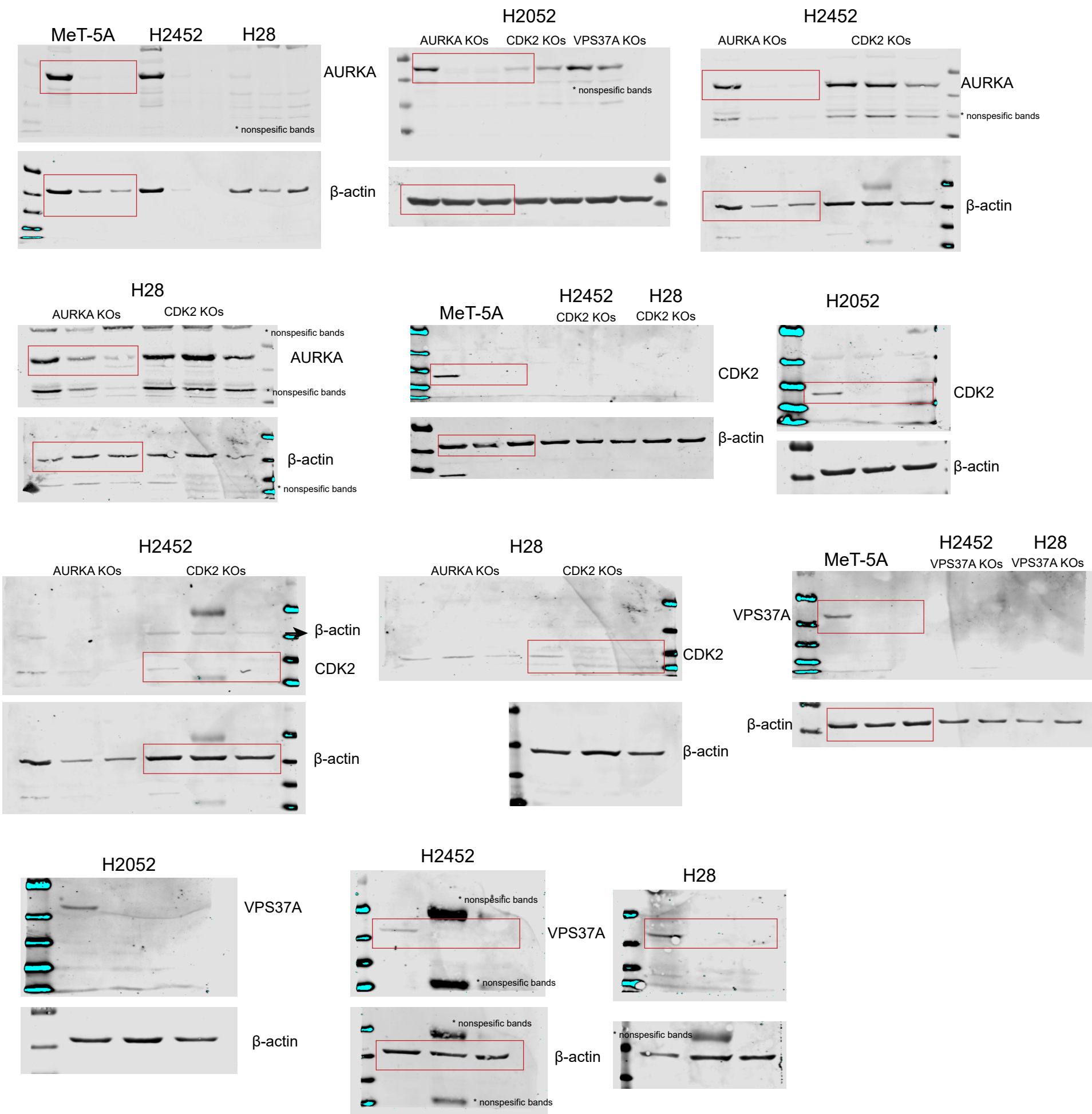

Fig. 8B

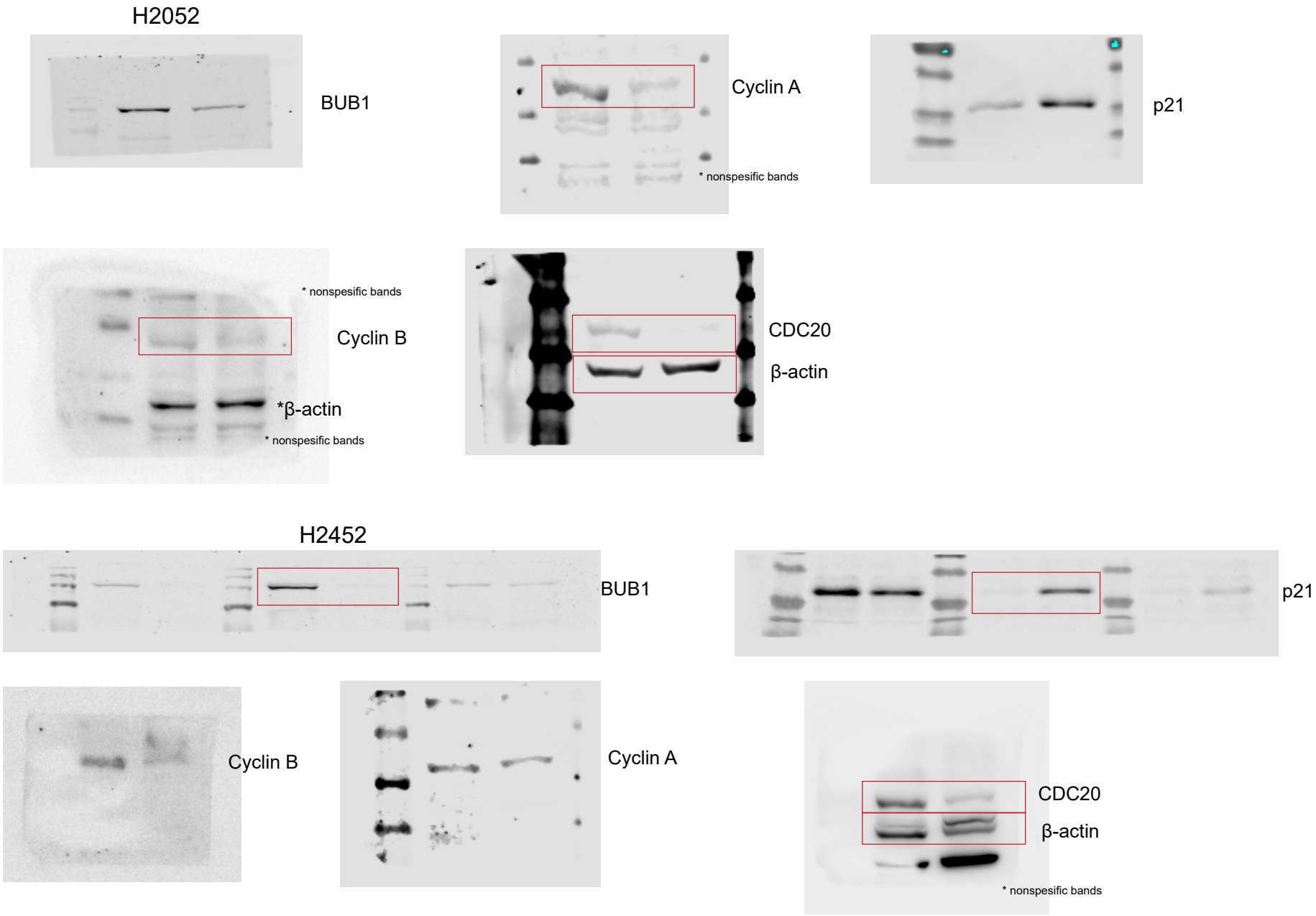

Fig. S9C

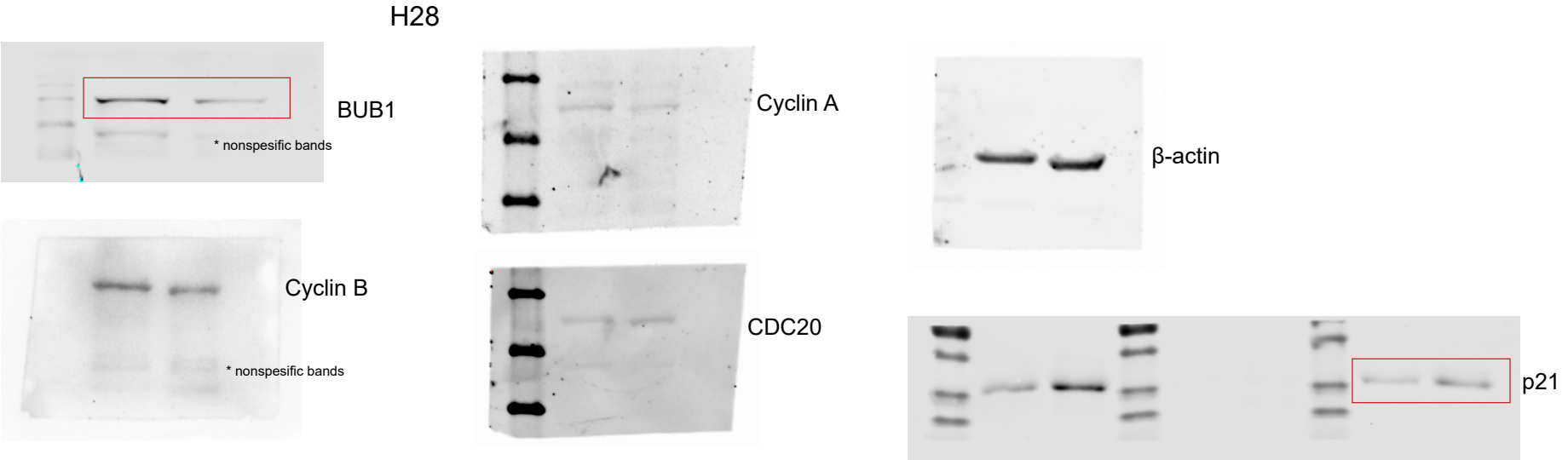

Supplement: Supplementary file 13 — Uncropped WB [file 41419_2025_7587_MOESM13_ESM.pdf]
